# ELF1-mediated transactivation of METTL3/YTHDF2 promotes nucleus pulposus cell senescence via m6A-dependent destabilization of E2F3 mRNA in intervertebral disc degeneration

**DOI:** 10.1038/s41420-025-02515-8

**Published:** 2025-06-04

**Authors:** Xiao-Wei Liu, Hao-Wei Xu, Shu-Bao Zhang, Yu-Yang Yi, Sheng-Jie Chang, Shan-Jin Wang

**Affiliations:** Department of Spinal Surgery, Shanghai East Hospital, School of Medicine, Tongji University, 200092, Shanghai, China

**Keywords:** Cell growth, Diagnostic markers

## Abstract

Intervertebral disc degeneration (IVDD) is a common pathology involving various degenerative diseases of the spine, with nucleus pulposus cell (NPC) senescence playing an important role in its pathogenesis. Transcriptional and epigenetic processes have been increasingly implicated in aging and longevity. E74-like factor 1 (ELF1) is a member of the erythroblast transformation specific family of proteins, which induce gene transcription by binding to gene promoters or enhancer sequences. However, the role of ELF1 in age-related diseases is unclear, with no reports of its involvement in NPC senescence or IVDD. ELF1 expression levels were assessed in human NP samples from IVDD patients, IVDD animal models, and naturally aged NP samples. Adeno-associated virus 5 (AAV5) vector-mediated Elf1 overexpressing mice and Elf1 knockout (KO) mice were used to investigate its role in NPC senescence and IVDD in vivo. The m6A methylase METTL3 and reading protein YTHDF2 were identified as downstream effectors of ELF1 using proteomic sequencing, RNA sequencing, ChIP-seq, promoter prediction, and binding analyses. MepRIP-qPCR, RNA pulldown, and double luciferase point mutation experiments revealed that METTL3 and YTHDF2 can recognize the m6A site on E2F3 mRNA, a key cell cycle gene. Finally, virtual screening techniques and various experiments were used to identify small molecule targets for ELF1 inhibition. ELF1 was found to drive m6A modification changes during NPC aging. The small molecule mycophenolate mofetil (MMF) could successfully target and inhibit ELF1 expression. In senescent NPCs, ELF1 can bind to the METTL3 and YTHDF2 gene promoter regions. Overexpressing METTL3 increased the E2F3 mRNA m6A modification abundance, while YTHDF2 was recruited to recognize this m6A site. This can accelerate the E2F3 mRNA degradation rate and ultimately lead to the onset of G1/S cell cycle arrest in NPC. For the first time, the transcription factor ELF1 has been identified as a novel regulator of NPC senescence and IVDD, which involves the ELF1-METTL3/YTHDF2-m6A-E2F3 axis. MMF, a small molecule designed to inhibit ELF1 and delay NPC senescence, was screened for the first time. This can potentially lead to new epigenetic therapeutic strategies for drug discovery and development for the clinical treatment of IVDD.

## Introduction

Lower back pain (LBP) is the most commonly observed health problem in clinical settings, causing severe disability and substantial social, economic, and medical costs worldwide [1, 2]. Intervertebral disc degeneration (IVDD) is the pathophysiological process of natural degeneration and aging of the intervertebral disc that is clinically associated with and a major cause of LBP. The intervertebral disc (IVD) is a fibrocartilaginous tissue consisting of a central, highly hydrated, gelatinous nucleus pulposus (NP), surrounded by a laminated annulus fibrosus (AF). The cartilaginous endplates (CEPs) define the upper and lower boundaries of the NP and AF [3–5]. Increasing research has suggested that the accumulation of senescent IVD cells, particularly NP cells (NPCs), in aging and degenerating discs may be a new hallmark and major etiological factor of IVDD [6, 7]. Furthermore, senescent NPCs secrete a variety of proinflammatory cytokines and proteases that can influence the local environment, leading to peripheral cellular senescence and tissue dysfunction [8, 9]. Therefore, targeting NPC senescence is a strategy with great clinical promise for effectively mitigating IVDD progression.

Repairing the integrity of the epigenome may be crucial for targeting drugs to reverse cellular aging, as recent studies have shown that epigenetic dysregulation is a central hallmark and driver of aging [10–12]. Some examples of epigenomic alterations include transcription factor binding, histone labeling, DNA methylation, N6-methyadenine (m6A) modifications, and non-coding RNA changes [13]. Age-related changes in transcription factors and the chromatin status are thought to be early drivers of senescence and important hubs that link together many of the features of aging [14, 15]. Individual transcription factors (pioneer factors) can mediate epigenetic changes while supporting the recruitment and activity of epigenetic regulators, thus exhibiting more persistent transcriptional regulatory changes [16]. Research has identified several transcription factors involved in human longevity and age-related diseases. These include the senescence-associated transcription factors p53 and MYC, both of which are key cell cycle regulators that are involved in the aging process in complex ways [17]. However, the pioneer transcription factors involved in the evolutionary control of epigenetic changes during NPC senescence have not yet been identified.

m6A is one of the most abundant RNA modifications in mammalian cells [18]. The m6A modification is a dynamic, reversible process that is initiated by m6A methyltransferases (‘writers’), released by m6A demethylases (‘erasers’), and recognized by reader proteins. Collectively, these proteins are involved in regulating RNA nuclear transport, splicing stability, translation, and RNA metabolism [19, 20]. m6A can regulate cellular senescence by modulating oxidative stress, telomere length, DNA damage, and the senescence-associated secretory phenotype (SASP) [21]. Altered m6A modification patterns play an important role in the progression of NPC senescence and IVDD, as m6A modifications have recently been shown to be significantly increased in tumor necrosis factor (TNF)-α-induced models of NPC senescence and IVDD [22–24]. Understanding the dynamics of m6A patterns during the natural aging of NP tissue will therefore be critical in identifying the major transcription factors responsible for such patterns.

E74 like factor 1 (ELF1) is a transcription factor belonging to the erythroblast transformation specific (ETS) family [25]. In recent years, studies have shown that ELF1 is involved in the development of numerous diseases through the regulation of various biological processes, such as cell proliferation, differentiation, apoptosis, and the immune response [26]. ELF1 can function in a wide range of organs and tissues, including nerve, brain, kidney, cardiovascular, and gastrointestinal tract tissues [27–30]. Furthermore, previous work has demonstrated that upregulated ELF1 in the lumbar dorsal root ganglion can bind to the NIS-lncRNA gene promoter to promote nerve injury, neuropathic pain symptoms, and hypersensitivity development [31]. However, no reports have indicated if ELF1 is a key transcription factor driving NPC senescence.

Expanding upon these previous research findings, the present study reports for the first time the role of ELF1 in NPC senescence, where this transcription factor can drive m6A modification changes. We also screened for small molecule drugs, finding that mycophenolate mofetil (MMF) can be used to inhibit ELF1 expression. Mechanistically, when ELF1 expression levels are elevated, it can bind to the DNA sequences of the genes encoding METTL3 (m6A methylation protein) and YTH structural domain family protein 2 (YTHDF2; m6A reader protein) to induce their transcription. Overexpression of METTL3 could increase the E2F3 m6A mRNA abundance, while recruiting the reader protein YTHDF2 to recognize the E2F3 m6A site accelerated mRNA degradation, leading to decreased E2F3 protein expression levels. Additionally, this causes G1/S phase arrest in NPCs, accelerating their senescence and ultimately leading to IVDD. Overall, our findings provide new insights into the molecular pathogenesis of NPC senescence in IVDD.

## Results

### ELF1 expression levels are significantly elevated in degenerating NP tissues and senescent NPCs

**Fig. 1:**
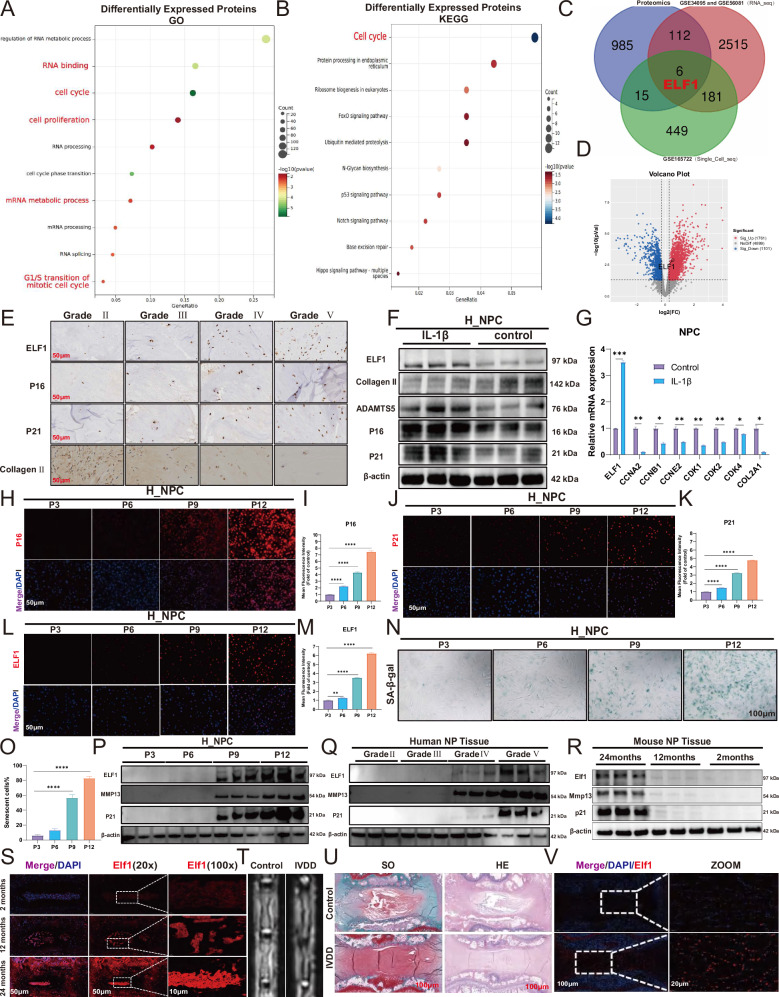
ELF1 expression levels in human degenerating NP tissues and senescent NPCs. **A** According to the Pfirrmann grading scale, grades I–III are mild degeneration (MDD) and grades IV–V are severe degeneration (SDD). Proteomics analysis of GO enrichment data of differentially expressed proteins in SDD and MDD NP tissues. **B** KEGG enrichment analysis of differentially expressed proteins in SDD and MDD NP tissues. **C** Venn diagram showing common gene intersections from the proteome sequencing, transcriptome sequencing, and single-cell group sequencing datasets. **D** Volcano plot showing the ELF1 mRNA expression levels in the combined transcriptomic dataset from GSE34095 and GSE56081. **E** Representative ELF1 immunohistochemistry staining images in human NP tissues of different Pfirrmann grades. **F** The protein expression levels of ELF1, Collagen II, ADAMTS5, P16, and P21 were detected after treating human-derived NPCs (H_NPC) with 10 ng/mL IL-1β for 48 h. **G** The mRNA expression levels of ELF1, CCNA2, CCNB1, CCNE2, CDK1, CDK2, CDK4, and COL2A1 were detected after treating H_NPC with 10 ng/mL IL-1β for 48 h. **H**–**M** Immunofluorescence data for the P16, P21, and ELF1 protein expression patterns in the H_NPC replicative senescence model; *N* = 3 biologically independent replicates. Scale bar=50 μm. **N**, **O** Representative SA-β-gal staining images and quantification of the H_NPC replicative senescence model; *N* = 3 biologically independent replicates. Scale bar=100 μm. **P** Western blot analysis of the protein expression levels of ELF1, MMP13, and P21 in the H_NPC replicative senescence model. **Q** Western blot analysis of the protein expression levels of ELF1, MMP13, and P21 in human NP tissues of different Pfirrmann grades. **R** Western blot analysis was used to detect the protein expression levels of Elf1, Mmp13, and p21 in the NP tissues of senescent mice at different months of age. **S** Immunofluorescence detection of the Elf1 protein expression levels in the NP tissues of senescent mice at different months of age. **T**, **U** MRI, H&E staining, and Safranin-O staining experiments were performed to validate the rat acupuncture IVDD model. **V** Immunofluorescence detection of the Elf1 protein expression levels in degenerated rat intervertebral disc tissues. The data are presented as the mean ± SD. One-way ANOVA was used for comparisons among multiple groups. *P < 0.05; **P < 0.01; ***P < 0.001; ****P < 0.0001.

We first selected degenerating human NP tissue samples for proteomics sequencing to investigate any key transcription factors that can drive NPC senescence. These included three samples with mild degeneration (MDD) and three with severe degeneration (SDD). GO and KEGG enrichment analyses showed that the differentially expressed proteins were mainly enriched in the cell cycle, cell proliferation, and mRNA metabolism (Fig. 1A, B). Bioinformatics analysis of single-cell sequencing data (GSE165722) was also used to analyze eight IVDD samples (MDD and SDD). In contrast to the MDD samples, Apoptosis, Focal adhesion, and Cellular senescence, were the main KEGG-enriched pathways in the NPC cluster of the SDD samples (Supplementary Fig. 1A–D). GO enrichment analysis revealed that the differentially expressed genes were mainly enriched in translation factor activity and RNA binding (Supplementary Fig. 1E). Additionally, the RNA-seq datasets GSE34095 and GSE56081 were merged, with the KEGG and GO enrichment analyses showing that the differentially expressed genes were mainly enriched in Cell cycle arrest and the G1/S transition mitotic cell cycle in the highly degenerated NP specimens (Supplementary Fig. 1F, G). The above multi-omics data demonstrate that both cell cycle arrest and cellular senescence play important roles in the IVDD process. Finally, we found that the transcription factor ELF1 is highly expressed in degenerated NP tissues after intersecting the differentially expressed genes from the proteomics sequencing, transcriptome sequencing, and single-cell sequencing datasets (Fig. 1C). Further volcano mapping revealed high ELF1 mRNA expression levels in the highly degenerated specimens (Fig. 1D). In addition, multi-organ single-cell sequencing data (https://twc-stanford.shinyapps.io/maca/) [32] from mice of different ages showed significantly upregulated Elf1 expression levels in aging bones, brain, gonadal adipose tissue, kidneys, limb muscles (tibialis anterior), liver, lung, mesenteric adipose tissue (MAT), pancreas, skin, and small intestine (duodenum) (Supplementary Fig. 2), suggesting that Elf1 has an important role in senescence in mice. We also established a replicative senescence model and H_2_O_2_ (200 µM) senescence model in rat NPCs (R_NPC) (Supplementary Fig. 3A, B). KEGG and GO enrichment analyses showed that the differentially expressed genes in the senescence model were mainly enriched in the Cell Cycle, Cellular senescence, and DNA replication (Supplementary Fig. 3C–F). E2F family proteins, which are involved in the G1 (pre-DNA synthesis) to S (DNA synthesis) cell cycle checkpoint, were downregulated in the senescence model (Supplementary Fig. 3G). The Elf1 expression levels were significantly upregulated in both R_NPC senescence models, consistent with the proteomic sequencing results described above (Supplementary Fig. 3H). Immunohistochemistry (IHC) staining results confirmed that the ELF1 protein expression levels increased with IVDD severity (Fig. 1E). Our in vitro model of IVDD using interleukin (IL)-1β (10 ng/mL) treatment in human-derived NPCs (H_NPC) revealed significantly elevated ELF1 protein and mRNA expression levels (Fig. 1F, G). In addition, the H_NPC replicative senescence model showed a gradual increase in P21 and P16 expression levels (Fig. 1H–K), with the ELF1 protein expression levels being synchronously increased (Fig. 1L, M). SA-β-gal assays confirmed that the H_NPC model showed a gradual increase in senescent cells with the number of passages (Fig. 1N, O). ELF1 protein expression levels were found to be significantly increased in both the H_NPC replicative senescence model and severely degenerated human NP tissues (Fig. 1P, Q). We further validated the Elf1 expression levels in NP tissues from naturally aged mice. Magnetic resonance imaging (MRI), hematoxylin and eosin (H&E) staining, Safranin-O staining, and histological scoring confirmed that NP tissue degeneration progressively worsened as the mice aged (Supplementary Fig. 4A–F). The immunofluorescence results showed that the p16 and p21 protein expression levels were significantly increased in the senescent NP tissues, while the collagen II and cyclin D1 protein expression levels were significantly lower (Supplementary Fig. 4G–J). Western blot and immunofluorescence analyses confirmed that Elf1 protein expression is significantly upregulated in senescent mouse NP tissues (Fig. 1R, S). Immunofluorescence results further showed that Elf1 protein expression is also significantly upregulated in degenerated rat IVD (Fig. 1T–V). Therefore, the transcription factor ELF1 was found to be highly expressed in degenerating NP tissues following validation of several in vitro and in vivo experiments.

### Reducing ELF1 expression levels could significantly inhibit NPC senescence

**Fig. 2:**
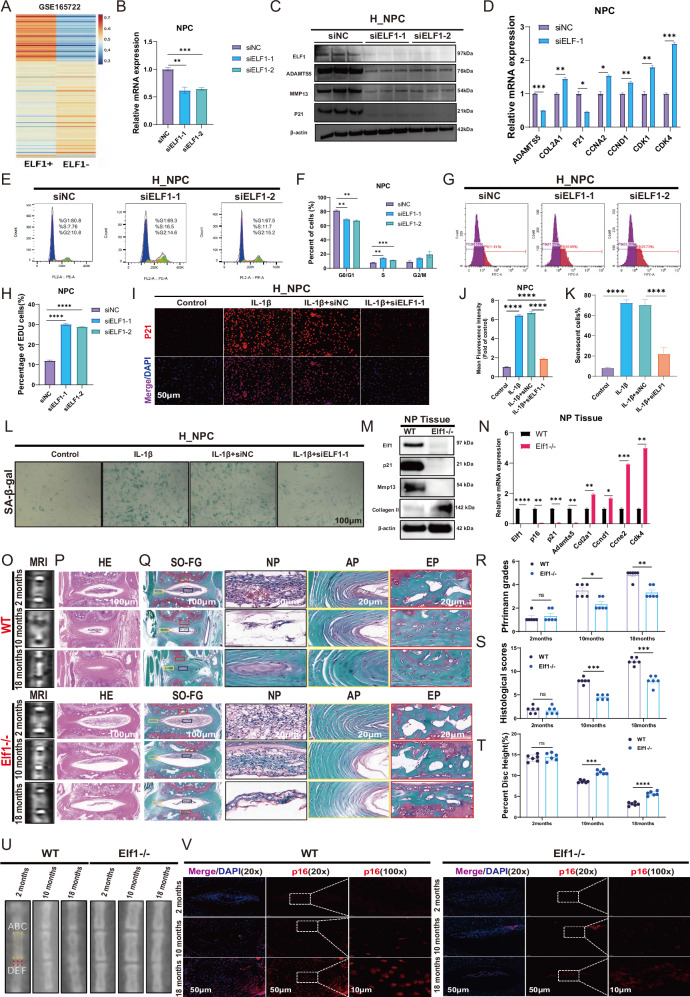
Reducing the expression of ELF1 inhibits the senescence of NPC and delays the IVDD. **A** Heatmap of differentially expressed genes in ELF1^+^ cell subpopulations vs. ELF1^-^ cell subpopulations. **B** The knockdown efficiency of ELF1 siRNA was detected by qPCR. **C** Western blot detection of protein expression levels of ADAMTS5, MMP13 and P21 in H_NPC after knockdown of ELF1. **D** The mRNA expression levels of extracellular matrix genes and cell cycle genes were detected by qPCR after H_NPC knockdown of ELF1. **E**, **F** Flow cytometry was used to detect the cell cycle progression of H_NPC after knockdown of ELF1. **G**, **H** EDU assays the DNA replication capacity of H_NPC after knockdown of ELF1. **I**, **J** Immunofluorescence was used to detect the protein expression level of P21 after the addition of ELF1 siRNA after 10 ng/ml IL-1β treatment of H_NPC for 48 h; *N* = 3 biologically independent replicates; Scale bar = 50 μm. **K**, **L** Representative images of SA-β-gal staining and quantification after 10 ng/ml IL-1β treatment of H_NPC for 48 h followed by the addition of ELF1 siRNA; *N* = 3 biologically independent replicates. Scale bar = 100 μm. **M** Western blot for protein expression levels of p21, Mmp13 and Collagen II in NP tissue after Elf1 KO. **N** The mRNA expression levels of extracellular matrix genes and cell cycle genes were detected by qPCR in NP tissues after Elf1 KO. **O**, **R** MRI detection of T2-weighted signal intensity in the intervertebral discs of WT and Elf1 KO naturally aging mice of different months of age; *N* = 6; **P**, **Q**, **S** H&E and Safranin-O staining of intervertebral discs from WT and Elf1 KO naturally aging mice at different months of age; *N* = 6; Scale bar = 100 μm and 20 μm. **T**, **U** Representative x-ray film images of intervertebral disc heights in WT and Elf1 KO naturally aging mice at different months of age; *N* = 6. **V** Immunofluorescence detection of the expression level of p16 in the NP tissues of intervertebral discs of WT and Elf1 KO naturally aging mice at different months of age. Data presented as mean ± SD. One-way ANOVA was used for comparison among multiple groups. *P < 0.05; **P < 0.01; ***P < 0.001; ****P < 0.0001.

We then performed differential gene analyses of the ELF1^+^ and ELF1^-^ sub-groups among the NPC sub-populations in the single-cell data (Fig. 2A). GO enrichment analysis of differential genes mainly enriched in Cell cycle G1/S phase transition, Cellular senescence, RNA stabilization, RNA N6-methyladenosine methyltransferase complex (Supplementary Fig. 4K). KEGG enrichment analysis of differential genes mainly enriched Cell cycle, RNA degadation, Longevity regulating pathway (Supplementary Fig. 4L). After confirming efficient ELF1 knockdown, significantly reduced expression levels of P21, ADAMTS5, and MMP13 were observed (Fig. 2B, C). The qPCR results showed that knocking down ELF1 expression significantly reduced the ADAMTS5 and P21 mRNA expression levels, while promoting the mRNA expression levels of COL2A1, CCNA2, CCND1 CDK1, and CDK4 (Fig. 2D). Flow cytometry assay data revealed that reduced ELF1 expression levels could promote the H_NPC G1/S phase transition (Fig. 2E, F). EDU experiments indicated that ELF1 knockdown promoted DNA replication and cell proliferation in the H_NPC model (Fig. 2G, H), while immunofluorescence results showed that it significantly reduced the IL-1β-induced elevation of P21 protein expression levels (Fig. 2I, J). SA-β-gal analysis confirmed that ELF1 knockdown also significantly reduced IL-1β-induced H_NPC senescence (Fig. 2K, L). Furthermore, the Elf1 protein expression levels gradually increased in the R_NPC replicative senescence model (Supplementary Fig. 5A, B). Reducing Elf1 expression delayed R_NPC senescence by inhibiting IL-1β-induced senescent protein upregulation and extracellular matrix degradation (Supplementary Fig. 5C–G). KEGG enrichment analyses by RNA-seq after Elf1 knockdown in the R_NPC model showed that the differentially expressed genes were mainly enriched in the mitotic cell cycle (Supplementary Fig. 5H, I). Next, we constructed Elf1 knockout (KO) mice to validate the critical role of Elf1 in IVDD. In Elf1 KO NP tissues, p21 and Mmp13 were expressed at significantly lower levels, while collagen II expression patterns were significantly higher (Fig. 2M). The qPCR results showed a significant decrease in the mRNA expression levels of p16, p21, and Adamts5, along with significantly increased mRNA expression levels of the cell cycle genes Ccnd1, Ccne2, and Cdk4, in the Elf1 KO NP tissues (Fig. 2N). The MRI results showed a higher T2-weighted signal intensity in the intervertebral discs of Elf1 KO mice compared with in those of their littermate wild-type (WT) mice (Fig. 2O, R). H&E and Safranin-O staining showed that lower Elf1 expression levels resulted in a significantly increased number of NP tissues and delayed IVDD (Fig. 2P, Q, S). The X-ray results indicated that the Elf1 KO increased the intervertebral disc space and delayed the onset of IVDD (Fig. 2T, U). Immunofluorescence assay data demonstrated significantly reduced senescent protein p16 expression levels in the Elf1 KO NP tissues (Fig. 2V). In summary, both in vivo and in vitro experiments confirmed that reducing ELF1 expression could significantly inhibit NPC cell senescence, thus delaying IVDD.

### METTL3 is involved in the regulation of NPC senescence as a downstream molecule of ELF1

**Fig. 3:**
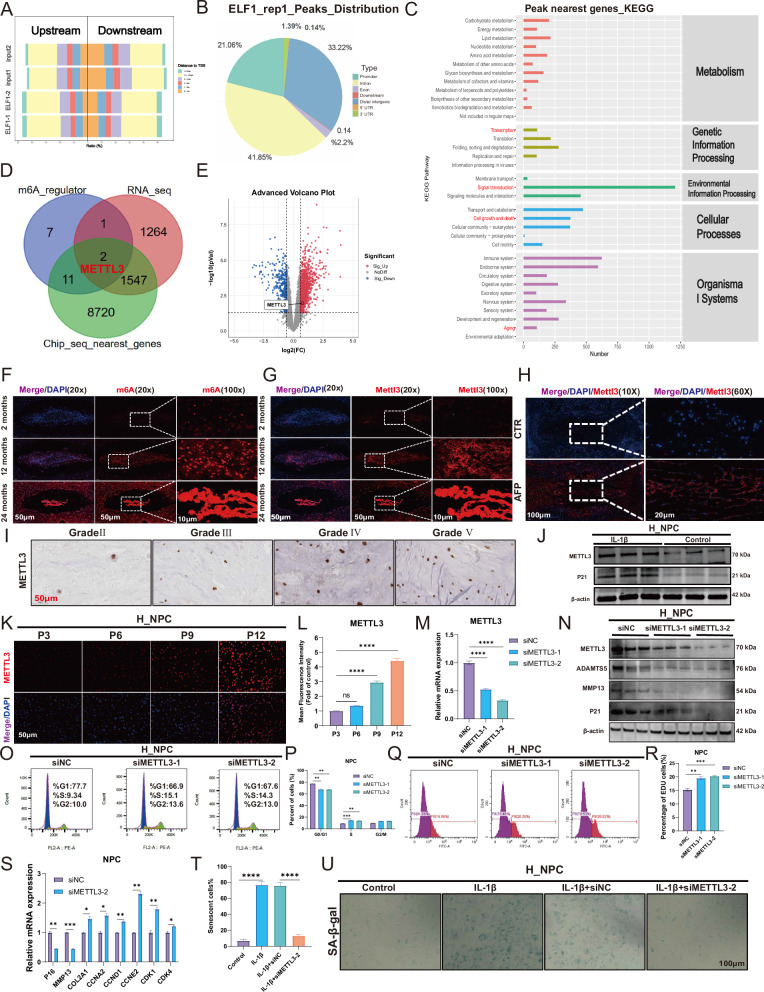
METTL3 is involved in the regulation of NPC senescence as a downstream of ELF1. **A** Distribution of Peak and TSS distances. Vertical coordinates are samples, different colors represent Peak in different intervals from TSS, horizontal coordinates are the proportion of Peak in different intervals to the total Peak, Upstream means Peak is upstream of TSS, Downstream means Peak is downstream of TSS. **B** Peak binding spectrum of TSS region. When the Peak coverage frequency of the TSS region is in the shape of a symmetrically distributed peak, it indicates that the target protein is concentrated in the TSS region. **C** Peak Neighbouring gene KEGG taxonomic annotations. **D** Venn diagram shows the intersection of m6A regulator with RNA-seq differential gene and Chip_seq_nearest_genes gene. **E** Volcano plot demonstrating the expression level of METTL3 in RNA-seq. **F**, **G** Immunofluorescence detection of m6A modification and Mettl3 expression levels in medullary tissues of naturally aging mice. **H** Immunofluorescence detection of Mettl3 expression levels in NP tissues in a rat model of acupuncture IVDD. **I** Immunohistochemical detection of METTL3 expression in human NP tissue. **J** The expression levels of METTL3 and P21 were detected 48 h after 10 ng/ml IL-1β treatment. **K**, **L** Immunofluorescence detection of METTL3 expression levels in the NPC replicative senescence model. **M** The knockdown efficiency of METTL3 was detected by qPCR. **N** The knockdown efficiency of METTL3 was detected by Western Blot. **O**, **P** Flow cytometry to detect the effect of knockdown of METTL3 on NPC cell cycle progression. **Q**, **R** EDU assays the effect of knockdown of METTL3 on NPC DNA replication. **S** Effect of knockdown of METTL3 on mRNA expression of cell cycle genes detected by qPCR. **T**, **U** SA-β-gal staining was used to detect the effect of knockdown of METTL3 on NPC senescence after 48 h of 10 ng/ml IL-1β treatment. Data presented as mean ± SD. One-way ANOVA was used for comparison among multiple groups. *P < 0.05; **P < 0.01; ***P < 0.001; ****P < 0.0001.

We performed ChIP-seq analysis using an anti-ELF1 antibody in H_NPC to identify ELF1 downstream target genes. The distance distribution of peak to transcription start site (TSS) was analyzed first, where “Upstream” means that the peak is upstream of the TSS and “Downstream” means that the peak is downstream of the TSS (Fig. 3A). The ChIP-seq results showed that the peak coverage frequency of the TSS region was symmetrically distributed in shape, suggesting that the ELF1 protein binds centrally to the TSS region, thereby regulating the transcription of neighboring genes (Fig. 3B). KEGG enrichment analysis showed that the peak neighboring genes are mainly involved in transcription, replication and repair, cell growth and death, and aging (Fig. 3C). Examining the ELF1 ChIP-seq public database suggested that ELF1 was primarily distributed on the promoter element (Supplementary Fig. 6A–F). KEGG functional enrichment analysis of the peak annotated genes revealed that ELF1 is mainly involved in the mRNA surveillance pathway, Cell cycle, and Cellular senescence (Supplementary Fig. 6G–L). Methylated protease METLL3, which is involved in the m6A modification, was found to be a key target of ELF1 transcriptional regulation. This was achieved by intersecting the m6A regulator with the human IVD RNA-seq differentially expressed genes and ChIP-seq _nearest_genes (Fig. 3D). The RNA-seq results suggested that METTL3 is highly expressed in severely degenerated human NP tissues (Fig. 3E). We further analyzed Me-RIP-seq data (GSE169484) [24] to compare the m6A modifications in transcripts from normal and senescent H_NPC. From m6A-seq data, we identified 4,553 and 3,542 m6A peaks in 3,765 and 2,999 m6A-modified transcripts from control and senescent NPCs, respectively. Peak distribution analysis showed that the m6A sites were enriched in both exons, the 3’ untranslated region (3’ UTR), and coding sequence (CDS), with the highest enrichment of m6A residues located near the stop codon (Supplementary Fig. 7A–H). GO enrichment analysis showed that the m6A-modified genes were mainly involved in cell cycle arrest, mRNA stabilization, and RNA metabolic process (Supplementary Fig. 7I). The above results further suggested that the m6A modification plays an important role in H_NPC senescence. Additionally, the m6A abundance and Mettl3 expression levels were significantly elevated in naturally senescent NP tissues (Fig. 3F, G). Mettl3 expression was also significantly elevated in the rat acupuncture IVDD model (Fig. 3H). In human degenerating NP tissues, the METTL3 expression levels increased with higher Pfirrmann grading (Fig. 3I). METTL3 expression patterns also significantly increased following IL-1β treatment of H_NPC (Fig. 3J). Our immunofluorescence results also showed that the METTL3 protein expression levels gradually increased with progressive H_NPC senescence (Fig. 3K, L). METTL3 knockdown significantly reduced the expression levels of the extracellular matrix proteins ADAMTS5, MMP13, and senescent protein P21 (Fig. 3M, N). Flow cytometry data indicated that knocking down METTL3 expression could promote the G1/S phase transition (Fig. 3O, P). EDU experiments further demonstrated that METTL3 knockdown could support H_NPC proliferation (Fig. 3Q, R). Using qPCR, we found that METTL3 knockdown led to lower mRNA expression levels of P16 and MMP13, but increased those of COL2A1, CCNA2, CCND1, CCNE2, CDK1, and CDK4 (Fig. 3S). Furthermore, as confirmed by SA-β-gal staining, METTL3 knockdown significantly delayed IL-1β-induced H_NPC senescence (Fig. 3T, U). In addition, in the rat NPC replicative senescence model, immunofluorescence assays showed a significant upregulation of Mettl3 protein expression (Supplementary Fig. 8A, B). Knocking down Mettl3 reduced the expression levels of p16 and p21, promoted the expression of cyclin d1, and inhibited the IL-1β-induced increase of p16 protein expression (Supplementary Fig. 8C–G). Gene set enrichment analysis (GSEA) of RNA-seq data following Mettl3 knockdown in R_NPC indicated differential gene involvement in the G1_to_S_cell cycle pathway (Supplementary Fig. 8H–J). GO and KEGG enrichment analyses further revealed differential gene involvement in RNA biosynthesis and cellular senescence (Supplementary Fig. 8K, L), with the upregulated differentially expressed genes also mainly involved in RNA biosynthesis and cellular senescence (Supplementary Fig. 8M, N). Moreover, Mettl3 knockdown significantly delayed IL-1β-induced R_NPC senescence (Supplementary Fig. 8O, P). Overall, the m6A methylated protein METTL3, as a downstream target of ELF1, is involved in the NPC senescence process.

### ELF1 transcriptionally regulates METTL3 to promote IVDD

**Fig. 4:**
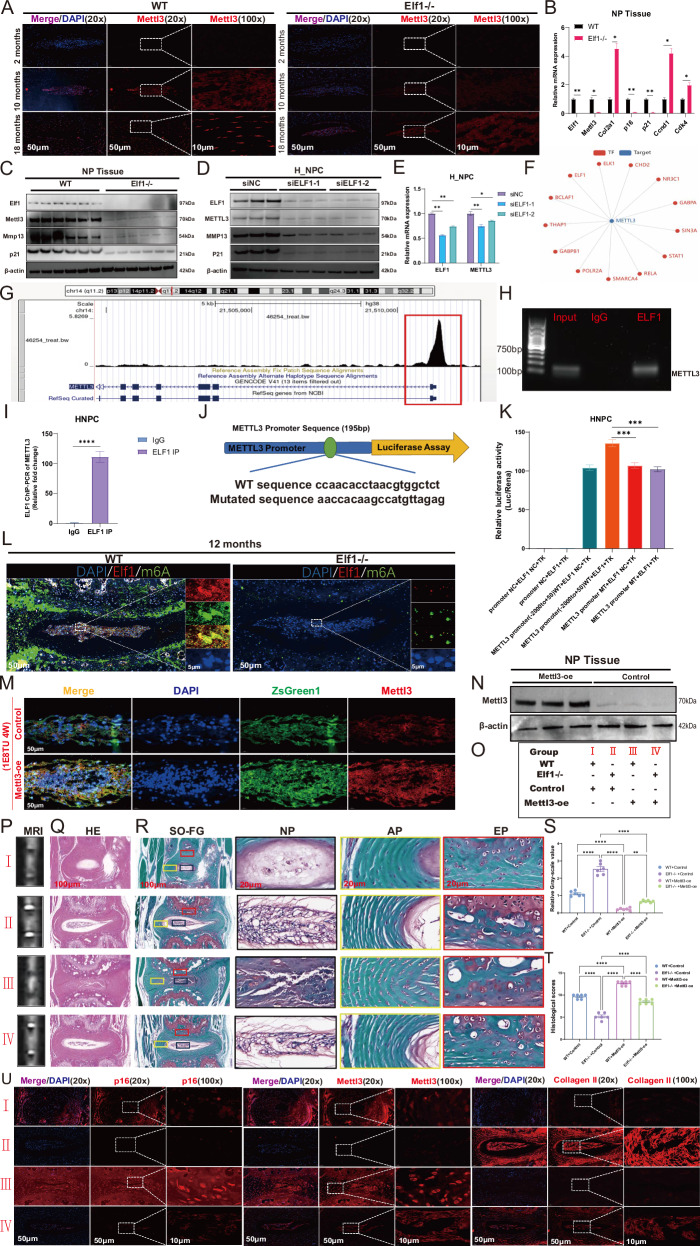
ELF1-mediated transcriptional regulation of METTL3 accelerates IVDD. **A** Immunofluorescence detection of the Mettl3 protein expression patterns in mouse NP tissues after Elf1 knockout (KO). **B** The mRNA expression levels of Mettl3 and cellular senescence-related proteins in mouse NP tissues after Elf1 KO were detected by qPCR. **C** Western blot analysis of the protein expression levels of Mettl3, Mmp13, and p21 in the NP tissues of mice after Elf1 KO. **D** Western blot analysis of the protein expression levels of Mettl3, Mmp13, and p21 following ELF1 knockdown in H_NPC. **E** The Mettl3 mRNA expression levels were detected using qPCR following ELF1 knockdown in H_NPC. **F** The single-cell sequencing transcription factor database was used to validate the transcription factors upstream of METTL3. **G** An ELF1 binding peak in the METTL3 promoter region was detected using the open ChIP-seq database. **H** The METTL3 promoter sequences were determined by examining the ELF1 immunoprecipitates using PCR. **I** ELF1 binding to the METTL3 promoter in H_NPC was confirmed by ChIP-PCR. **J** The human METTL3 promoter region contains ELF1-like elements. **K** Luciferase activity, with expression driven by the METTL3 promoter, was more pronounced following ELF1 overexpression. **L** Immunofluorescence detection of Elf1 expression and m6A modification levels and co-localization in mouse NP tissues after Elf1 KO. **M**, **N** Immunofluorescence and western blot analyses were used to detect the Mettl3 protein expression levels in mouse NP tissues injected with a Mettl3-overexpressing lentivirus. **O** Subgroups of Mettl3-overexpressing lentivirus injected into the NP tissues of Elf1 KO mice. **P**, **S** MRI detection of the T2-weighted signal intensity in intervertebral discs after injecting the Mettl3-overexpressing lentivirus into the NP tissues of wild-type (WT) and Elf1 KO mice. *N* = 6. **Q**, **R**, **T** H&E and Safranin-O staining of intervertebral discs after injecting the Mettl3-overexpressing lentivirus into the NP tissues of WT and Elf1 KO mice; *N* = 6; Scale bars = 100 μm and 20 μm. **U** Immunofluorescence detection of the protein expression levels of p16, Mettl3, and Collagen II in the NP tissues of WT and Elf1 KO mice injected with the Mettl3-overexpressing lentivirus. The data are presented as the mean ± SD. One-way ANOVA was used for comparisons among multiple groups. *P < 0.05; **P < 0.01; ***P < 0.001; ****P < 0.0001.

Following Elf1 KO, we observed significantly decreased Mettl3 expression levels in the mouse NP tissues (Fig. 4A). The qPCR results showed that the mRNA levels of Mettl3, p16, and p21 were significantly downregulated, while those of Col2a1, Ccnd1, and Cdk4 were significantly increased, in the Elf1 KO mouse NP tissues (Fig. 4B). Western blot analysis showed significantly decreased Mettl3 protein expression levels in the NP tissues of Elf1 KO mice (Fig. 4C). Knocking down ELF1 expression in H_NPC significantly reduced the METTL3 and P21 protein expression levels (Fig. 4D), with qPCR data indicating that METTL3 expression also decreased at the mRNA level (Fig. 4E). In addition, a transcription factor database based on single-cell sequencing data further identified METTL3 as a potential latent target of ELF1 (Fig. 4F). Public ChIP-seq datasets further identified the METTL3 promoter region as having abundant ELF1 protein binding peaks (Fig. 4G). In H_NPC, we identified the ELF1 binding site in the METTL3 promoter region and designed ChIP primers. We then used PCR to further amplify the METTL3 promoter fragment using human genomic DNA as a template. After immunoprecipitation with an anti-ELF1 antibody, clear DNA amplification was observed (Fig. 4H). ChIP-qPCR further demonstrated that ELF1 can bind to the METTL3 promoter fragment (Fig. 4I). Finally, binding site assays using a luciferase reporter gene revealed that ELF1 binds to a site in the METTL3 promoter, with mutations in this site resulting in no promotion of METTL3 expression (Fig. 4J, K). Elf1 and m6A were co-localized in mouse NP tissues, with Elf1 being highly expressed in synchrony with m6A in senescent NP tissues. However, Elf1 KO significantly reduced the overall m6A levels in NP tissues (Fig. 4L). These results further suggested that Elf1 can drive changes in m6A abundance in NP tissues by regulating Mettl3. In addition, we injected a lentivirus to overexpress Mettl3 in the NP tissues of Elf1 KO mice for rescue experiments. Immunofluorescence and western blot experiments verified that Mettl3 can be efficiently overexpressed in these tissues (Fig. 4M, N). Mouse IVDs displayed a lower T2-weighted signal intensity on MRI after two months of sustained Mettl3 overexpression in 10-month-old NP tissues (Fig. 4O, P, S). H&E and Safranin-O staining showed that Elf1 KO significantly delayed the reduction in the number of NP tissues resulting from Mettl3 overexpression (Fig. 4Q, R, T). Immunofluorescence assays demonstrated that overexpressing Mettl3 could accelerate NP tissue senescence by promoting p16 expression and inhibiting collagen II expression. In contrast, Elf1 KO reversed the increased senescent protein expression and decreased extracellular matrix protein expression caused by Mettl3 overexpression (Fig. 4U). Taken together, ELF1 can directly bind to the METTL3 promoter region to accelerate IVDD.

### METTL3 accelerates NPC senescence by inhibiting expression of the key cell cycle gene E2F3

**Fig. 5:**
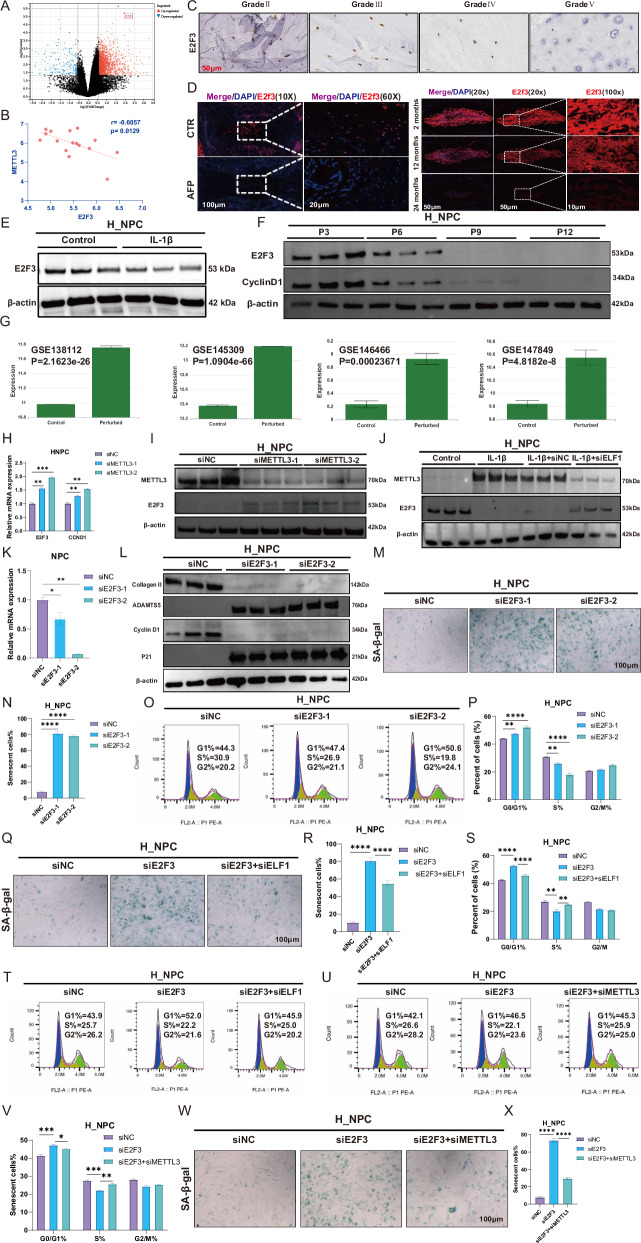
METTL3 inhibits expression of the key cell cycle gene E2F3 to accelerate NPC senescence. **A** Volcano map demonstrating that the E2f3 expression levels are significantly upregulated following Mettl3 knockdown. **B** Correlation analysis of the expression levels of METTL3 and E2F3 in RNA-seq data from human NP tissues. **C** Immunohistochemistry detection of E2F3 protein expression levels in human NP tissues. **D** Immunofluorescence detection of the E2f3 protein expression levels in NP tissues from the rat IVDD and mouse natural aging models. **E** Western blot analysis was used to detect the E2F3 protein expression levels after 10 ng/mL IL-1β treatment for 48 h. **F** Western blot analysis of the E2F3 protein expression levels in the H_NPC replicative senescence model. **G** The E2F3 expression levels were examined in the METTL3 KO database. **H** The E2F3 mRNA expression levels were detected by qPCR following METTL3 knockdown in H_NPC. **I** The E2F3 protein expression levels were detected by western blot analysis following METTL3 knockdown. **J** Western blot analysis was used to detect the effects of ELF1 knockdown on the E2F3 protein expression levels after 10 ng/mL IL-1β treatment for 48 h. **K** The E2F3 knockdown efficiency was detected by qPCR. **L** Western blot analysis was used to detect the protein expression levels of Collagen II, ADAMTS5, Cyclin D1, and P21 following E2F3 knockdown. **M**, **N** SA-β-gal staining was used to detect the effects of E2F3 knockdown on H_NPC senescence. **O**, **P** Flow cytometry was performed to detect the effects of E2F3 knockdown on H_NPC cell cycle progression. **Q**, **R** SA-β-gal staining was used to detect the effects of simultaneous E2F3/ELF1 knockdown on H_NPC senescence. **S**, **T** Flow cytometry was used to detect the effects of simultaneous E2F3/ELF1 knockdown on H_NPC cell cycle progression. **U**, **V** Flow cytometry was used to detect the effects of simultaneous E2F3/METTL3 knockdown on the medullary cell cycle. **W**, **X** SA-β-gal staining was used to detect the effects of simultaneous E2F3/METTL3 knockdown on H_NPC senescence. The data are presented as the mean ± SD. One-way ANOVA was used for comparisons among multiple groups. *P < 0.05; **P < 0.01; ***P < 0.001; ****P < 0.0001.

Decreased expression levels of the genes encoding E2F family proteins were found following RNA-seq analysis of the R_NPC senescence model. The key cell cycle gene E2f3 was significantly upregulated after Mettl3 knockdown (Fig. 5A). We also observed a significant negative correlation between the expression levels of METTL3 and E2F3 in the human NP tissue RNA-seq data (Fig. 5B), with E2F3 expression levels decreasing with an increasing IVDD grade (Fig. 5C). E2f3 protein expression levels were significantly decreased in NP tissues from both the rat acupuncture IVDD model and mouse natural aging model (Fig. 5D). E2F3 expression levels were also significantly decreased in the IL-1β-induced in vitro IVDD model and replicative aging model (Fig. 5E, F). In addition, in four METTL3 KO datasets, METTL3 knockdown was found to significantly promote E2F3 mRNA expression (Fig. 5G). Our qPCR data showed that knocking down METTL3 significantly promoted the mRNA expression of E2F3 and CCND1 (Fig. 5H), with western blot analysis confirming that this also significantly increased E2F3 expression at the protein level (Fig. 5I). Furthermore, ELF1 knockdown inhibited METTL3 expression and promoted E2F3 expression (Fig. 5J), while E2F3 knockdown significantly promoted the expression of ADAMTS5 and P21 and inhibited the expression of Collagen II and Cyclin D1 (Fig. 5K, L). SA-β-gal staining revealed that knocking down E2F3 significantly increased the number of senescent cells, while significantly inhibiting the H_NPC cell cycle G1/S transition (Fig. 5M–P). The SA-β-gal staining data also confirmed that inhibiting ELF1 expression could significantly delay the NPC senescence, as well as reverse the cell cycle G1/S arrest, resulting from E2F3 knockdown (Fig. 5Q–T). Furthermore, inhibiting METTL3 expression reversed the cell cycle G1/S arrest and delayed the NPC senescence caused by E2F3 knockdown (Fig. 5U–X). Taken together, ELF1 can promote METTL3 expression and inhibit E2F3 expression to accelerate H_NPC senescence.

### METTL3 accelerates E2F3 mRNA degradation by increasing the abundance of m6A modifications

**Fig. 6:**
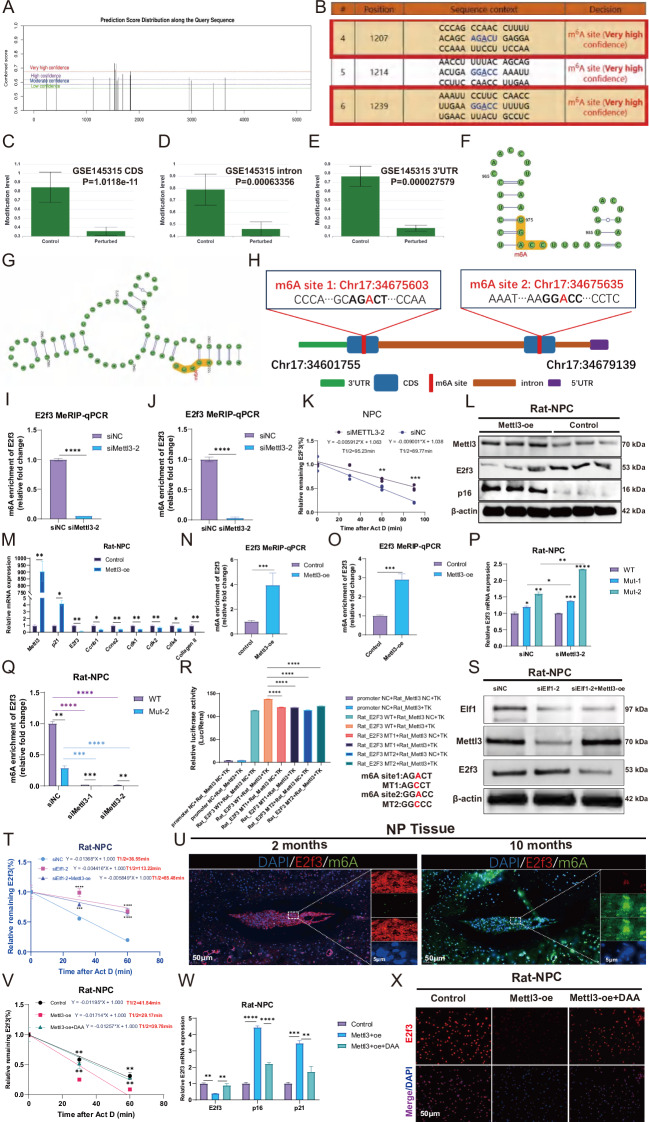
METTL3 increases E2F3 mRNA m6A modification to accelerate E2F3 mRNA degradation. **A**, **B** SRAMP was used to predict the m6A sites of the E2f3 mRNA. **C**–**E** The GSE145315 database shows the E2f3 mRNA m6A modification sites with Mettl3 knockout (KO). **F**–**H** The E2f3 mRNA m6A sites predicted with high confidence. **I**, **J** The E2f3 mRNA m6A modification abundance was determined by MeRIP-qPCR following Mettl3 knockdown. **K** The E2f3 mRNA degradation rate following Mettl3 knockdown was determined by qPCR. **L**, **M** Western blot and qPCR analyses were used to detect the protein and mRNA expression levels of E2f3 and p16 following Mettl3 overexpression. **N**, **O** The E2f3 mRNA m6A modification abundance was detected using MeRIP-qPCR following Mettl3 overexpression. **P** The E2f3 mRNA expression levels with wild-type (WT) or site-mutant sequences with or without Mettl3 knockdown. **Q** The E2f3 mRNA m6A modification abundance in WT or site-mutant sequences with or without Mettl3 knockdown. **R** Dual luciferase reporter gene assays demonstrating the binding capacity of METTL3 to E2F3. **S** The E2f3 protein expression levels were detected following Elf1 knockdown and Mettl3 overexpression in R_NPC using western blot analysis. **T** The effects of Elf1 knockdown and Mettl3 overexpression on the E2f3 mRNA degradation rate were detected by qPCR. **U** The co-localization of E2f3 and m6A in NP tissues of senescent mice were detected by immunofluorescence. **V** The effects of Mettl3 overexpression and m6A inhibitor (DAA, 50 μM) treatment on the E2f3 mRNA degradation rate. **W**, **X** qPCR and immunofluorescence analyses were used to detect the effects of Mettl3 overexpression followed by DAA treatment on E2f3 mRNA and protein expression levels. The data are presented as the mean ± SD. One-way ANOVA was used for comparisons among multiple groups. *P < 0.05; **P < 0.01; ***P < 0.001; ****P < 0.0001.

Because we observed that E2f3 expression was significantly elevated in the RNA-seq data of R_NPC with Mettl3 knockdown, we next explored how Mettl3 can regulate E2f3 expression in this model. We found multiple m6A sites on the E2f3 mRNA using the SRAMP database (Fig. 6A, B). We further analyzed the GSE145315 dataset and found that Mettl3 KO resulted in significantly reduced m6A modifications in the E2f3 mRNA CDS, intron, and 3’ UTR regions (Fig. 6C–E). We selected the m6A modification site on E2f3 with high confidence for validation in R_NPC (Fig. 6F–H). MepRIP-qPCR experiments showed that Mettl3 knockdown significantly reduced the abundance of two m6A sites on E2f3 mRNA (Fig. 6I, J), while enhancing its mRNA stability (Fig. 6K). Western blot and qPCR results demonstrated that overexpressing Mettl3 significantly reduced the E2f3 protein and mRNA expression levels (Fig. 6L, M). MepRIP-qPCR data indicated that Mettl3 overexpression significantly increased the abundance of two m6A sites on E2f3 mRNA (Fig. 6N, O). Mettl3 knockdown followed by m6A site mutation significantly increased the E2f3 mRNA expression levels (Fig. 6P), with MepRIP-qPCR assays demonstrating that this also significantly reduced the E2f3 mRNA m6A abundance (Fig. 6Q). Dual luciferase assays resulted in significantly reduced binding of Mettl3 to E2f3 after both E2f3 mRNA m6A sites were mutated (Fig. 6R). Western blot analysis showed that overexpressing Mettl3 significantly reduced the induction of E2f3 protein expression following Elf1 knockdown (Fig. 6S). Elf1 knockdown also slowed the E2f3 mRNA degradation rates, whereas Mettl3 overexpression accelerated this degradation (Fig. 6T). Immunofluorescence co-localization analysis revealed a significant decrease in E2f3 expression with increased m6A abundance in senescent NP tissues (Fig. 6U). E2f3 mRNA stability was reduced after Mettl3 overexpression, but was significantly enhanced after treatment with a specific m6A methylation inhibitor (3-Deazaadenosine, DAA) (Fig. 6V). The E2f3 mRNA and protein expression levels were significantly upregulated after DAA treatment (Fig. 6W, X). Taken together, Mettl3 can increase the abundance of the E2f3 m6A site and thereby promote the degradation of E2f3 mRNA.

### YTHDF2 accelerates NPC senescence by promoting E2F3 mRNA degradation through recognition of the m6A site

**Fig. 7:**
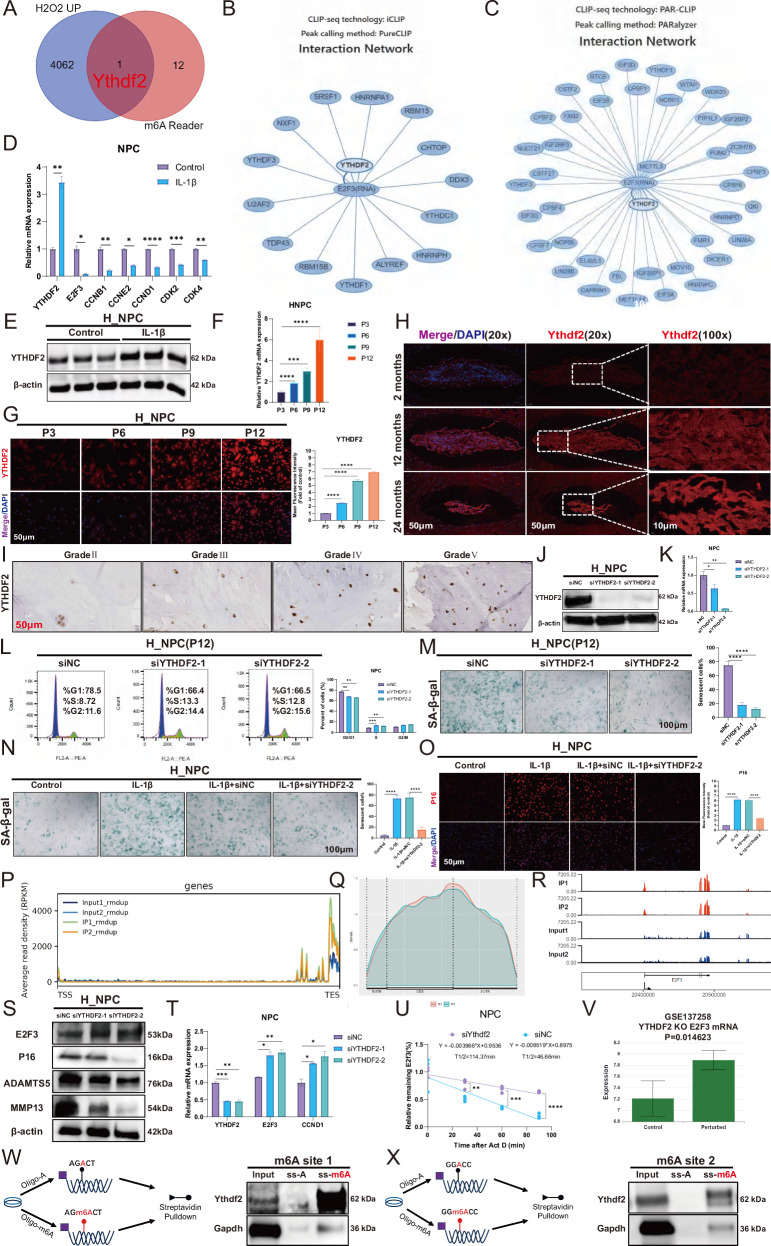
YTHDF2 accelerates NPC senescence by promoting E2F3 mRNA degradation by recognising the m6A site. **A** Venn diagram shows the intersection of differential genes and m6A reader genes in R_NPC H2O2 aging model. **B**, **C** Proteins that can bind to E2F3 mRNA were analyzed using CLIP-seq data from the POSTAR3 database. **D**, **E** The mRNA and protein expression levels of YTHDF2 after the addition of 10 ng/ml IL-1β to H_NPC were detected by qPCR and Western blot. **F**, **G** qPCR and immunofluorescence were used to detect mRNA and protein expression levels of YTHDF2 in the H_NPC replicative senescence model. **H** Immunofluorescence detection of Ythdf2 protein expression levels in NP tissue of naturally aging mice. **I** Immunohistochemical detection of YTHDF2 protein expression levels in human degenerating NP tissue. **J**, **K** Western blot and qPCR were used to detect the knockdown efficiency of YTHDF2 in H_NPC. **L** Flow cytometry was performed to detect the effect of knockdown of YTHDF2 on the cell cycle of H_NPC; **M** SA-β-gal staining to detect the effect of knockdown of YTHDF2 on H_NPC senescence. **N** SA-β-gal staining was used to detect the effect of knockdown of YTHDF2 on H_NPC senescence after addition of 10 ng/ml IL-1β. **O** Immunofluorescence was used to detect the effect of knockdown of YTHDF2 on P16 protein after addition of 10 ng/ml IL-1β. **P** Signal distribution map of YTHDF2 binding to the E2F3 gene: *x*-axis is the position of the gene, *y*-axis is the signal mean RPKM. **Q** Metagene plot: the gene is divided into three segments, 5’UTR, CDS and 3’UTR, and the distribution of peaks in each segment is counted. **R** The E2f3 peak from the RIP-seq data is plotted. **S** Western blot to detect the protein expression level of E2F3 after knockdown of YTHDF2 at H_NPC. **T** qPCR was performed to detect the mRNA expression level of E2F3 after knockdown of YTHDF2 at H_NPC. **U** qPCR detection of the degradation rate of E2f3 mRNA after knockdown of Ythdf2. **V** E2F3 expression was detected using the YTHDF2 KO database GSE37258. **W**, **X** Experimental scheme for RNA pulldown assay; Biotin-labelled single-stranded RNA ss-m6A and biotin-labelled ss-A were used. The western blot shows that the Ythdf2 protein has been pulled down. Data presented as mean ± SD. One-way ANOVA was used for comparison among multiple groups. *P < 0.05; **P < 0.01; ***P < 0.001; ****P < 0.0001.

We found that the m6A reader gene Ythdf2 is involved in the R_NPC senescence process (Fig. 7A). After analyzing CLIP-seq data from the POSTAR3 database, we determined that YTHDF2 and METTL3 could co-bind to the E2F3 mRNA (Fig. 7B, C). YTHDF2 mRNA and protein expression levels were significantly upregulated in both the IL-1β-induced IVDD model and replicative aging model (Fig. 7D–G). Additionally, YTHDF2 protein expression levels were significantly upregulated in naturally aged mouse NP tissues and highly degenerated human NP tissues (Fig. 7H, I). Western blot and qPCR data confirmed successful knockdown of YTHDF2 (Fig. 7J, K). Flow cytometry results showed that YTHDF2 knockdown significantly promoted the G1/S phase transition of the cell cycle (Fig. 7L), with further experiments indicating that it also significantly delayed NPC senescence and reduced P16 expression levels (Fig. 7M–O). A signal distribution map of RIP-Seq data (GSE142827) helped us determine that YTHDF2 can bind to the E2F3 gene (Fig. 7P–R). Western blot and qPCR results showed that knocking down YTHDF2 significantly increased the E2F3 mRNA and protein expression levels (Fig. 7S, T). In addition, E2f3 mRNA stability was significantly improved following Ythdf2 knockdown in the R_NPC model (Fig. 7U). Bioinformatics analysis also indicated that YTHDF2 KO significantly promoted E2F3 expression (Fig. 7V). Methylated single-stranded RNA baits (ss-m6A oligo, consensus sequences AG(m6A) CT and GG(m6A) CC) were used in an RNA pulldown assay, with unmethylated control RNA (ss-A) used as control. Interestingly, in the cell nuclear lysates, Ythdf2 was pulled down by ss-M6A oligomers, but not by ss-A oligomers (Fig. 7W, X). This suggests that, in the presence of m6A labeling, Ythdf2 can bind to E2f3 mRNA.

### ELF1 promotes NPC senescence through transcriptional regulation of YTHDF2

**Fig. 8:**
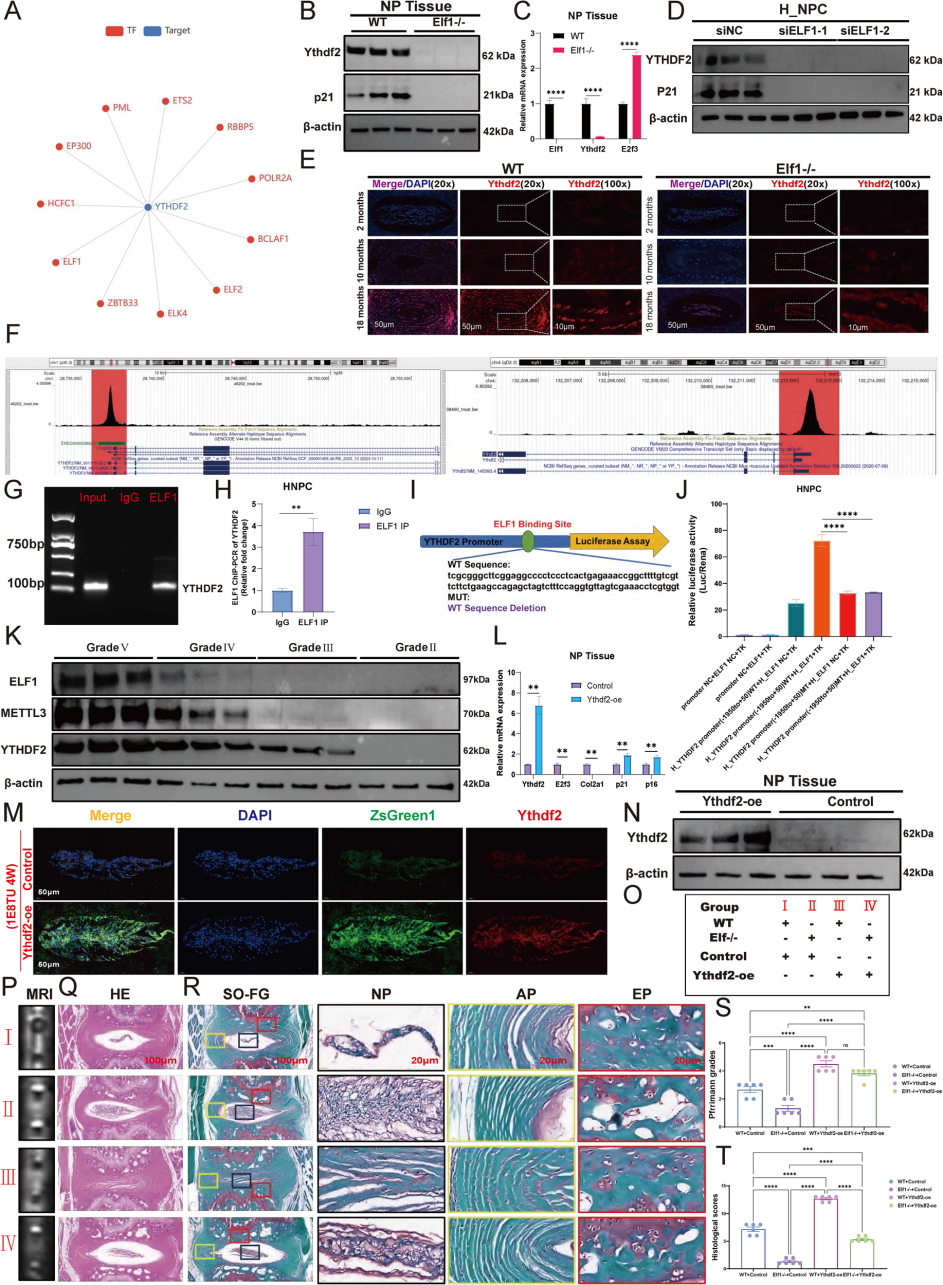
ELF1-mediated transcriptional regulation of YTHDF2 promotes NPC senescence. **A** The transcription factor database (http://www.grndb.com/) for single-cell sequencing was used to predict the potential transcription factors regulating YTHDF2 expression. **B**, **C** Western blot and qPCR analyses were performed to detect the Ythdf2 protein and mRNA expression levels in NP tissues after Elf1 knockout (KO). **D** The YTHDF2 protein expression levels were detected using western blot analysis following ELF1 knockdown in H_NPC. **E** The Ythdf2 protein expression patterns were detected in NP tissues of Elf1 KO mice using immunofluorescence. **F** An ELF1 binding peak was detected in the YTHDF2 promoter region using the open ChIP-seq database. **G** The YTHDF2 promoter sequence was detected by PCR analysis of ELF1 immunoprecipitates. **H** ELF1 binding to the YTHDF2 promoter in H_NPC was confirmed by ChIP-PCR. **I** ELF1-like elements in the promoter region of the human YTHDF2 gene. **J** Luciferase activity, driven by the YTHDF2 promoter, was more pronounced following ELF1 overexpression. In contrast, in the absence of ELF1, the luciferase activity was decreased; The luciferase activity was unchanged with the mutant YTHDF2 reporter following ELF1 overexpression. **K** The protein expression levels of ELF1, METTL3, and YTHDF2 were detected in human NP tissues using western blot analysis. **L**, **M** The Ythdf2 mRNA and protein expression levels were examined using qPCR and immunofluorescence following injection of the Ythdf2-overexpressing lentivirus into mouse NP tissues. **N** Western blot analysis was used to examine the Ythdf2 protein expression levels in mouse NP tissues. **O** Grouping of the Ythdf2-overexpressing lentiviruses injected into the NP tissues of Elf KO mice. **P**, **S** MRI was used to detect the T2-weighted signal intensity of the intervertebral discs after injection of the Ythdf2-overexpressing lentivirus into the NP tissues of wild-type (WT) and Elf1 KO mice; *N* = 6. **Q**, **R**, **T** H&E and Safranin-O staining of intervertebral discs after injection of the Ythdf2-overexpressing lentivirus into the NP tissues of WT and Elf1 KO mice; *N* = 6; Scale bars = 100 μm and 20 μm. The data are presented as the mean ± SD. One-way ANOVA was used for comparisons among multiple groups. *P < 0.05; **P < 0.01; ***P < 0.001; ****P < 0.0001.

Our transcription factor database of single-cell sequencing data identified ELF1 as a potential transcription factor that can also regulate YTHDF2 expression (Fig. 8A). Western blot and qPCR results showed significant downregulation of Ythdf2 protein and mRNA expression levels in NP tissues following Elf1 KO (Fig. 8B, C). YTHDF2 expression levels were significantly reduced by ELF1 knockdown in H_NPC (Fig. 8D). Immunofluorescence results confirmed that Ythdf2 protein expression was significantly lower in the NP tissues of Elf1 KO mice (Fig. 8E). Public ChIP-seq data further identified the YTHDF2 gene promoter region as having abundant ELF1 protein binding peaks (Fig. 8F). We then identified the ELF1 binding site in the YTHDF2 promoter and observed clear YTHDF2 DNA amplification after immunoprecipitation with an anti-ELF1 antibody (Fig. 8G). ChIP-qPCR further demonstrated that ELF1 can bind to the YTHDF2 promoter fragment (Fig. 8H). Binding site determination using luciferase reporter assays revealed that ELF1 binds to a site in the YTHDF2 promoter, with the absence of this site resulting in the inability of ELF1 to promote YTHDF2 expression (Fig. 8I, J). Additionally, YTHDF2 was synergistically upregulated with METTL3 and ELF1 in highly degenerated human NP tissues (Fig. 8K). We next constructed a lentivirus to overexpress Ythdf2 for further validation using in vivo experiments. The qPCR data showed that injecting Ythdf2-expressing lentiviruses into the discs of mice significantly promoted Ythdf2 expression, while suppressing E2f3 expression (Fig. 8L). Immunofluorescence and western blot experiments showed that intradiscal injection of the Ythdf2-overexpressing lentivirus could significantly enhance Ythdf2 protein expression in NP tissues (Fig. 8M, N). Sustained overexpression of Ythdf2 in NP tissues of 10-month-old mice significantly accelerated IVDD after two months (Fig. 8O, P, S). H&E and Safranin-O staining suggested that Elf1 KO significantly delayed the Ythdf2 overexpression-induced IVDD (Fig. 8Q, R, T). Taken together, ELF1 can accelerate NPC senescence by transcriptionally regulating YTHDF2 through binding to the YTHDF2 gene promoter region.

### The small molecule drug MMF targets and inhibits ELF1 expression to delay NPC senescence

**Fig. 9:**
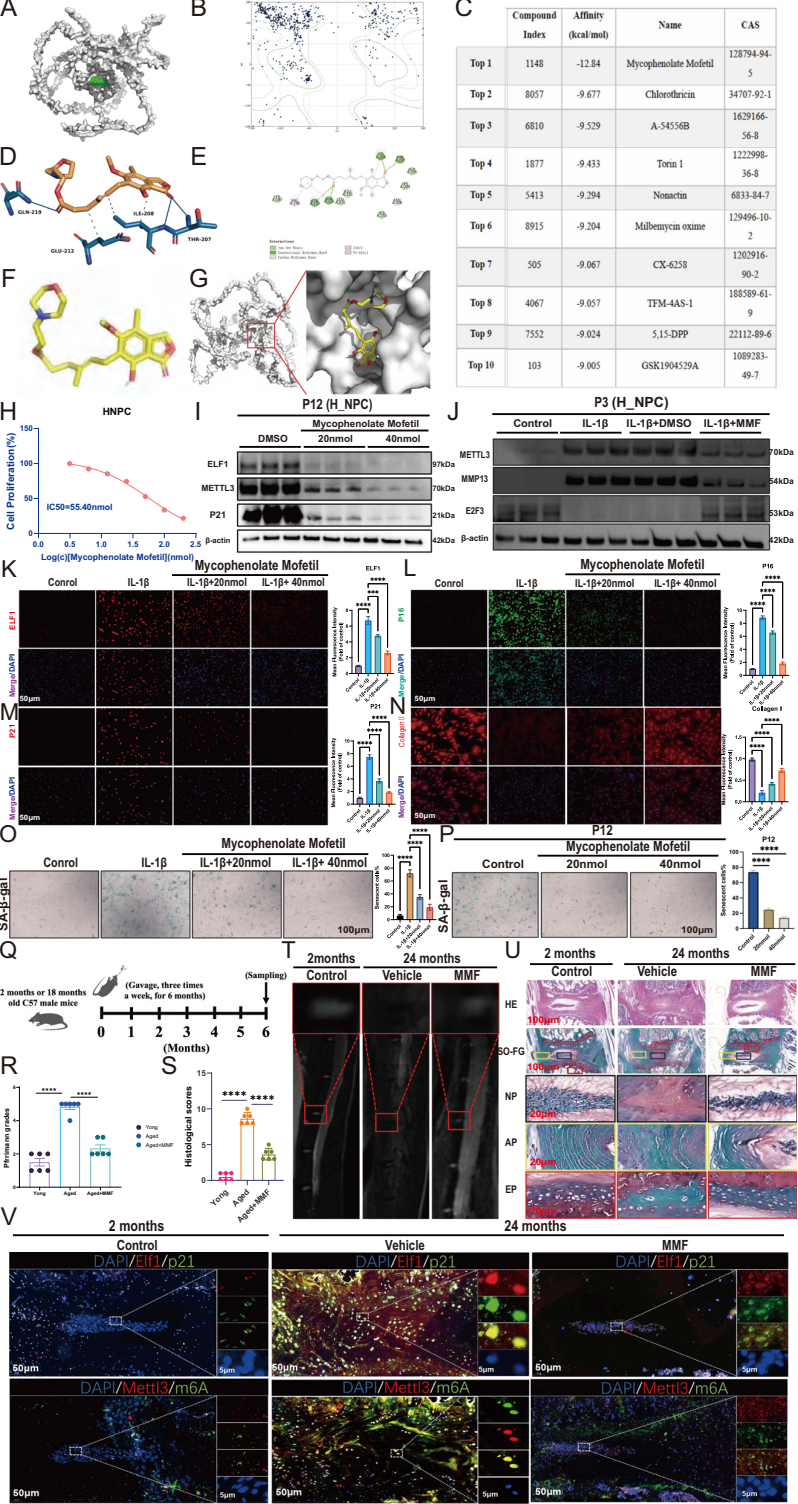
The small-molecule drug mycophenolate mofetil (MMF) targets and interferes with ELF1 expression to inhibit NPC senescence. **A** Proteins used for virtual screening of boxes (proteins are surface representations; AI predicted binding pockets are shown in green). **B** Protein Rasch plot; the number of amino acids located outside the 0.002 curve is small, accounting for 34%, suggesting that this structure can be used for subsequent calculations. **C** Docking scores for the ELF1 protein. The second to fifth columns of the table represent the Compound Index, Affinity (kcal/mol), Compound Name, and CAS number, respectively. **D** TOP1 interaction diagram of ELF1. Solid blue line: hydrogen bonding; dashed gray line: hydrophobic interaction. **E** Detailed view of the 2-dimenstional interaction of ELF1 with top 1 small molecules. **F**, **G** Top 1 small molecule structure, with the binding conformation of ELF1 to the small molecules. **H** MMF IC50 value in H_NPC. **I** Western blot analysis was used to detect the protein expression levels of ELF1, METTL3, and P21 following MMF treatment in H_NPC. **J** Western blot analysis was used to detect the protein expression levels of METTL3, MMP13, and E2F3 following MMF treatment in the 10 ng/mL IL-1β model. **K**–**N** Immunofluorescence was used to detect the protein expression patterns of ELF1, P21, P16, and Collagen II after the addition of MMF with 48 h of 10 ng/mL IL-1β treatment. **O** SA-β-gal staining was used to detect the number of senescent NP cells after the addition of MMF with 48 h of 10 ng/mL IL-1β treatment. **P** SA-β-gal staining was used to detect the amount of H_NPC senescence after the addition of MMF in the replicative senescence model. **Q** Flowchart of the experiment. Juvenile (2 months old, males, *N* = 6) or naturally aged (18 months old, males, *N* = 12) C57BL/6 J mice were given a carrier (carboxymethylcellulose sodium (CMC-Na) or MMF (30 mg/kg/day)) orally. The lumbar vertebrae were collected for histological examination after 6 months of continuous experiments with MRI followed by execution. **R**, **T** MRI detection of the T2-weighted signal intensity of the intervertebral discs in MMF-treated mice; *N* = 6. **S**, **U** H&E and Safranin-O staining of mouse intervertebral discs after MMF treatment; *N* = 6; Scale bars = 100 μm and 20 μm. **V** Immunofluorescence was used to examine the protein expression patterns of Elf1, p21, and Mettl3, as well as the m6A modifications, in the NP tissues of MMF-treated mice. The data are presented as the mean ± SD. One-way ANOVA was used for comparisons among multiple groups. *P < 0.05; **P < 0.01; ***P < 0.001; ****P < 0.0001.

We further investigated small molecule drugs or compounds that can target ELF1 binding and inhibit NPC senescence. Firstly, AlphaFold2 software was used to predict the ELF1 structure, then assess its reliability (Fig. 9A, B). Docking prediction analyses showed that the compound MMF (binding energy = -12.84 kJ/mol) could effectively bind to ELF1 and was ranked as the first compound (Fig. 9C). We obtained detailed maps of the interaction between the ELF1 protein and MMF (Fig. 9D, E) and structural conformation of MMF (Fig. 9F, G). MMF has an IC50 value of 55.40 nmol in H_NPC (Fig. 9H). Western blot results confirmed that the ELF1, METTL3, and P21protein expression levels were significantly inhibited with increasing MMF concentrations (Fig. 9I). In addition, MMF significantly inhibited the IL-1β-induced upregulation of METTL3 (Fig. 9J). Immunofluorescence results showed that MMF treatment also reversed the IL-1β-induced increase in P16 and P21 expression levels and decrease in collagen II expression levels (Fig. 9K–N). Furthermore, MMF significantly reduced the number of senescent NPCs in the IL-1β senescence model, as well as in the replicative senescence model (Fig. 9O, P). MMF (30 mg/kg/day) or control was given by gavage three times a week for 6 months to naturally senescent 18-month-old mice (Fig. 9Q). A higher T2-weighted signal intensity was seen in the IVDs of mice treated with continuous oral MMF compared with naturally aged discs, according to MRI and Pfirrmann grading analysis (Fig. 9R, T). H&E and Safranin-O staining showed that MMF treatment significantly increased the amount of NP tissues with decreased histological scores compared with the naturally aging group (Fig. 9S, U). IHC staining also demonstrated that MMF treatment significantly reduced the Elf1, p21, and Mettl3 protein expression levels, as well as the overall abundance of m6A modifications, in senescent NP tissues (Fig. 9V). We further developed a model of D-galactose (125 mg/kg/day)-induced NP tissue senescence with and without MMF supplementation (Supplementary Fig. 9A). MRI and Pfirrmann grading results indicated that the D-galactose-induced discs in mice had a significantly reduced T2-weighted signal intensity, while the discs in MMF-administered mice had an increased T2-weighted signal intensity (Supplementary Fig. 9B–D). H&E and Safranin-O staining showed that MMF treatment could significantly increase the number of NP tissues and delay IVDD (Supplementary Fig. 9E, F). IHC staining demonstrated that MMF treatment significantly reduced the Mettl3, p21, and p16 protein expression levels, as well as the overall abundance of m6A modifications, in senescent NP tissues (Supplementary Fig. 9G). Next, we constructed a rat acupuncture IVDD model to further validate the role of MMF. The MRI and Pfirrmann grading results showed that injections of MMF had the same effects as injections of an Elf1 small interfering RNA (siRNA) in the needle model of IVDD, both of which increased the T2-weighted signal intensity of the discs (Supplementary Fig. 10A–E). H&E and Safranin-O staining demonstrated that both the MMF and Elf1 siRNA treatments significantly delayed IVDD (Supplementary Fig. 10F–K). IHC staining confirmed that the MMF and Elf1 siRNA treatments could revert the expression of E2f3 and Collagen II in IVDD (Supplementary Fig. 10L, M). In summary, these data suggest that MMF can target Elf1 and suppress its expression, thereby delaying NPC senescence and attenuating IVDD.

### Overexpressing Elf1 in vivo accelerates NPC senescence to promote IVDD

**Fig. 10:**
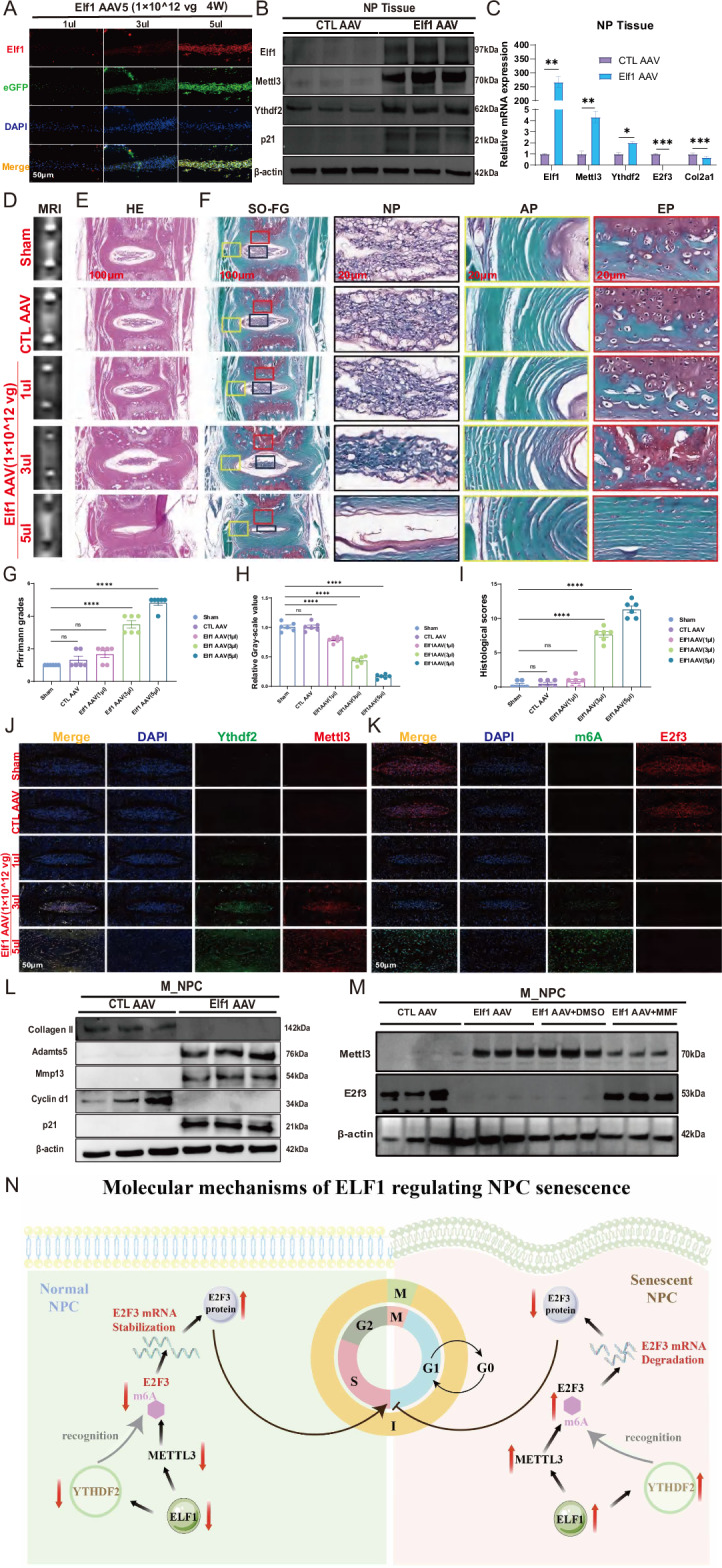
Overexpressing Elf1 in vivo accelerates NPC senescence to promote IVDD. **A** Immunofluorescence detection of Elf1 protein expression patterns in NP tissues after injection of different doses of Elf1 AAV5. **B**, **C** Western blot and qPCR analyses were used to detect the protein and mRNA expression levels of Elf1, Mettl3, and Ythdf2 in NP tissues after Elf1 overexpression. **D**, **G**, **H** MRI detection of the T2-weighted signal intensity and Pfirrmann grading of intervertebral discs in mice after injection of Elf1 AAV5; *N* = 6. **E**, **F**, **I** H&E and Safranin-O staining and histological scoring of mouse intervertebral discs after injection of Elf1 AAV5; *N* = 6; Scale bars = 100 μm and 20 μm. **J**, **K** Immunofluorescence detection of the protein expression patterns of Elf1, p21, and Mettl3 and the m6A abundance in the NP tissues of mice treated with an injection of Elf1 AAV5. **L** Western blot analysis was used to detect the protein expression levels of Collagen II, Adamts5, Mmp13, Cyclin D1, and p21 in M_NPC after Elf1 overexpression. **M** Western blot analysis was used to detect the protein expression levels of Mettl3 and E2f3 after MMF treatment in Elf1-overexpressing M_NPC. **N** Molecular mechanisms of ELF1-mediated regulation of NPC senescence. The data are presented as the mean ± SD. One-way ANOVA was used for comparisons among multiple groups. *P < 0.05; **P < 0.01; ***P < 0.001; ****P < 0.0001.

To further validate the role of Elf1 in IVDD, we constructed an adeno-associated virus (AAV5) overexpressing Elf1. Immunofluorescence results showed a gradual increase in Elf1 protein expression levels with higher Elf1 AAV5 viral loads (Fig. 10A). Western blot and qPCR results suggested that overexpressing Elf1 in mouse NP tissues could significantly increase the Mettl3 and Ythdf2 expression levels (Fig. 10B, C). MRI and Pfirrmann grading analyses showed a lower T2-weighted signal intensity in mouse IVDs after continuous addition of Elf1 AAV5 (Fig. 10D, G, H). H&E and Safranin-O staining confirmed that overexpressing Elf1 significantly reduced the amount of NP tissues and increased the histological scores (Fig. 10E, F, I), with IHC staining showing that the Mettl3 and Ythdf2 protein expression levels became gradually enhanced with higher expression of Elf1 in the NP tissues (Fig. 10J). Moreover, overexpressing Elf1 significantly increased the m6A abundance in NP tissues and inhibited E2f3 protein expression (Fig. 10K). In addition, western blot analysis showed that overexpression of Elf1 in M_NPC promoted the protein expression levels of Adamts5, Mmp13, and p21, while decreasing those of collagen II and Cyclin d1 (Fig. 10L). Elf1 overexpression also promoted Mettl3 expression and inhibited E2f3 expression, but these patterns were reversed with MMF treatment (Fig. 10M). The immunofluorescence data confirmed that the addition of MMF significantly reduced the elevation of Mettl3, p21, and p16 expression levels resulting from Elf1 overexpression (Supplementary Fig. 11A, C). Additionally, knocking down Mettl3 following Elf1 overexpression increased the expression levels of E2f3 and decreased those of senescent proteins (Supplementary Fig. 11B, D). SA-β-gal experiments confirmed that Elf1 overexpression-induced NPC senescence was delayed by treatment with MMF or a Mettl3 siRNA (Supplementary Fig. 11E–H). Flow cytometry experiments further suggested that both MMF and siMettl3 treatment could delay the G1/S phase cell cycle arrest caused by Elf1 overexpression (Supplementary Fig. 11I–L). Furthermore, qPCR results confirmed that the Mettl3 and Ythdf2 mRNA expression levels were significantly decreased in M_NPC after Elf1 KO, whereas the E2f3 mRNA expression levels were significantly increased (Supplementary Fig. 12A). Western blot analysis indicated that the Elf1 KO-induced increase in E3f3 protein expression was significantly reversed by overexpression of Mettl3 and Ythdf2 (Supplementary Fig. 12B, C). SA-β-gal work showed that Elf1 KO can slow the senescence caused by high expression levels of Mettl3 and Ythdf2 (Supplementary Fig. 12D–G). Overexpressing Mettl3 and Ythdf2 in M_NPC could promote the G1/S phase arrest, whereas Elf1 KO accelerated the G1/S phase cell cycle transition (Supplementary Fig. 12H–K). Immunofluorescence confirmed that MMF inhibited the high expression patterns of the senescent proteins p21 and p16 caused by Ythdf2 overexpression (Supplementary Fig. 12L–O). Taken together, MMF treatment can delay NPC senescence by targeted inhibition of the Elf1-Mettl3/Ythdf2-E2f3 axis.

## Discussion

Recently, senescent NPCs accumulation caused by aging and other injurious factors has been identified as a significant marker for the onset and progression of IVDD [9, 33, 34]. Elucidating the underlying mechanisms of NPC aging to assist with developing new therapeutic strategies has become a current research hotspot and critical issue to be addressed. Therefore, the current diagnostic and therapeutic methods of IVDD should be shifted to precision therapy using specific molecular biological mechanisms, aiming to inhibit NPC senescence from the root cause to thereby slow IVDD progression.

The modern scientific study of aging focuses on answering several interrelated questions: Why do organisms age? How do cells become senescent? How, and to what extent, can the aging process be slowed down, stopped, or even reversed? Therefore, it is important to have a better understanding of what initiates NPC senescence. Several recent studies have shown that perturbations in transcriptional networks and chromatin states involved in the control of cell fate, metabolism, and stress response pathways are an early driver and one of the possible underlying causes of senescence [35, 36]. Transcription factor-driven epigenomic changes are critical for aging. The genetic regulation of a single transcription factor, which is conserved throughout evolution, may be sufficient to affect the health and lifespan of an organism [14, 15, 37]. During disc development, a number of transcription factors, such as Mkx, Pax1/9, Shh, Foxa1/2, T-Brachyury, and Sox5/6/9, orchestrate the differentiation of the NP and AF [38]. Therefore, identifying the specific transcription factors that regulate IVD development is key to unravelling the gene expression networks that can serve as targets or tools for regenerative medicine strategies for IVD-related diseases. However, the transcription factors that drive NPC senescence and promote IVDD development are poorly understood. In this study, using a multi-omics approach, we found for the first time that the transcription factor ELF1 drives NPC senescence and accelerates IVDD pathogenesis. ELF1 expression levels progressively increased with higher Pfirrmann grading in human degenerating NP tissues. ELF1 expression patterns were also significantly higher in our rat acupuncture IVDD model, as well as in naturally aged degenerating NP tissues. Additionally, we used an Elf1 KO animal model and NPC senescence model to confirm that reducing these ELF1 expression levels could significantly delay NPC senescence and inhibit IVDD. In addition, we constructed an Elf1 AAV5 to overexpress Elf1 in mouse IVDs, which significantly accelerated the NP tissue senescence and IVDD onset. Our findings demonstrate that the degenerative changes in both nucleus pulposus (NP) and annulus fibrosus (AF) tissues exhibit progressive deterioration with aging in mice. Notably, we observed a substantial upregulation of Elf1 expression in aged NP and AF tissues, indicating that Elf1 not only plays a critical role within the NP but may also be actively involved in the pathogenesis of AF degeneration. Importantly, our comparative analysis revealed that Elf1 knockout (KO) significantly attenuates the degenerative progression of AF tissue when compared to aged mouse disc tissues, suggesting a potential therapeutic target for intervertebral disc degeneration.

Our study further explored the specific molecular mechanisms by which high ELF1 expression can promote NPC senescence. We analyzed a single-cell database to identify differentially expressed genes enriched in cellular senescence and m6A-containing RNA binding in the ELF1^+^ and ELF1^-^ subpopulations of human NP tissues. Recent studies have demonstrated that epigenetic regulatory mechanisms, including DNA methylation, histone modification, non-coding RNAs, and RNA methylation, are closely associated with the pathogenesis of NPC aging and age-related IVDD [39, 40]. One of the most common RNA modifications in mammals is m6A, which involves the interplay of m6A methyltransferases (“writers”), m6A demethylases (“erasers”), and reader proteins, to control RNA nuclear transport, splicing, stability, translation, and metabolism [18, 20]. Numerous reports have implicated m6A modifications in oxidative stress, DNA damage, cellular senescence, autophagy, neurodegenerative disease, diabetes, cardiovascular disease, and other age-related processes [21, 41]. Recently, several studies have investigated the biological functions of m6A modifications in NPC ageing and IVDD, and abundant m6A modifications were found in TNF-α-induced NPC senescence, a puncture-induced IDD animal model and a natural NP senescence model [42]. The study by Chen et al. has revealed the dynamic changes of N6-methyladenosine (m6A) modification in nucleus pulposus (NP) tissues during natural aging and its potential role in intervertebral disc degeneration (IVDD). MeRIP-Seq was utilized to analyze the m6A transcriptome in NP tissues at three different time points (2, 10, and 20 months). In the 2-month (2 M) group, 31,986 m6A peaks were identified in 15,166 genes; in the 10-month (10 M) group, 32,007 peaks in 15,079 genes; and in the 20-month (20 M) group, 19,322 peaks in 13,448 genes. More interestingly, it was found that a total of 931 genes showed a trend towards persistent m6A methylation during the senescence of the NPC [23, 43]. Moreover, in a study by Cao et al., a mouse IVDD standing model was constructed, and MeRIP-seq analysis of NP tissues revealed 8,173 genes in the experimental group and 8,796 genes in the control group. There were 7,321 overlapping genes between the two groups, with 852 unique genes in the experimental group and 1,475 unique genes in the control group. Additionally, 17,846 distinct peaks were detected in the 8,173 genes of the experimental group, while 19,742 distinct peaks were identified in the 8,796 genes of the control group. The study also found 933 significantly upregulated m6A peaks and 386 significantly downregulated m6A peaks [22]. This suggests that persistent changes in m6A peaks may play a key role in the development of IVDD, and that genes with persistent methylation trends are primarily associated with RNA biosynthesis and cellular senescence. Therefore, a deeper understanding of the drivers of m6A dynamics during NPC aging will help identify intrinsic causative factors and further provide new therapeutic targets for IVDD. In this study, our multi-omics analysis revealed significantly upregulated expression levels of the methylase METTL3 in degenerating human NP tissues, as well as in senescent NPCs, which is consistent with the results of studies that used a standing mouse model and natural aging model [22, 23]. Here, our ChIP-seq data using an anti-ELF1 antibody in human NPCs revealed that the peak neighboring genes were enriched in aging. ChIP-qPCR and dual luciferase experiments in H_NPC confirmed that ELF1 can directly bind to the METTL3 promoter to transcriptionally regulate its expression, which was confirmed when significantly reduced METTL3 mRNA and protein expression levels were observed following ELF1 knockdown. Significantly lower Mettl3 expression levels and m6A modifications were found in the Elf1 KO mice IVDs. Knocking down METLL3 could significantly delay NPC senescence. In addition, overexpressing Mettl3 in mouse IVDs significantly accelerated NPC aging, reversing the delay in this process from Elf1 KO. In summary, ELF1 can accelerate IVDD by increasing the abundance of m6A modifications through the transcriptionally activation of METLL3 to promote NPC senescence. To our knowledge, this is the first study to demonstrate that ELF1 can drive more abundant m6A modifications in NPC senescence.

A common feature of senescent cells is intrinsic, irreversible cell cycle arrest, which may be an alarm response to noxious stimuli or abnormal proliferation [44]. Cell cycle arrest limits the proliferation of damaged cells, with E2F family proteins being key downstream transcription factors that regulate the cell cycle, as well as DNA repair, apoptosis, and senescence. Overactivation of the E2F family is associated with increased the oxidative stress, imbalanced cellular metabolism, and reduced cellular function that are hallmarks of the aging process. Furthermore, regulating E2F family transcription factor activity can increase the ability of cells to counteract DNA damage, suggesting that aging may be delayed by regulating these proteins [45–47]. In the present study, we found that E2F family transcription factors were downregulated in senescent NPCs, with E2f3 expression levels significantly increasing after Mettl3 knockdown. This suggests that E2f3 is involved in NPC senescence as a downstream methylation target of Mettl3. The E2F3 methylation levels in human embryonic lung fibroblasts were shown to be higher in prematurely aged cells compared with young cells [48], suggesting that E2F3 mRNA m6A modifications may be an important factor in cellular senescence. Additionally, other work has demonstrated a significant decrease in E2F3 expression levels during NPC senescence [24], but the involvement of m6A modifications in these expression patterns has not been reported. In this study, we found for the first time that high expression levels of the methylase METTL3 could increase the abundance of E2F3 mRNA m6A modifications, leading to decreased E2F3 mRNA and protein expression levels that thereby accelerate NPC senescence.

Although m6A writers and erasers affect the RNA methylation patterns, m6A binding proteins ultimately determine the biological outcomes of these modifications [49, 50]. In this study, we found that the mRNA degradation rate increased significantly with a higher abundance of m6A-modified E2F3 mRNA in NPC. Therefore, it is necessary to search for m6A readers that can recognize the m6A site of E2F3 mRNA and accelerate its degradation. The YTH structural domain family protein 1 (YTHDF1) has been shown to initiate RNA translation by interacting with translation initiation factors and ribosomes, whereas YTHDF2 selectively binds m6A-modified transcripts and accelerates their degradation [51, 52]. The (TG)_n_ microsatellite polymorphism in the fourth intron of the YTHDF2 gene is associated with human longevity [53]. In the present study, we found that YTHDF2 expression levels were significantly increased in senescent NPCs and highly degenerated human NP tissues, with YTHDF2 knockdown resulting in higher E2F3 mRNA stability. In addition, ChIP-qPCR and dual luciferase experiments in H_NPC confirmed that ELF1 can directly bind to the YTHDF2 promoter to transcriptionally regulate its expression, which was further confirmed when ELF1 knockdown led to significantly reduced YTHDF2 mRNA and protein expression levels. Elf1 KO mice IVDs displayed significantly reduced Ythdf2 expression levels and m6A modifications. Overexpressing Ythdf2 in mouse IVDs significantly accelerated NPC senescence, reversing the delay in this process that occurred from Elf1 KO. Thus, our study shows for the first time that increased ELF1 expression patterns in NPC can simultaneously promote the expression levels of the methylase METTL3 and reading protein YTHDF2, thereby affecting the m6A modification abundance in NPCs to drive their senescence.

Conservative and surgical strategies are the current treatment modalities for IVDD. Unfortunately, a significant number of patients do not respond to conventional therapies and continue to suffer from chronic pain and disability [54]. Recently, small molecules, defined as low molecular weight organic compounds less than 900 daltons, have been shown to have anti-inflammatory, anti-apoptotic, antioxidant, and anabolic properties. They may therefore potentially help prevent further disc degeneration and promote disc cell regeneration [55], leading to small molecule therapy becoming a traditional treatment for discogenic pain and an alternative to surgery. We aimed to identify small molecule compounds that can directly target and inhibit ELF1 for the early prevention and mitigation of IVDD. We predicted the structure of ELF1 using AlphaFold2 software and found that MMF displayed the highest potential for binding to ELF1. MMF is prepared as mycophenolic acid (MPA) 2-morpholinoethylester, with oral MMF having an availability of 94.1%. After absorption, esterases in the plasma, liver, and kidneys rapidly convert MMF to the active metabolite MPA [56]. Studies have shown that MPA can prolong the replicative lifespan of yeast [57, 58]. MMF has also been defined as an ‘anti-aging’ drug, as it has been shown to ameliorate oxidative stress, as well as inhibit macrophage and lymphocyte infiltration and cytokine production by these cells [59–61]. In addition, after acute spinal cord injury in young rats, MMF displayed anti-apoptotic, anti-lipid peroxidation, and neuroprotective effects [62]. In the present study, we have demonstrated for the first time that MMF can significantly inhibit ELF1 expression and reduce the m6A modification abundance, thereby delaying NPC senescence and attenuating IVDD progression. These findings suggest that MMF may be an effective therapeutic strategy for IVDD by inhibiting ELF1 expression and delaying NPC senescence.

## Conclusion

This study was designed from multiple perspectives, including cell biology, bioinformatics, genomics, histopathology, and gene knockout animal models. It proposed the mechanisms and targets for inhibiting nucleus pulposus (NP) cell senescence and elucidated the molecular mechanism by which the ELF1-METTL3/YTHDF2-m6A-E2F3 signaling axis promotes NP cell senescence. Additionally, using artificial intelligence-based virtual screening technology, the small-molecule active compound mycophenolate mofetil (MMF) was identified to target and modulate ELF1 expression, thereby inhibiting this signaling axis. These findings provide a solid theoretical and experimental foundation for clinically delaying intervertebral disc degeneration (IVDD), demonstrating significant translational potential in clinical applications. The limitations and shortcomings of this study mainly include the following three aspects: 1. The grade I/II intervertebral disc tissues collected in this study were primarily obtained from patients with idiopathic scoliosis and lumbar spine fractures. Due to the difficulty in acquiring normal human nucleus pulposus tissue samples, the sample size available for research is relatively limited. Considering the differences in genetic backgrounds among individuals and the varying sampling locations within the intervertebral disc (such as the junctional area between the annulus fibrosus and nucleus pulposus), there may be some degree of biological heterogeneity among the samples. 2. The number of nucleus pulposus tissue specimens subjected to proteomic sequencing in this study was relatively small, and further expansion of the sample size is necessary for validation. The RNA transcriptomics and single-cell transcriptomics analyses were primarily based on bioinformatics analysis, and there is a lack of single-cell self-measured data from IVDD nucleus pulposus tissues in our department. Further multidimensional validation should be conducted by combining the expression levels of ELF1 in nucleus pulposus tissues with other clinical indicators of IVDD patients to enhance the clinical diagnostic value. 3. This study utilized ELF1 whole-gene knockout mice for molecular biology experiments. In future research, conditional gene knockout of ELF1 (using the Cre-loxP system) should be established, and the impact on IVDD should be observed after specifically knocking out ELF1 in the nucleus pulposus tissue.

## Materials and methods

See the Supplementary Material for more information on the materials and methods used in this study.

## Supplementary information

Supplementary Figures

Full and uncropped western blots

## Data Availability

The original contributions of this study are detailed within the article and its accompanying Supplementary Material. For any further questions, please contact the corresponding author for additional information.
