# Correction: ELF1-mediated transactivation of METTL3/YTHDF2 promotes nucleus pulposus cell senescence via m6A-dependent destabilization of E2F3 mRNA in intervertebral disc degeneration

**DOI:** 10.1038/s41420-026-03153-4

**Published:** 2026-05-29

**Authors:** Xiao-Wei Liu, Hao-Wei Xu, Shu-Bao Zhang, Yu-Yang Yi, Sheng-Jie Chang, Shan-Jin Wang

**Affiliations:** Department of Spinal Surgery, Shanghai East Hospital, School of Medicine, Tongji University, Shanghai, 200092 China

**Keywords:** Cell growth, Diagnostic markers

Correction to: *Cell Death Discovery* 10.1038/s41420-025-02515-8, published online 04 June 2025

In the original version of this article, an error occurred during figure assembly. Due to a copy-paste mistake, the Ythdf2 immunofluorescence image in Figure 8E was inadvertently misplaced during image assembly and upload, and the correct original image was not presented. The accurate Figure 8E has now been replaced, as shown in the Amended Figure 8. Furthermore, in Figure 8K, the molecular weight of YTHDF2 should be 62 kDa instead of 70 kDa, which we have modified in the Amended Figure 8. This correction does not affect the results or conclusions of the study. We apologize for any confusion or inconvenience caused.

**Original Figure 8** 
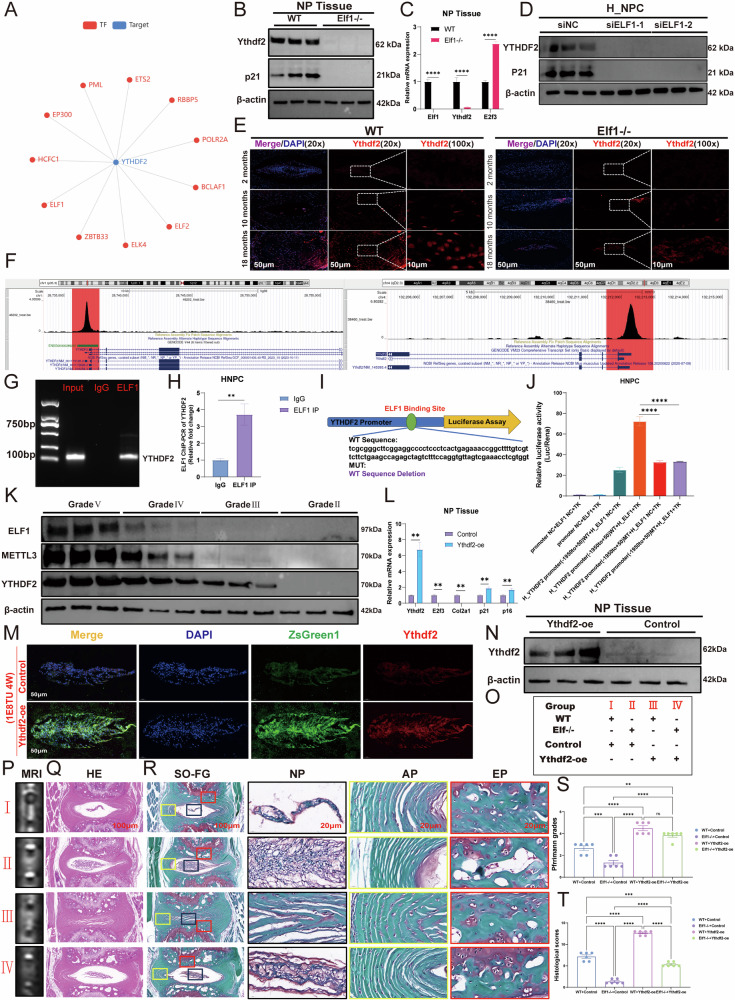


**Amended Figure 8** 
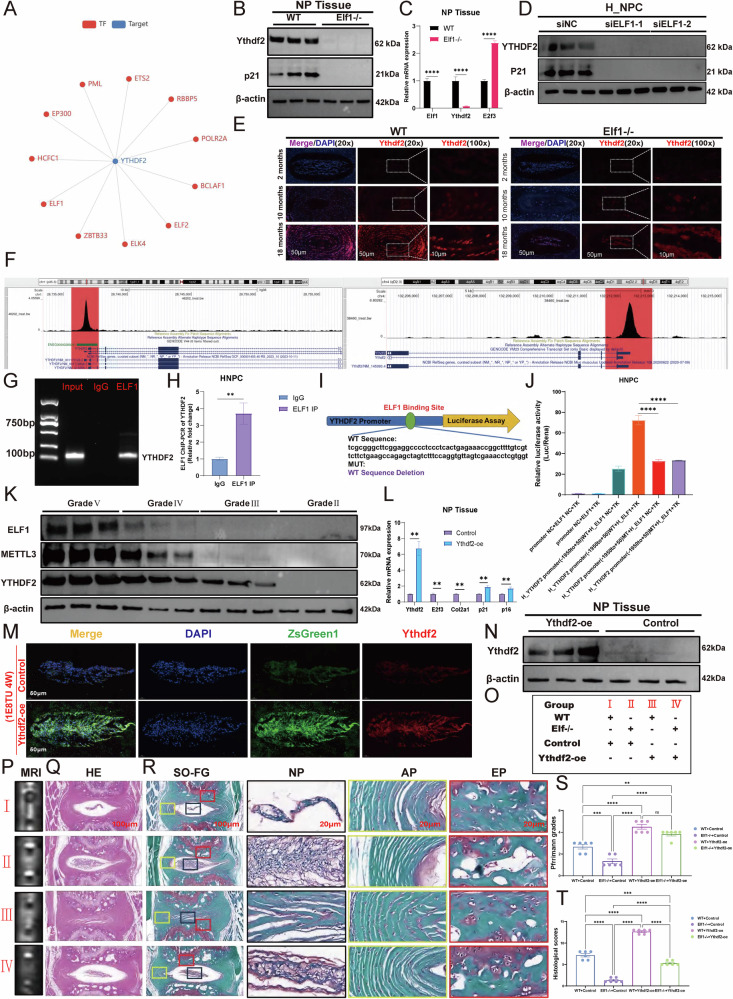


The original article has been corrected.

