# Supplementary material for: ELF1-mediated transactivation of METTL3/YTHDF2 promotes nucleus pulposus cell senescence via m6A-dependent destabilization of E2F3 mRNA in intervertebral disc degeneration
Source: Cell Death Discov. 2025 Jun 4;11:267. doi: 10.1038/s41420-025-02515-8 (PMC12137937; doi:10.1038/s41420-025-02515-8)
Supplement: Supplementary file 1 — Supplementary Figures [file 41420_2025_2515_MOESM1_ESM.pdf]

Supplementary Materials

ELF1-mediated transactivation of METTL3/YTHDF2 promotes nucleus pulposus cell senescence via m6A-dependent destabilization of E2F3 mRNA in intervertebral disc degeneration

Xiao-Wei Liu[1], Hao-Wei Xu[1], Shu-Bao Zhang[1], Yu-Yang Yi[1], Sheng-Jie Chang[1], Shan-Jin Wang*

1.Department of Spinal Surgery, Shanghai East Hospital, School of Medicine, Tongji University, Shanghai 200092, China.

Corresponding author: kingspine@163.com.

Supplementary Figures (1-12)

Supplementary Tables (1-2)

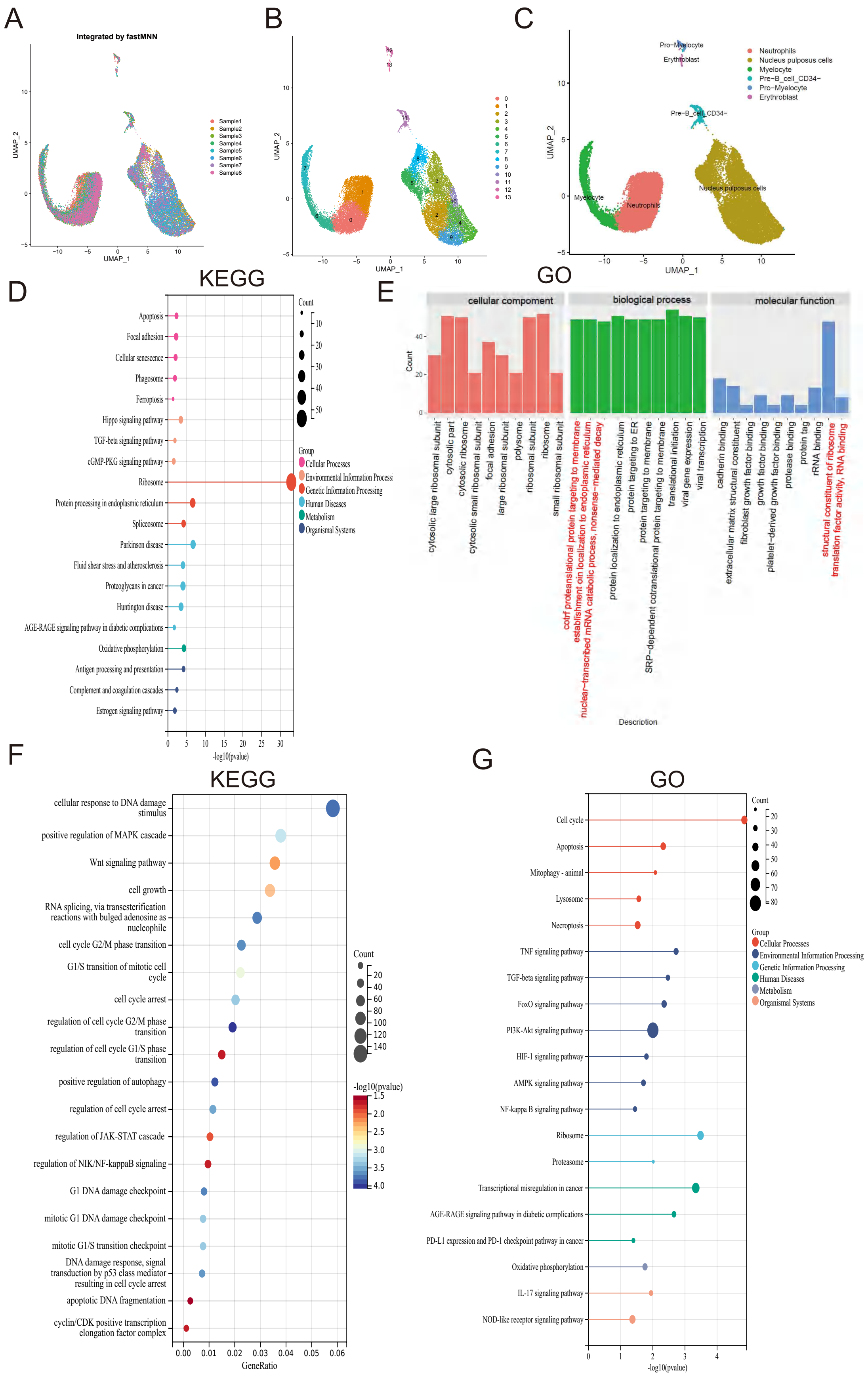
A
Integrated by fastMNN
UMAP_1
UMAP_2
Sample1
Sample2
Sample3
Sample4
Sample5
Sample6
Sample7
Sample8
B
UMAP_1
UMAP_2
0
1
2
3
4
5
6
7
8
9
10
11
12
13
C
UMAP_1
UMAP_2
Neutrophils
Nucleus pulposus cells
Myelocyte
Pre−B_cell_CD34−
Pro−Myelocyte
Erythroblast
D
KEGG
Apoptosis
Focal adhesion
Cellular senescence
Phagosome
Ferroptosis
Hippo signaling pathway
TGF-beta signaling pathway
cGMP-PKG signaling pathway
Ribosome
Protein processing in endoplasmic reticulum
Spliceosome
Parkinson disease
Fluid shear stress and atherosclerosis
Proteoglycans in cancer
Huntington disease
AGE-RAGE signaling pathway in diabetic complications
Oxidative phosphorylation
Antigen processing and presentation
Complement and coagulation cascades
Estrogen signaling pathway
-log10(pvalue)
Count
Group
Cellular Processes
Environmental Information Process
Genetic Information Processing
Human Diseases
Metabolism
Organismal Systems
E
GO
cellular compoment
biological process
molecular function
Count
cytosolic large ribosomal subunit
cytosolic part
cytosolic ribosome
cytosolic small ribosomal subunit
focal adhesion
large ribosomal subunit
polysome
ribosomal subunit
ribosome
small ribosomal subunit
cotranslational protein targeting to membrane
establishment oin localization to endoplasmic reticulum
nuclear−transcribed mRNA catabolic process, nonsense−mediated decay
protein localization to endoplasmic reticulum
protein targeting to ER
protein targeting to membrane
SRP−dependent cotranslational protein targeting to membrane
translational initiation
viral gene expression
viral transcription
cadherin binding
extracellular matrix structural constituent
fibroblast growth factor binding
growth factor binding
platelet−derived growth factor binding
protease binding
protein tag
rRNA binding
structural constituent of ribosome
translation factor activity, RNA binding
Description
F
KEGG
cellular response to DNA damage stimulus
positive regulation of MAPK cascade
Wnt signaling pathway
cell growth
RNA splicing, via transesterification reactions with bulged adenosine as nucleophile
cell cycle G2/M phase transition
G1/S transition of mitotic cell cycle
cell cycle arrest
regulation of cell cycle G2/M phase transition
regulation of cell cycle G1/S phase transition
positive regulation of autophagy
regulation of cell cycle arrest
regulation of JAK-STAT cascade
regulation of NIK/NF-kappaB signaling
G1 DNA damage checkpoint
mitotic G1 DNA damage checkpoint
mitotic G1/S transition checkpoint
DNA damage response, signal transduction by p53 class mediator resulting in cell cycle arrest
apoptotic DNA fragmentation
cyclin/CDK positive transcription elongation factor complex
GeneRatio
Count
-log10(pvalue)
G
GO
Cell cycle
Apoptosis
Mitophagy - animal
Lysosome
Necroptosis
TNF signaling pathway
TGF-beta signaling pathway
FoxO signaling pathway
PI3K-Akt signaling pathway
HIF-1 signaling pathway
AMPK signaling pathway
NF-kappa B signaling pathway
Ribosome
Proteasome
Transcriptional misregulation in cancer
AGE-RAGE signaling pathway in diabetic complications
PD-L1 expression and PD-1 checkpoint pathway in cancer
Oxidative phosphorylation
IL-17 signaling pathway
NOD-like receptor signaling pathway
-log10(pvalue)
Count
Group
Cellular Processes
Environmental Information Processing
Genetic Information Processing
Human Diseases
Metabolism
Organismal Systems

Suplementary Figure 1

(A-C) Cellular compartmentalisation of intervertebral disc single-cell sequencing data (GSE165722); (D，E) Differential gene KEGG and GO enrichment analysis in Nucleus pulposus cell (NPC) clusters from single-cell sequencing data MDD vs SDD samples; (F， G) RNA-seq data GSE34095 and GSE56081 KEGG and GO enrichment analysis of differential genes in the merged dataset.

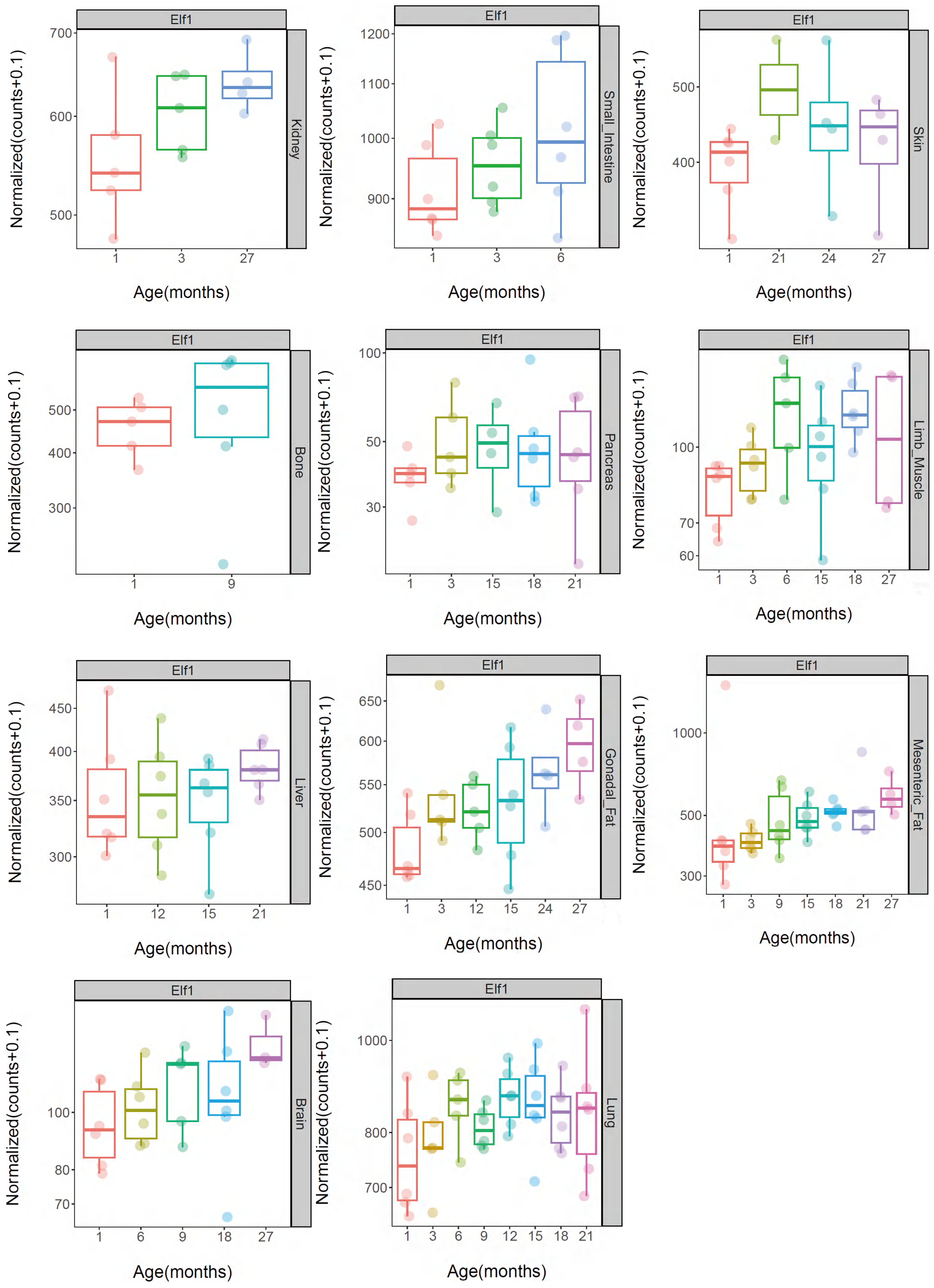
Elf1
Kidney
Small_Intestine
Skin
Bone
Pancreas
Limb_Muscle
Liver
Gonadal_Fat
Mesenteric_Fat
Brain
Lung
Normalized(counts+0.1)
Age(months)

Suplementary Figure 2

Elf1 expression in senescent bone, brain, gonadal adipose tissue, kidney, limb muscle (tibialis anterior), liver, lung, mesenteric adipose tissue (MAT), pancreas, skin and small intestine (duodenum) in multi-organ single-cell sequencing data (https://twc-stanford.shinyapps.io/maca/) from mice of different ages.

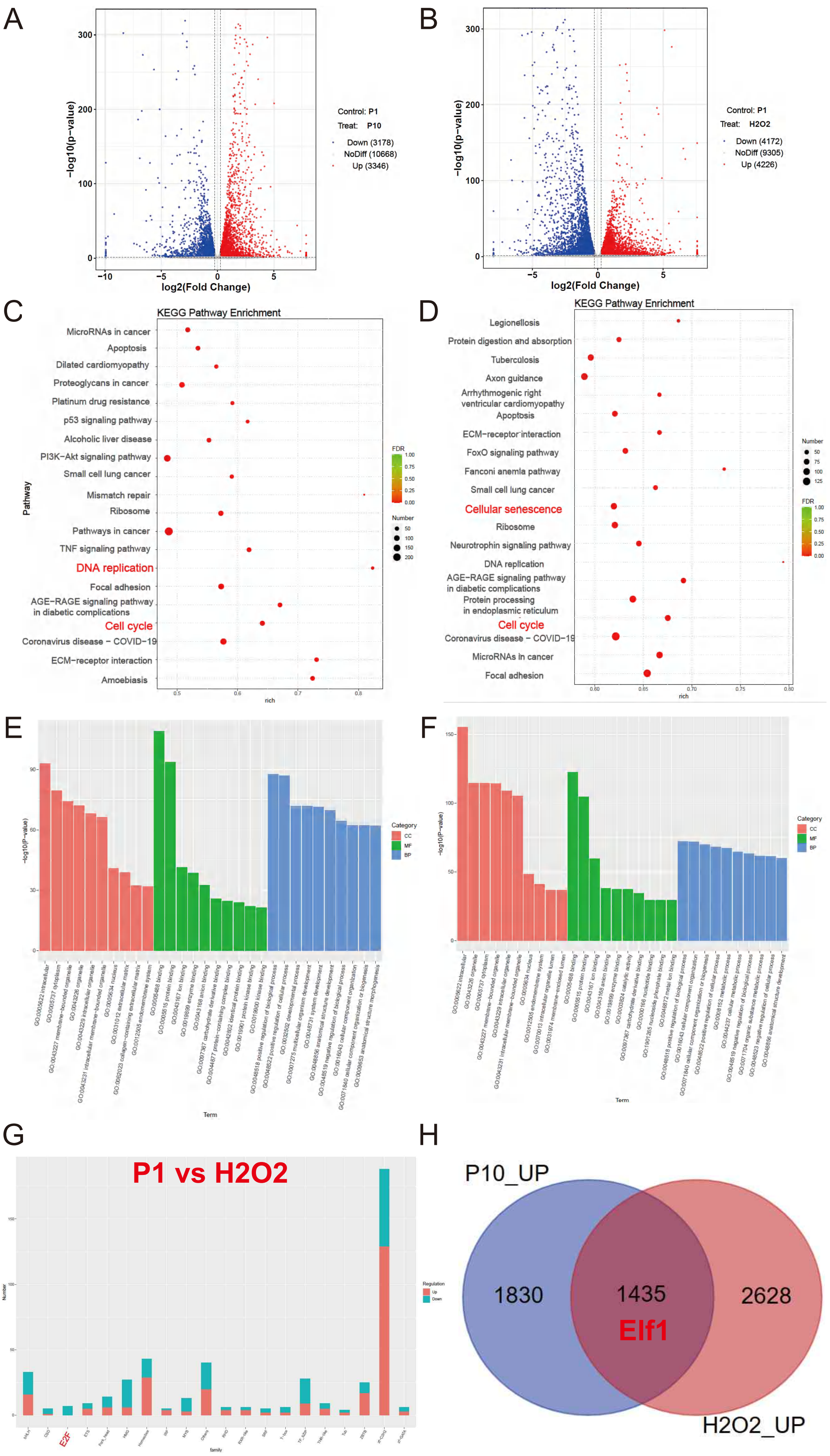
A
-log10(p-value)
log2(Fold Change)
Control: P1
Treat: P10
Down (3178)
NoDiff (10668)
Up (3346)
B
Control: P1
Treat: H2O2
Down (4172)
NoDiff (9305)
Up (4226)
C
KEGG Pathway Enrichment
Pathway
MicroRNAs in cancer
Apoptosis
Dilated cardiomyopathy
Proteoglycans in cancer
Platinum drug resistance
p53 signaling pathway
Alcoholic liver disease
PI3K-Akt signaling pathway
Small cell lung cancer
Mismatch repair
Ribosome
Pathways in cancer
TNF signaling pathway
DNA replication
Focal adhesion
AGE-RAGE signaling pathway in diabetic complications
Cell cycle
Coronavirus disease - COVID-19
ECM-receptor interaction
Amoebiasis
rich
FDR
Number
D
KEGG Pathway Enrichment
Legionellosis
Protein digestion and absorption
Tuberculosis
Axon guidance
Arrhythmogenic right ventricular cardiomyopathy
Apoptosis
ECM-receptor interaction
FoxO signaling pathway
Fanconi anemia pathway
Small cell lung cancer
Cellular senescence
Ribosome
Neurotrophin signaling pathway
DNA replication
AGE-RAGE signaling pathway in diabetic complications
Protein processing in endoplasmic reticulum
Cell cycle
Coronavirus disease - COVID-19
MicroRNAs in cancer
Focal adhesion
rich
Number
FDR
E
-log10(P-value)
Category
CC
MF
BP
Term
F
-log10(P-value)
Category
CC
MF
BP
Term
G
P1 vs H2O2
Number
Regulation
Up
Down
E2F
family
H
P10_UP
1830
1435
Elf1
2628
H2O2_UP

Suplementary Figure 3

(A) Volcano diagram showing differential genes in the P10 and P1 transcriptomes of rat nucleus pulposus cells (R_NPC) undergoing replicative senescence;（B）Volcano plot showing differential genes in H2O2 senescent R_NPC and P1 young R_NPC transcriptome sequencing;（C, D）Plot of KEGG enrichment analysis of differential genes in the R_NPC replicative senescence model and the H2O2 senescence model;（E, F）Plot of GO enrichment analysis of differential genes in the R_NPC replicative senescence model and the H2O2 senescence model;（G）A family of transcription factors differentially expressed in the H2O2-induced R_NPC senescence model; (H) Venn diagram showing the intersection of up-regulated differential genes in the R_NPC replicative aging model and up-regulated differential genes in the H2O2 aging model.

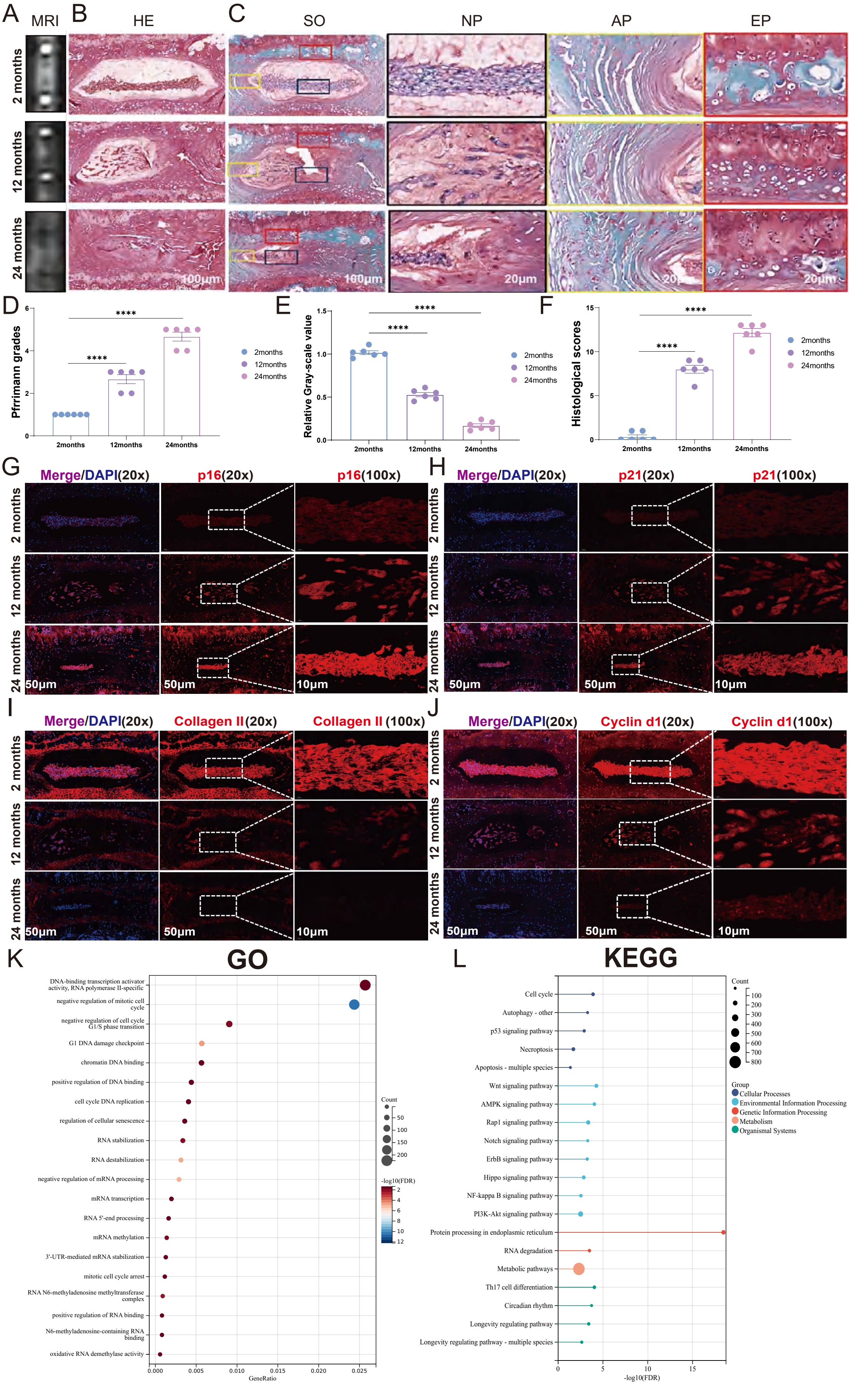

A MRI
B HE
C SO
NP
AP
EP
2 months
12 months
24 months
100μm
20μm
D
Pfrrimann grades
****
2months
12months
24months
E
Relative Gray-scale value
F
Histological scores
G
Merge/DAPI(20x)
p16(20x)
p16(100x)
H
p21(20x)
p21(100x)
50μm
10μm
I
Collagen II(20x)
Collagen II (100x)
J
Cyclin d1(20x)
Cyclin d1(100x)
K
GO
DNA-binding transcription activator activity, RNA polymerase II-specific
negative regulation of mitotic cell cycle
negative regulation of cell cycle G1/S phase transition
G1 DNA damage checkpoint
chromatin DNA binding
positive regulation of DNA binding
cell cycle DNA replication
regulation of cellular senescence
RNA stabilization
RNA destabilization
negative regulation of mRNA processing
mRNA transcription
RNA 5'-end processing
mRNA methylation
3'-UTR-mediated mRNA stabilization
mitotic cell cycle arrest
RNA N6-methyladenosine methyltransferase complex
positive regulation of RNA binding
N6-methyladenosine-containing RNA binding
oxidative RNA demethylase activity
GeneRatio
Count
-log10(FDR)
L
KEGG
Cell cycle
Autophagy - other
p53 signaling pathway
Necroptosis
Apoptosis - multiple species
Wnt signaling pathway
AMPK signaling pathway
Rap1 signaling pathway
Notch signaling pathway
ErbB signaling pathway
Hippo signaling pathway
NF-kappa B signaling pathway
PI3K-Akt signaling pathway
Protein processing in endoplasmic reticulum
RNA degradation
Metabolic pathways
Th17 cell differentiation
Circadian rhythm
Longevity regulating pathway
Longevity regulating pathway - multiple species
-log10(FDR)
Count
Group
Cellular Processes
Environmental Information Processing
Genetic Information Processing
Metabolism
Organismal Systems

Suplementary Figure 4

（A, D, E）MRI detection of T2-weighted signal intensity of intervertebral discs at different months of age in naturally aging mice; N=6 biologically independent replicates. Scale bars=100 μm and 20 μm.（B, C, F）H&E and Safranin-O staining of intervertebral discs of naturally aging mice at different months of age; N=6 biologically independent replicates. Scale bar=100 μm and 20 μm.（G-J）Immunofluorescence detection of p16, p21, Collagen II and Cyclin d1 proteins in the intervertebral discs of naturally aging mice at different ages. (K) GO pathway enrichment analysis of differentially expressed genes in subpopulations of $ELF1^{+}$ versus $ELF1^{-}$ cells; (L) Enrichment analysis of the KEGG pathway for differentially expressed genes in subpopulations of $ELF1^{+}$ versus $ELF1^{-}$ cells;Data presented as mean ± SD. One-way ANOVA was used for comparison among multiple groups. *P<0.05; **P<0.01; ***P<0.001; ****P<0.0001.

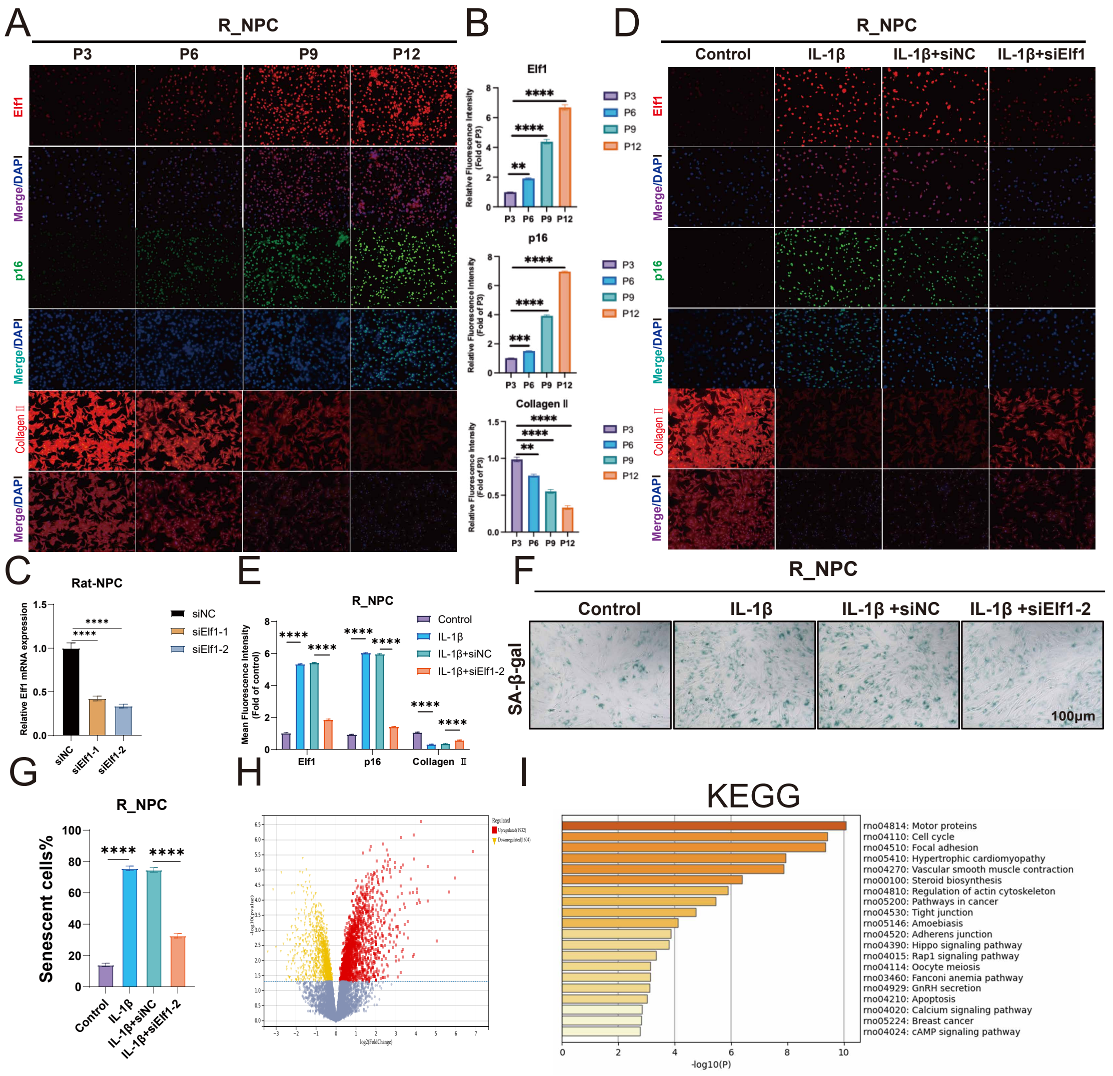

Supplementary Figure 5

(A, B) Immunofluorescence detection of Elf1 expression in R_NPC replicative senescence model; N=3 biologically independent replicates; Scale bar=50μm; (C) qPCR verified the knockdown efficiency of Elf1 siRNA in R_NPC; (D, E) Immunofluorescence was used to detect the protein expression levels of p16 and Collagen II following the addition of Elf1 siRNA after 10 ng/ml IL-1β treatment of R_NPC for 48 hours; N=3 biologically independent replicates; Scale bar=50μm; (F, G) Representative images of SA-β-gal staining and quantification of R_NPC

after 10 ng/ml IL-1β treatment for 48 h followed by the addition of Elf1 siRNA; N=3 biologically independent replicates; Scale bar=100μm; (H) Volcano plot showing R_NPC knockdown of Elf1 followed by RNA-seq of the differential gene; (I) KEGG enrichment analysis of differential genes after knockdown of Elf1; Data presented as mean ± SD. One-way ANOVA was used for comparison among multiple groups. *P < 0.05; **P < 0.01; ***P < 0.001; ****P < 0.0001.

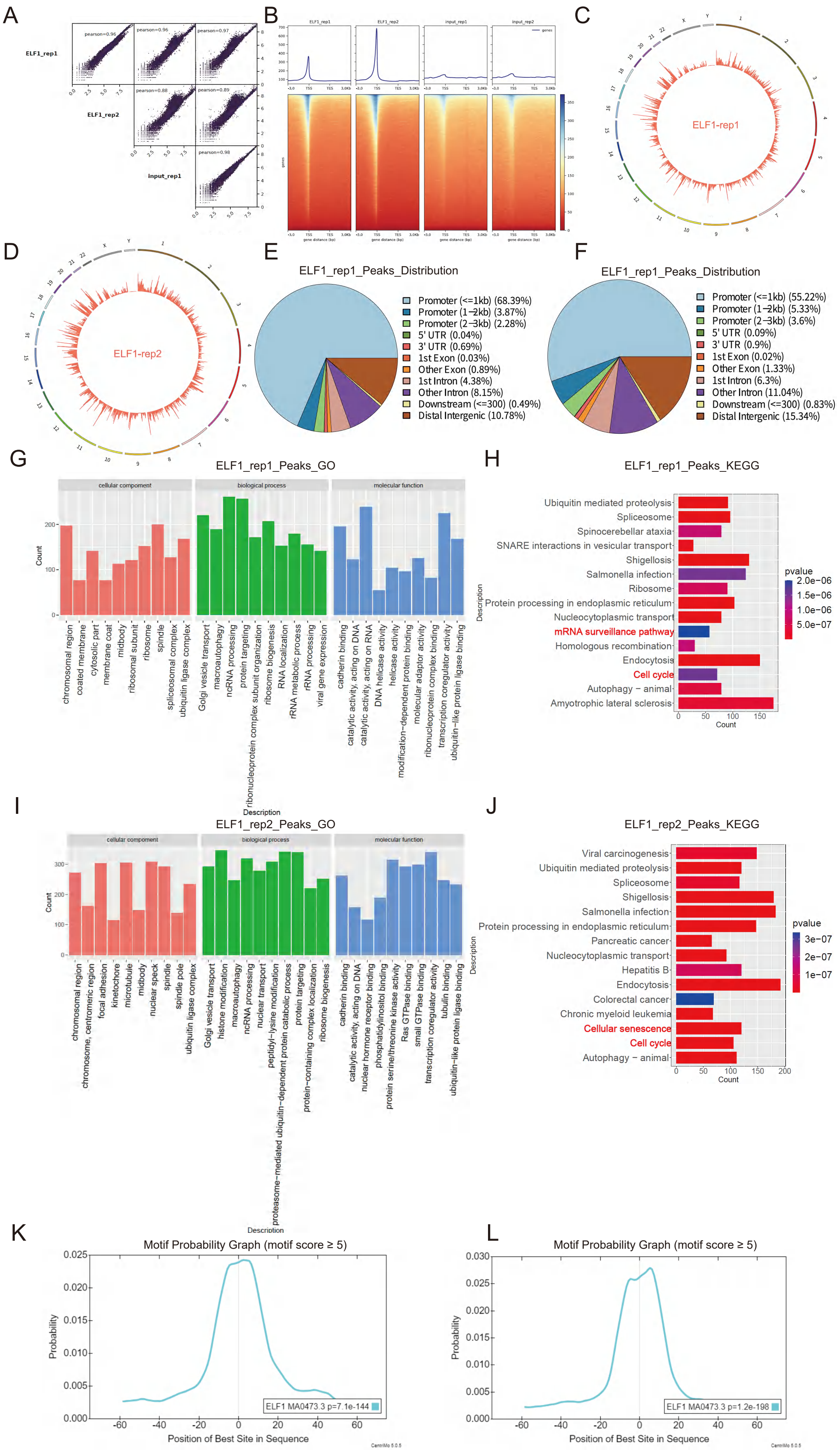
A
ELF1_rep1
ELF1_rep2
input_rep1
pearson=0.96
pearson=0.96
pearson=0.97
pearson=0.88
pearson=0.89
pearson=0.98
B
ELF1_rep1
ELF1_rep2
input_rep1
input_rep2
TSS
TES
gene distance (bp)
C
ELF1-rep1
D
ELF1-rep2
E
ELF1_rep1_Peaks_Distribution
Promoter (<=1kb) (68.39%)
Promoter (1−2kb) (3.87%)
Promoter (2−3kb) (2.28%)
5' UTR (0.04%)
3' UTR (0.69%)
1st Exon (0.03%)
Other Exon (0.89%)
1st Intron (4.38%)
Other Intron (8.15%)
Downstream (<=300) (0.49%)
Distal Intergenic (10.78%)
F
ELF1_rep1_Peaks_Distribution
Promoter (<=1kb) (55.22%)
Promoter (1−2kb) (5.33%)
Promoter (2−3kb) (3.6%)
5' UTR (0.09%)
3' UTR (0.9%)
1st Exon (0.02%)
Other Exon (1.33%)
1st Intron (6.3%)
Other Intron (11.04%)
Downstream (<=300) (0.83%)
Distal Intergenic (15.34%)
G
ELF1_rep1_Peaks_GO
cellular component
biological process
molecular function
Count
chromosomal region
coated membrane
cytosolic part
membrane coat
midbody
ribosomal subunit
ribosome
spindle
spliceosomal complex
ubiquitin ligase complex
Golgi vesicle transport
macroautophagy
ncRNA processing
protein targeting
ribonucleoprotein complex subunit organization
ribosome biogenesis
RNA localization
rRNA metabolic process
rRNA processing
viral gene expression
cadherin binding
catalytic activity, acting on DNA
catalytic activity, acting on RNA
DNA helicase activity
helicase activity
modification−dependent protein binding
molecular adaptor activity
ribonucleoprotein complex binding
transcription coregulator activity
ubiquitin−like protein ligase binding
Description
H
ELF1_rep1_Peaks_KEGG
Ubiquitin mediated proteolysis
Spliceosome
Spinocerebellar ataxia
SNARE interactions in vesicular transport
Shigellosis
Salmonella infection
Ribosome
Protein processing in endoplasmic reticulum
Nucleocytoplasmic transport
mRNA surveillance pathway
Homologous recombination
Endocytosis
Cell cycle
Autophagy − animal
Amyotrophic lateral sclerosis
Description
Count
pvalue
2.0e−06
1.5e−06
1.0e−06
5.0e−07
I
ELF1_rep2_Peaks_GO
cellular component
biological process
molecular function
Count
chromosomal region
chromosome, centromeric region
focal adhesion
kinetochore
microtubule
midbody
nuclear speck
spindle
spindle pole
ubiquitin ligase complex
Golgi vesicle transport
histone modification
macroautophagy
ncRNA processing
nuclear transport
peptidyl−lysine modification
proteasome−mediated ubiquitin−dependent protein catabolic process
protein targeting
protein−containing complex localization
ribosome biogenesis
cadherin binding
catalytic activity, acting on DNA
nuclear hormone receptor binding
phosphatidylinositol binding
protein serine/threonine kinase activity
Ras GTPase binding
small GTPase binding
transcription coregulator activity
tubulin binding
ubiquitin−like protein ligase binding
Description
J
ELF1_rep2_Peaks_KEGG
Viral carcinogenesis
Ubiquitin mediated proteolysis
Spliceosome
Shigellosis
Salmonella infection
Protein processing in endoplasmic reticulum
Pancreatic cancer
Nucleocytoplasmic transport
Hepatitis B
Endocytosis
Colorectal cancer
Chronic myeloid leukemia
Cellular senescence
Cell cycle
Autophagy − animal
Description
Count
pvalue
3e−07
2e−07
1e−07
K
Motif Probability Graph (motif score ≥ 5)
Probability
Position of Best Site in Sequence
ELF1 MA0473.3 p=7.1e-144
CentriMo 5.0.5
L
Motif Probability Graph (motif score ≥ 5)
Probability
Position of Best Site in Sequence
ELF1 MA0473.3 p=1.2e-198
CentriMo 5.0.5

Supplementary Figure 6

(A) Duplicate sample correlation analysis; (B) Gene body enrichment heatmap; (C, D) Peaks_position_circos plot: The Circos plot shows the positional distribution and enrichment degree of all Peaks on chromosomes, with the outer circle indicating the chromosome position, the middle circle indicating the degree of Peak enrichment in the Promoter region, and the inner circle indicating the degree of Peak enrichment in other regions; (E, F) Peaks_position_circos plot: The Circos plot shows the positional distribution and enrichment degree of all Peaks on chromosomes, with the outer circle indicating the chromosome position, the middle circle indicating the degree of Peak enrichment in the Promoter region, and the inner circle indicating the degree of Peak enrichment in other regions; (G, I) GO functional enrichment analysis of genes annotated by Peaks; (H, J) Peaks annotated genes for KEGG functional enrichment analysis; (K, L) Based on the calibration peaks and the annotated peaks, the 1000 Peaks with the largest fold change were filtered, and the Summit±75bp sequences of the Peaks were taken and analysed by Motif analysis using the MEME tool.

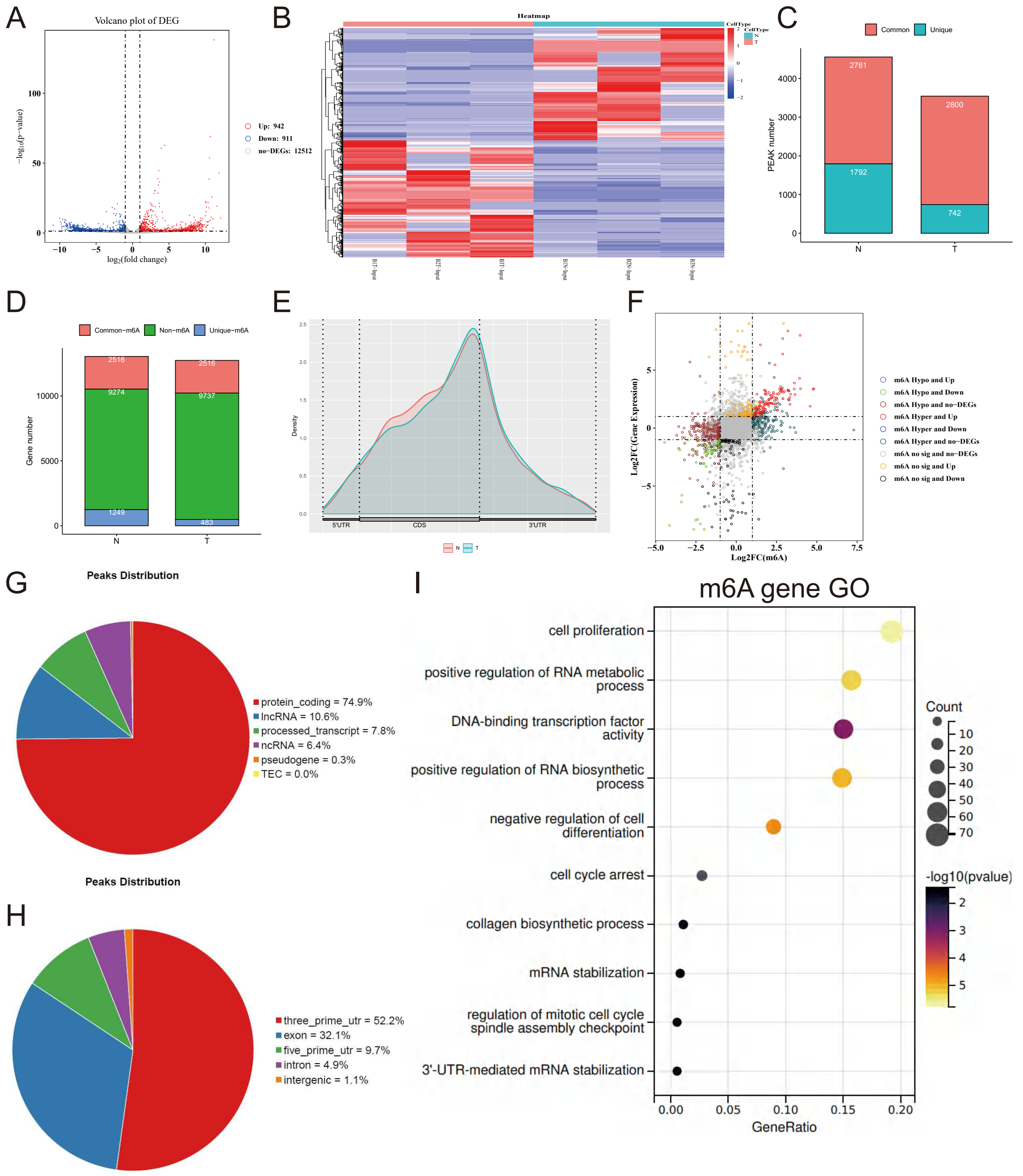

Supplementary Figure 7

(A) Volcano diagram showing differential genes in senescent H_NPC; (B) Heat map showing differential genes in senescent H_NPC; (C, D) PEAK and gene number analysis of Me-RIP-Seq results for H_NPC; (E) Metagene plot: the gene is divided into three segments, 5‘UTR, CDS and 3’UTR, and the distribution of peaks in each segment is counted; (F) Nine-quadrant plots demonstrating the correlation analysis between m6A levels

and mRNA expression in the results of the association analysis, with the criteria for this difference being P-value<0.05, FC>2 or FC< 0.5; (G) Peaks annotation to gene type statistics; (H) Annotation to gene location statistics; (I) GO enrichment analysis of m6A-modified genes.

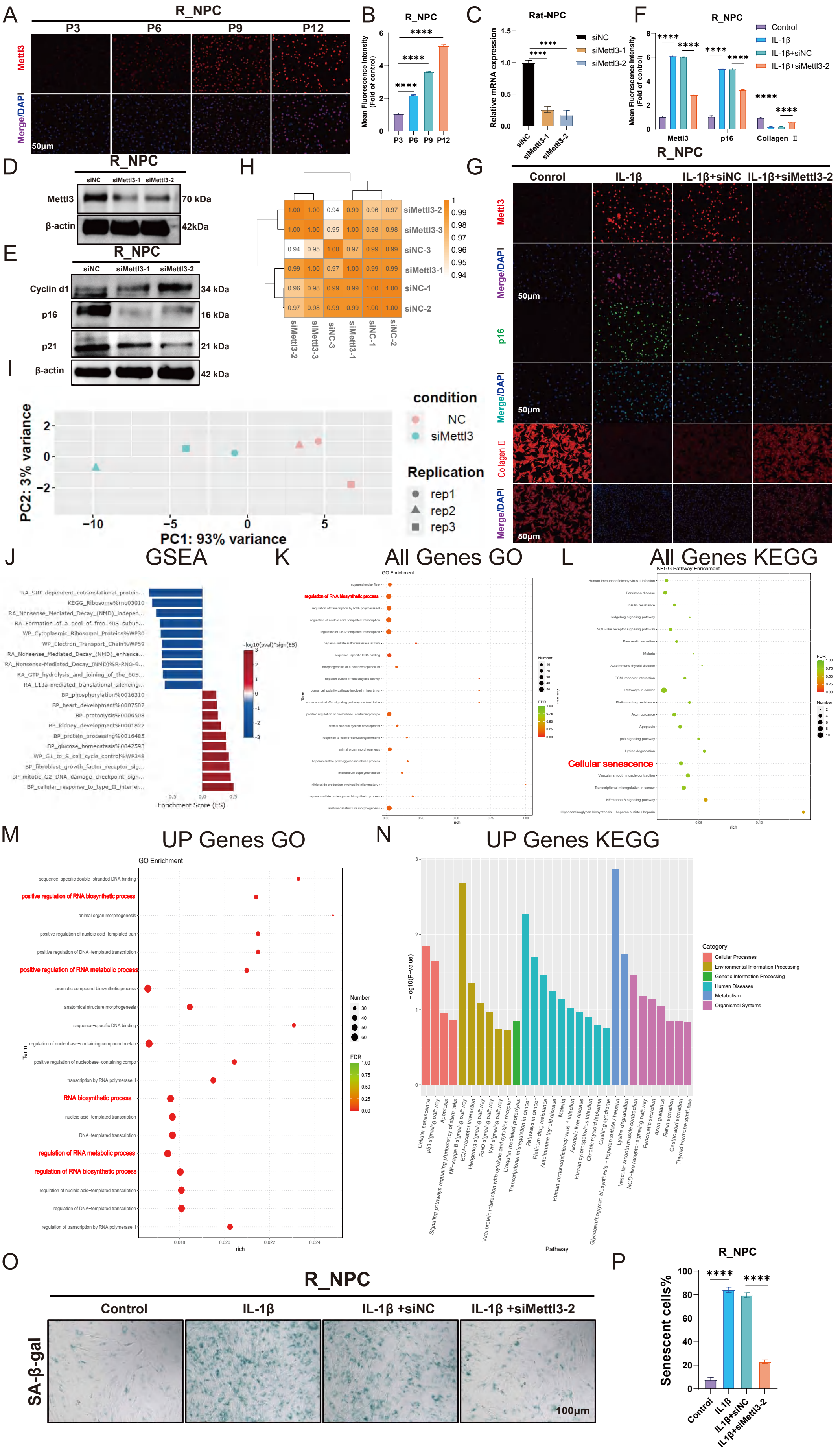

A
R_NPC
P3
P6
P9
P12
Mettl3
Merge/DAPI
50μm
B
R_NPC
Mean Fluorescence Intensity (Fold of control)
P3
P6
P9
P12
C
Rat-NPC
Relative mRNA expression
siNC
siMettl3-1
siMettl3-2
F
R_NPC
Control
IL-1β
IL-1β+siNC
IL-1β+siMettl3-2
Mean Fluorescence Intensity (Fold of control)
Mettl3
p16
Collagen Ⅱ
R_NPC
D
R_NPC
siNC
siMettl3-1
siMettl3-2
Mettl3
β-actin
70 kDa
42kDa
E
R_NPC
Cyclin d1
p16
p21
β-actin
34 kDa
16 kDa
21 kDa
42 kDa
H
siMettl3-2
siMettl3-3
siNC-3
siMettl3-1
siNC-1
siNC-2
G
Conrol
IL-1β
IL-1β+siNC
IL-1β+siMettl3-2
p16
Collagen Ⅱ
I
PC2: 3% variance
PC1: 93% variance
condition
NC
siMettl3
Replication
rep1
rep2
rep3
J
GSEA
Enrichment Score (ES)
-log10(pval)*sign(ES)
K
All Genes GO
GO Enrichment
regulation of RNA biosynthetic process
L
All Genes KEGG
KEGG Pathway Enrichment
Cellular senescence
M
UP Genes GO
GO Enrichment
positive regulation of RNA biosynthetic process
positive regulation of RNA metabolic process
RNA biosynthetic process
regulation of RNA metabolic process
regulation of RNA biosynthetic process
Term
rich
Number
FDR
N
UP Genes KEGG
-log10(P-value)
Pathway
Category
Cellular Processes
Environmental Information Processing
Genetic Information Processing
Human Diseases
Metabolism
Organismal Systems
O
R_NPC
Control
IL-1β
IL-1β +siNC
IL-1β +siMettl3-2
SA-β-gal
100μm
P
R_NPC
Senescent cells%
Control
IL-1β
IL-1β+siNC
IL-1β+siMettl3-2

Supplementary Figure 8

(A, B) Immunofluorescence detection of Mettl3 protein expression levels in rat NPC; (C, D) qPCR and Western blot assays for the efficiency of knockdown of Mettl3 in R_NPC; (E) Western blot detection of protein expression levels of Cyclin d1, p16 and p21 after knockdown of Mettl3; (F, G) Immunofluorescence was used to detect the protein expression levels of p16 and Collagen II after the addition of Mettl3 siRNA 48 h after 10 ng/ml IL-1β treatment; (H) Sample correlation tests; (I) PCA principal component analysis; (J) GSEA enrichment analysis of all differential genes for RNA-seq by knockdown of Mettl3 in R_NPC; (K, L) GO and KEGG enrichment analysis of all differential genes in RNA-seq after knockdown of Mettl3; (M, N) GO and KEGG enrichment analysis of all differential genes in RNA-seq after knockdown of Mettl3; (O, P) SA-β-gal staining was used to detect the effect of knockdown of Mettl3 on NPC senescence after 48 h of 10 ng/ml IL-1β treatment. Data presented as mean ± SD. One-way ANOVA was used for comparison among multiple groups. *P<0.05; **P<0.01; ***P<0.001; ****P<0.0001.

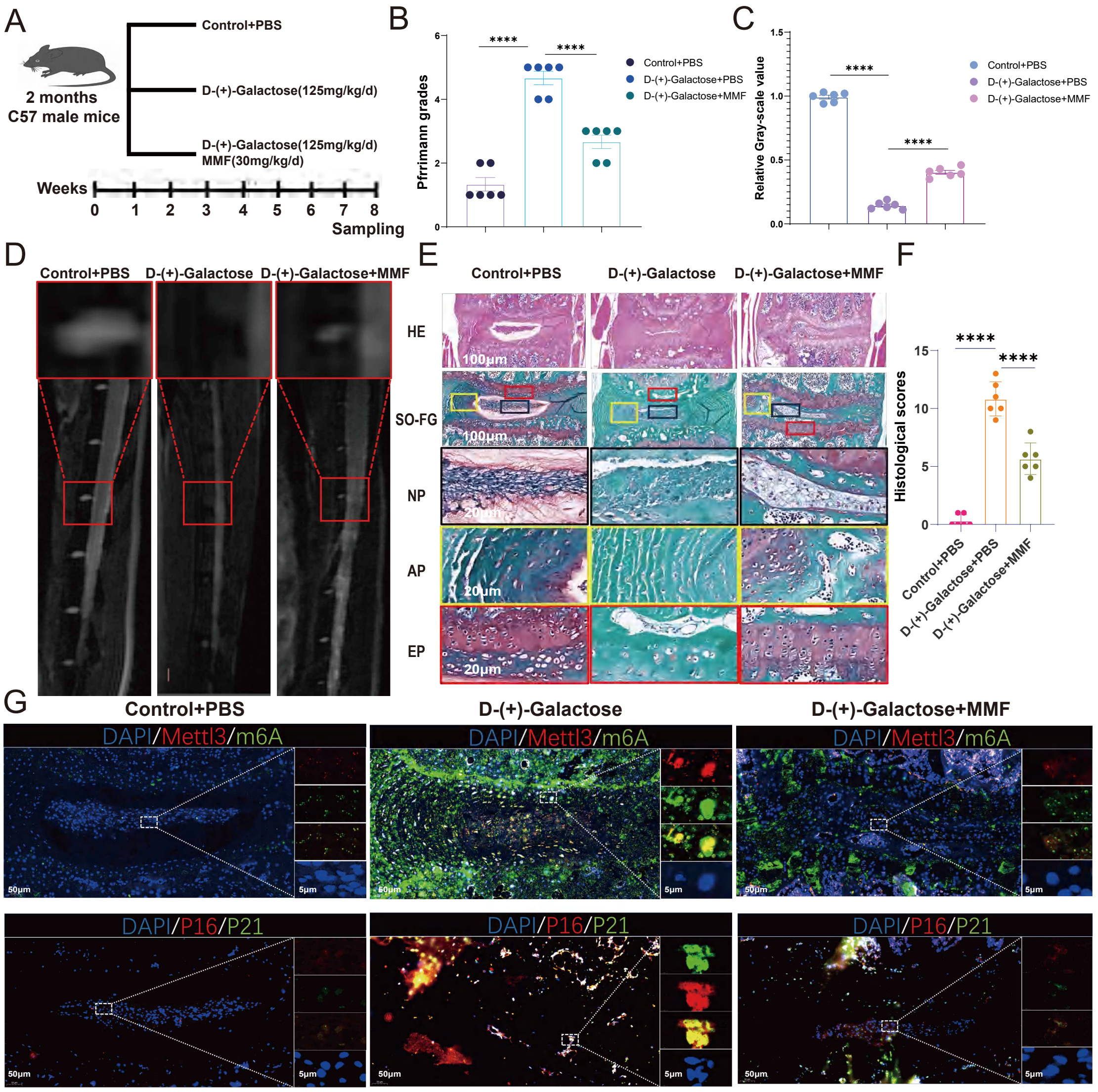

Supplementary Figure 9

(A) Experimental Flowchart. Juvenile (2-month-old, male, n=6) C57BL/6J mice were subjected to a model of NP tissue senescence induced by intraperitoneal injection of D-galactose (125 mg/kg/d), accompanied or unaccompanied by MMF (30 mg/kg/d)) oral treatment, and the lumbar vertebrae were collected for histological examination by MRI after 8 weeks of consecutive experiments followed by necropsy; (B-D) T2-weighted signal intensity of intervertebral discs in D-galactose-induced mouse aging model after treatment with MMF was detected using MRI; N=6; (E, F)

H&E and Safranin-O staining of mouse intervertebral discs after MMF treatment; N=6; Scale bar=100μm and 20μm; (G) Expression levels of Mettl3, p21, p16 and m6A in the NP tissue of MMF-treated mice were detected using immunofluorescence. Data presented as mean ± SD. One-way ANOVA was used for comparison among multiple groups. *P<0.05; **P<0.01; ***P< 0.001; ****P<0.0001.

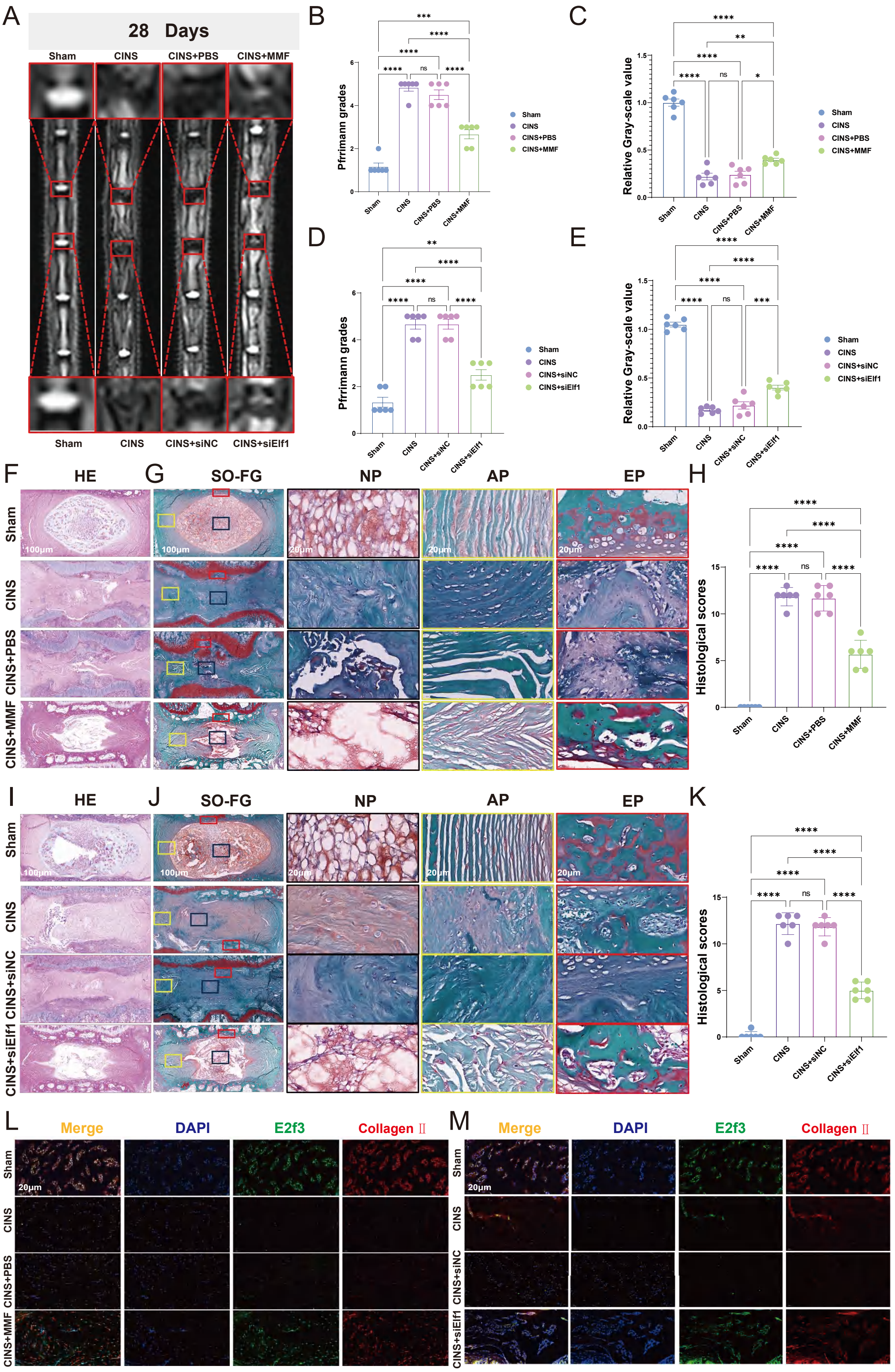
A
28 Days
Sham
CINS
CINS+PBS
CINS+MMF
CINS+siNC
CINS+siElf1
B
Pfrrimann grades
C
Relative Gray-scale value
D
E
F
HE
G
SO-FG
NP
AP
EP
H
Histological scores
I
J
K
L
Merge
DAPI
E2f3
Collagen Ⅱ
M

Supplementary Figure 10

(A-E) T2-weighted signal intensity of rat intervertebral discs after injection of MMF and Elf1 siRNA in the needling model of IVDD was detected using MRI; N=6；(F-H) H&E and Safranin-O staining of rat intervertebral discs after MMF treatment; N=6; Scale bar=100μm and 20μm; (I-K) H&E and Safranin-O staining of rat intervertebral discs after Elf1 siRNA treatment; N=6; Scale bar=100μm and 20μm; (L) Immunofluorescence detection of protein expression levels of E2f3 and Collagen II in NP tissue after MMF injection; (M) Immunofluorescence detection of protein expression levels of E2f3 and Collagen II in NP tissue after injection of Elf1 siRNA; Scale bar=20μm; Data presented as mean ± SD. One-way ANOVA was used for comparison among multiple groups. *$P<0.05$; **$P<0.01$; ***$P<0.001$; ****$P<0.0001$.

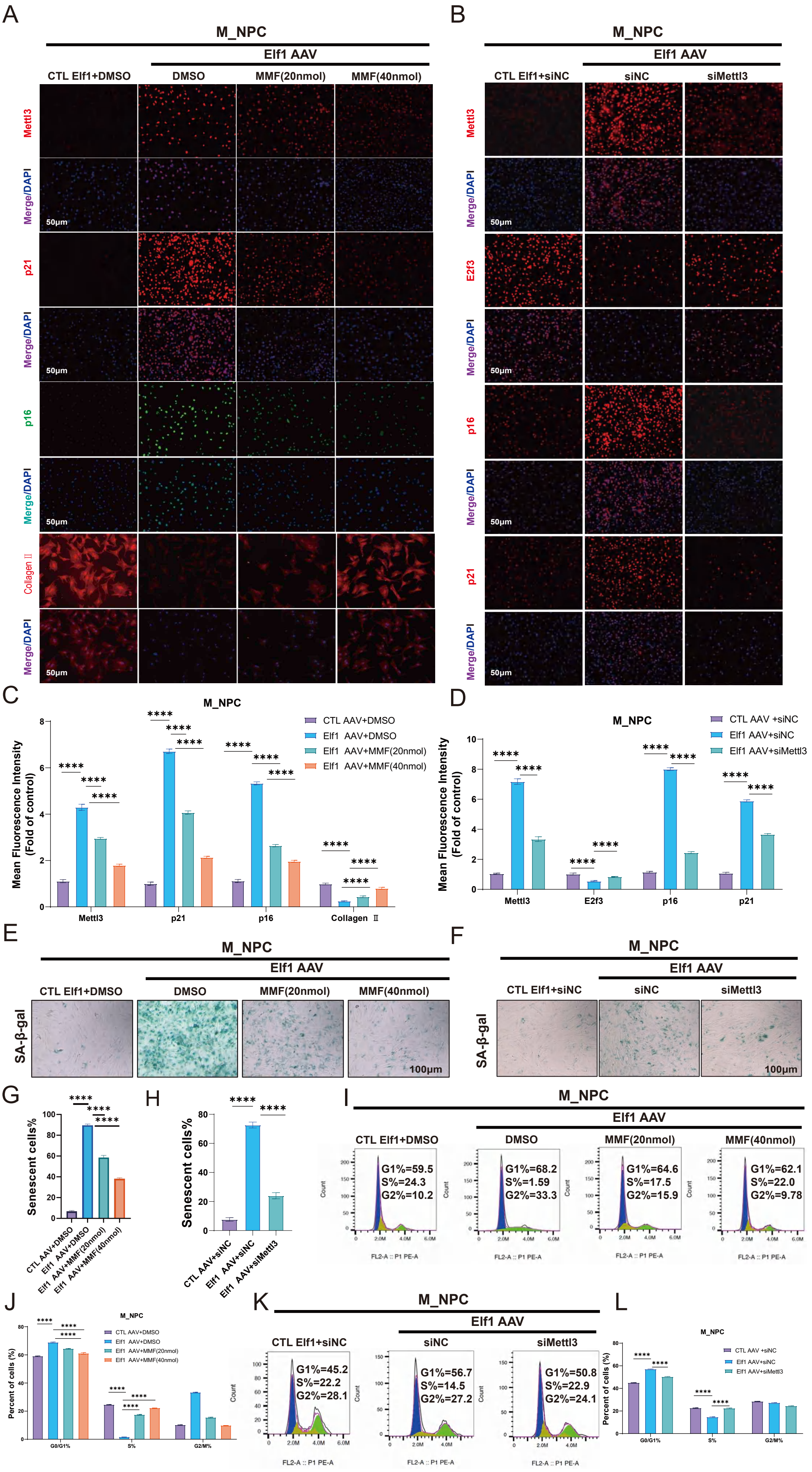

A
M_NPC
Elf1 AAV
CTL Elf1+DMSO
DMSO
MMF(20nmol)
MMF(40nmol)
Mettl3
Merge/DAPI
p21
p16
Collagen II
50μm
B
M_NPC
Elf1 AAV
CTL Elf1+siNC
siNC
siMettl3
Mettl3
Merge/DAPI
E2f3
p16
p21
50μm
C
M_NPC
CTL AAV+DMSO
Elf1 AAV+DMSO
Elf1 AAV+MMF(20nmol)
Elf1 AAV+MMF(40nmol)
Mean Fluorescence Intensity (Fold of control)
Mettl3
p21
p16
Collagen II
****
D
M_NPC
CTL AAV +siNC
Elf1 AAV+siNC
Elf1 AAV+siMettl3
Mean Fluorescence Intensity (Fold of control)
Mettl3
E2f3
p16
p21
****
E
M_NPC
Elf1 AAV
CTL Elf1+DMSO
DMSO
MMF(20nmol)
MMF(40nmol)
SA-β-gal
100μm
F
M_NPC
Elf1 AAV
CTL Elf1+siNC
siNC
siMettl3
SA-β-gal
100μm
G
Senescent cells%
CTL AAV+DMSO
Elf1 AAV+DMSO
Elf1 AAV+MMF(20nmol)
Elf1 AAV+MMF(40nmol)
H
Senescent cells%
CTL AAV+siNC
Elf1 AAV+siNC
Elf1 AAV+siMettl3
I
M_NPC
Elf1 AAV
CTL Elf1+DMSO
G1%=59.5
S%=24.3
G2%=10.2
DMSO
G1%=68.2
S%=1.59
G2%=33.3
MMF(20nmol)
G1%=64.6
S%=17.5
G2%=15.9
MMF(40nmol)
G1%=62.1
S%=22.0
G2%=9.78
Count
FL2-A :: P1 PE-A
J
M_NPC
CTL AAV+DMSO
Elf1 AAV+DMSO
Elf1 AAV+MMF(20nmol)
Elf1 AAV+MMF(40nmol)
Percent of cells (%)
G0/G1%
S%
G2/M%
K
M_NPC
Elf1 AAV
CTL Elf1+siNC
G1%=45.2
S%=22.2
G2%=28.1
siNC
G1%=56.7
S%=14.5
G2%=27.2
siMettl3
G1%=50.8
S%=22.9
G2%=24.1
Count
FL2-A :: P1 PE-A
L
M_NPC
CTL AAV +siNC
Elf1 AAV+siNC
Elf1 AAV+siMettl3
Percent of cells (%)
G0/G1%
S%
G2/M%

Supplementary Figure 11

(A, C) Immunofluorescence detection of protein expression levels of Mettl3, p21 and p16 in mouse nucleus pulposus cells (M_NPC) after overexpression of Elf1 followed by addition of MMF treatment; N=3; Scale bar=50μm; (B, D) Immunofluorescence detection of protein expression levels of Mettl3, E2f3, p21 and p16 in M_NPC after overexpression of Elf1 followed by addition of Mettl3 siRNA treatment; N=3; Scale bar=50μm; (E, G) SA-β-gal detects the number of senescent M_NPC after overexpression of Elf1 followed by addition of MMF treatment; N=3; Scale bar=100μm; (F, H) SA-β-gal detects the number of senescent M_NPC after overexpression of Elf1 followed by treatment with Mettl3 siRNA; N=3; Scale bar=100μm; (I, J) Flow cytometry was used to detect cell cycle progression after addition of MMF treatment following overexpression of Elf1 in M_NPC; (K, L) Flow cytometry was used to determine the progression of the cell cycle after the addition of Mettl3 siRNA treatment following the overexpression of Elf1 in M_NPC; Data presented as mean ± SEM. One-way ANOVA was used for comparison among multiple groups. *P<0.05; **P<0.01; ***P<0.001; ****P<0.0001.

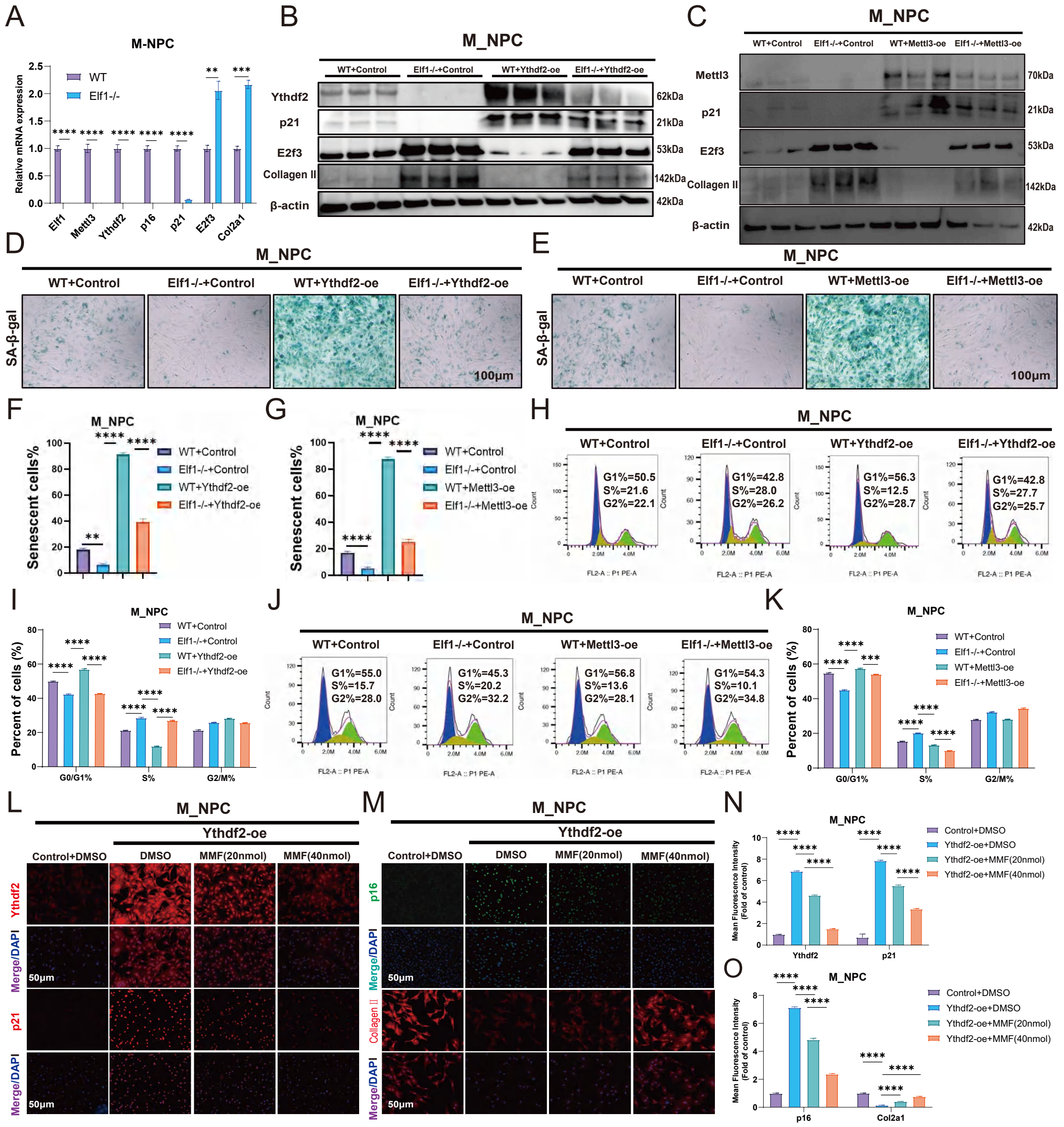

Supplementary Figure 12

(A) qPCR for mRNA expression of Mettl3, Ythdf2 and E2f3 after Elf1 KO; (B) Protein expression levels of Ythdf2, p21, E2f3 and Collagen II after overexpression of Ythdf2 in M_NPC following Elf1 KO were detected using Western blot; (C) Protein expression levels of Mettl3, p21, E2f3 and Collagen II were detected using Western blot after overexpression of Mettl3 in M_NPC following Elf1 KO; (D, F) SA-β-gal detects the number of senescent cells after overexpression of Ythdf2 in M_NPC after Elf1 KO;

N=3; Scale bar=100μm; (E, G) SA-β-gal detects the number of senescent cells after overexpression of Mettl3 in M_NPC after Elf1 KO; N=3; Scale bar=100μm; (H, I) Flow cytometry showing cell cycle progression after overexpressing Ythdf2 in M_NPC following Elf1 KO; (J, K) Flow cytometry showing cell cycle progression after overexpressing Mettl3 in M_NPC following Elf1 KO; (L-O) Immunofluorescence was used to detect the protein expression levels of Ythdf2, p16, p21 and Collagen II after overexpression of Ythdf2 followed by addition of MMF treatment. Data presented as mean ± SD. One-way ANOVA was used for comparison among multiple groups. *P<0.05; **P<0.01; ***P<0.001; ****P<0.0001.

Materials and methods

2.1. Acquisition of IVDD Datasets

Two IVDD-related high-throughput RNA sequencing datasets, GSE34095 and GSE56081, were obtained from the Gene Expression Omnibus (GEO: https://www.ncbi.nlm.nih.gov/geo/) database in this study. The pre-processed datasets mentioned above were consolidated and any batch effects were taken into account using the sva R package, in particular by using the ComBat function. Single-cell RNA sequence data (scRNA-seq) were obtained from the Gene Expression Omnibus (GEO) database GSE165722 and analysed using the Seurat package in R (v4.3.0). Once the data has been downloaded, it is normalised for the identification of highly variable genes and a scaling procedure is performed. Principal Component Analysis (PCA) was then performed on the scaled data and elbow diagram analysis determined the principal component count. In order to account for the batch effect between the degradation level groups (MDD groups for levels II and III and SDD groups for levels IV and V), the Harmony multi-data set integration algorithm was used. PCA embeddings were co-ordinated and visualised using a drop chart and a violin plot by merging 'group' variables. Uniform Mobility Approximation and Projection (UMAP) and t-distributed Stochastic Neighbourhood Embedding (t-SNE) were then applied to the coordinated embeddings to further analyse the data. Identify marker genes for all clusters and export results for further analysis. For

differential expression analysis between groups, combine cell type and degeneration grade group information for each cell.

2.2. Analysis of functional enrichment and analysis of gene set enrichment

We used the DAVID database, available at https://david.ncifcrf.gov/, to identify enriched biological terms associated with DEGs. GO terms and KEGG pathways were considered statistically significant if their p-values were below 0.05. For all of the results, graphical plots have been produced using the powerful ggplot2 package in R.

2.3. Patient tissue sample collection

We collected 30 fresh disc samples from patients undergoing spinal surgery at Dongfang Hospital, Tongji University, and assessed the degree of disc degeneration according to the Pfirrmann grading system. This study was approved by the ethics committees of Shanghai East Hospital, Tongji University School of Medicine and was conducted in accordance with the World Medical Association's Declaration of Helsinki (Approval No: 2022YS-045).

2.4 LC-MS/MS analysis

For proteomic profiling through mass spectrometry (MS), samples were subjected to isobaric tagging using the TMTsixplex™ Isobaric Label Reagent Set (Thermo Fisher Scientific, USA) in accordance with the manufacturer's specifications for the 6-plex TMT Kit. In brief, nucleus

pulposus (NP) specimens were treated with 50 μL of 100 mM triethylammonium bicarbonate buffer, homogenized via vortex mixing, and the labeling reaction was executed within a 1.5 mL Eppendorf tube. An aliquot of 88 μL anhydrous acetonitrile was subsequently introduced to the mixture, followed by 5 minutes of vortex agitation and centrifugation. Post-centrifugation, 41 μL of TMT reagent was administered to 100 μg of enzymatically digested NP samples. Differential labeling was achieved with TMT-126, TMT-127, and TMT-128 tags for the non-degenerated NP cohort, whereas the degenerated NP group was labeled with TMT-129, TMT-130, and TMT-131 tags. The samples were then subjected to agitation, centrifugation, and incubated at ambient temperature for 60 minutes. The labeling reaction was quenched by the addition of 5% hydroxylamine (8 μL) with a 15-minute incubation, succeeded by lyophilization for sample preservation. Chromatographic separation was conducted utilizing an Agilent 1100 series high-performance liquid chromatography (HPLC) system equipped with an Agilent Zorbax Extend-C18 column (2.1 × 150 mm; 5 μm particle diameter). Tagged protein samples were introduced to an Acclaim PepMap 100 RP-C18 column (100 μm × 2 cm; Thermo Fisher Scientific, Waltham, MA, USA) at a flow rate of 2 μL/min, with subsequent resolution on an Acclaim PepMap RSLC RP-C18 column (75 μm × 15 cm; Thermo Fisher Scientific, Waltham, MA, USA). For the analysis of peptide fractions, the mobile phases comprised eluent A (99.9%

aqueous solution with 0.1% formic acid) and eluent B (80% acetonitrile, 19.9% water, and 0.1% formic acid). Mass spectra were captured across a range of 350–1500 m/z with a resolution of 60,000, and the automatic gain control (AGC) target was calibrated to 3×10^6. The 20 most prominent ion peaks underwent fragmentation via higher-energy collisional dissociation (HCD) at a collision energy of 32. Tandem MS (MS/MS) spectra were obtained with a resolution of 15,000, an AGC target of 2×10^5, and a maximum ion injection time of 40 milliseconds. A dynamic exclusion window of 30 seconds was applied, and all analyses were conducted in positive ionization mode.

2.5 Elf1 $^{-/-}$ mouse

Animal studies were performed using 7-8 weeks Elf1 knockout C57BL/6 (n = 6 per group) generated by Sayer (Suzhou, China) using CRISPR/Cas9-mediated genome editing. Mouse Elf1 gene gRNA and Cas9 mRNA were co-injected into fertilised eggs to generate targeted knockout progeny. F2 founder animals were identified for Elf1$^{-/-}$ by PCR and IVD tissues were immunofluorescently stained for Elf1 protein. All mice were housed in an SPF environment with constant temperature (23-25°C) and humidity (50%) and a circadian rhythm of 12 h light/12 h dark. The following primer sets were used to determine the genotypes of (+/-) heterozygous mice that can be self-bred to produce (-/-) heterozygous mice. Primer set 1: F1: 5‘-TCATCCTAAAGGAGGACAGCAG-3’; R1: 5‘-

CCATACATAGCCTCACATACCACTG-3’; Target gene knockdown positive. 647 bp; primer set 2: F2: 5‘-AGATTCTAGTTGTGGTGCCCTTG-3’ R2: 5‘-CTCCCTCCAGAACACCATGCTATC-3’; Target gene not knocked out negative: 716 bp. (-/-) Pure heterozygote: 647 bp; (+/-) Heterozygote: 647 bp, 716 bp; (WT) Wild type: 716 bp. All animal and human experiments were reviewed and approved by the Ethics Committee of School of Medicine, Tongji University .

2.6 Cultivating, transfecting and treating NPC in vitro

(1) Extraction of Human-derived Nucleus pulposus cell (H_NPC): The Nucleated Pulposus tissue was digested sequentially with 0.2% Pronase and 65 U/ml type 2 collagenase solution (Worthington, cat. #LS004177). Released cells were filtered through a 100 μm cell filter and collected by centrifugation at 500 g for 5 min. Cells were then inoculated into culture flasks and cultured in minimum essential medium α (α-MEM; Gibco, cat. #11900-073) (Corning, cat. #35-010-CV) containing 10% foetal bovine serum and maintained in a humidified incubator at 37°C, 2% CO_2. When cells grew to 80% confluence, they were digested with 0.05% trypsin/0.48 mM EDTA (Gibco, cat. #15400054) and passaged for amplification. The P3 cells were used for further experiments. **(2) Extraction of Rat nucleus pulposus cells (R_NPC):** Isolation of R_NPC from adult SD rats. Same method as above. **(3) Extraction of mouse nucleus pulposus cells**

(M_NPC): The mice were first anaesthetised with 1% sodium pentobarbital to obtain M_NPC from Elf1$^{-/-}$ mice. Mice were then subjected to decapitation and execution. The lumbar spine of the mouse was removed from the aseptic operating table after immersion in 75% ethanol for 5 minutes, remove NP tissue, place in 15ml centrifuge tubes containing 0.25% type II collagenase, shaker at 37°C, 100rpm, digestion for 5hrs, 1000rpm, centrifuge for 5min, Resuspend in complete medium, place in a cell culture dish and incubate in a cell culture flask at 5% CO2/37°C. The medium was changed every 3 d after cell attachment to the wall. When 80% fusion was achieved, the cells underwent passage 1:(2-3). **(4) For IL-1β treatment:** H_NPC or R_NPC were inoculated at a density of 2×10^{6} cells/well into 6-well cell plates and treated with or without 10 ng/ml IL-1β (PeproTech, Catalogue #200-01B). For the H2O2 treatment, 200 μM of H2O2 was added to the NPC and the medium was changed after 2 hours. For the treatment with Mycophenolate Mofetil (APExBIO Technology, catalogue no. A4336), primary H_NPC or R_NPC were treated with 0, 20 or 40 nM Mycophenolate Mofetil. **(5)** Nucleus pulposus cells were first plated in 6-well plates and incubated for 24 hours to allow cell attachment and stabilisation in preparation for siRNA transfection. Transfection was then performed using Lipofectamine 2000 as the transfection reagent and siRNA at a dose of 100 pmol per well. This 12-hour transfection was performed. NPCs were subjected to a series of

analytical procedures, including RNA and protein extraction, following a 48-hour post-transfection interval. Target sequences were as follows: Supplementary Table 1. **(6) For Elf1 AAV5 infection:** M_NPC were infected with Elf1AAV5 expressing enhanced green fluorescent protein (Elf1 AAV5-eGFP) as control AAV5 or Elf1 AAV5 (GPAAV-CMV-Mouse Elf1-3xFlag-T2A-eGFP-WPRE) at a MOI of 100.

2.7. MeRIP-qPCR

Total RNA was extracted and purified from R_NPC using the RiboBio RiboMethylation RNA Immunoprecipitation (RiboMeRIP) m6A Transcriptome Profiling Kit (10 assays) (Cat. No. C11051-1). To enrich and purify RNA fragments with m6A modifications, A/G magnetic beads and m6A methylation antibody were pre-mixed and incubated with the RNA fragments. The relative expression of the target gene was determined by qPCR after reverse transcription of the RNA and cDNA. The relative abundance of m6A modification in the target gene RNA was expressed as input %.

2.8. RNA pulldown and RNA stability detection

A biotin-labeled mRNA probe for E2f3 (Biontin-GCAG**a**CUGAGGACCAAAUUCCUUCCAACCUUGAAGG**a**CCUUU) was purchased from Genomeditech (Shanghai, China). In vitro transcription experiments were performed using the BersinBioTM RNA Pulldown Kit (Bersin, Bes5102). RNA probes were labelled. RNA-protein

complexes were then formed by incubating the labelled probes with whole-cell protein extracts. The complex is able to bind to streptavidin-labelled magnetic beads, allowing it to be separated from the other components of the incubation solution. After elution of the complex, whether specific RNA-binding proteins interacted with the RNA was determined by Western blot experiments. For the evaluation of E2f3 stability, cells were treated with actinomycin (5 μg/ml) for transcription arrest. After transcription was stopped, samples were taken at 0, 30, 60 and 90 minutes. Total RNA was extracted and the expression of E2f3 was measured by quantitative real-time PCR with fluorescence detection.

2.9. IVDD rat models

Ten adult Sprague-Dawley rats, each 8 weeks old, were included in the current study. These subjects were distributed at random, with an equal number of subjects in the control group and in the group designated for the IVDD research. Protocols established by previous research were used to select the C7/8 discs for study. The rats were then placed in a prone position under a high-resolution MRI system (PHILIPS-Achieva 3.0 T, The Netherlands) to take the necessary images. Briefly, the rat was anaesthetised. A 21-gauge needle was inserted 3.0 mm into the caudal disc of the rat. An IVDD model was established for a duration of 30 seconds. Mycophenolate mofetil and PBS were administered at a dose of 30 mg/kg in the needling group by intradiscal injection twice a week for 4 weeks.

Meanwhile, si-Elf1 and siNC chemically modified with 5Col/2OMe were synthesised by RiboBio (Guangzhou, China) for intradiscal injection of 5 nmol (10 μL) in the needle IVDD model after the knockdown efficiency of si-Elf1 was verified by transfection of R_NPC.

2.10. Administering drugs to aged mice

Young and naturally senescent wild-type C57BL/6J mice (2 months old, males, n=6; 18 months old, males, n=12) were housed in a standard Specific Pathogen Free (SPF) laboratory with controlled temperature (20-26°C), humidity (range 50% to 60%) and a 12-hour light/dark cycle. They were given food and water ad libitum. Each experimental group was randomly assigned to mice. Prior to dosing, the mice were kept for one week in the animal facility. For oral administration, mice were given mycophenolate mofetil (30 mg/kg/d) or a control diet of CMC-Na (Solarbio Life Sciences, Cat. IS9000) three times a week for 6 months, until the mice were euthanised with isoflurane. For Mycophenolate Mofetil to ameliorate disc degeneration due to D-galactose in mice the following subgroups were performed: Control group (2 months); D-Galactose group: D-Galactose was administered via intraperitoneal injection at a concentration of 30 mg/kg/d for 8 weeks; D-Galactose + Mycophenolate Mofetil group: Mycophenolate Mofetil was administered via oral tube feeding at a concentration of 30 mg/kg for 8 weeks.

2.11. Histology and Immunostaining assays

Human NP samples were fixed in 4% paraformaldehyde (PFA) for 48 h, dehydrated, paraffin-embedded and sectioned at 5 μm. The sections were subjected to IHC and IF staining. Mouse IVDs were fixed for 48 hours in 4% PFA, decalcified for 14 days in 10% EDTA (pH 7.2), dehydrated, embedded in paraffin and sectioned at 5 μm prior to staining. Rat IVDs were fixed in 4% PFA for 48 hours, followed by decalcification in 10% EDTA for 8 weeks. The IVDs were dehydrated, embedded in paraffin and sectioned at 5μm. The prepared sections were stained with Safranin O and Fast Green (SO&FG). The histological scores of the IVDs were scored according to the Histological Scoring System for IVDs as previously described (PMID: 28636254). For IHC staining, the sections were deparaffinised in xylene and then rehydrated in a graded series of ethanol. Citrated buffer (0.1 mol/L, pH 6.0) was used for antigen retrieval. After blocking with peroxidative blocking solution and normal horse serum, sections were incubated with primary antibodies (4°C, overnight). The sections were then incubated with biotinylated IgG and streptavidin-horseradish peroxidase. Immunoreactivity was visualised using the DAB peroxidase substrate kit. Finally, the sections were counterstained with haematoxylin and then mounted. Sections were prepared for IF staining in the same way as for IHC staining. Following blocking with QuickBlock™ buffer (Beyotime) supplemented with Triton 100 (Sigma-Aldrich), sections were incubated with primary antibodies (4°C, overnight). The sections

were then incubated with anti-mouse or anti-rabbit Alexa Fluor 488 or 568 secondary antibodies.

2.12. X-ray Film and MRI Analyses

The height of the intervertebral space was assessed using X-ray film (uDR 588i, United Imaging, Shanghai, PR China). The analysis of intervertebral disc height reduction was conducted by calculating the Disk Height Index (DHI), using the following equation: DHI%=(D+E+F) *100%/(A+B+C+D+E+F). In this formula, A, B and C are the heights of the anterior, middle and posterior edges of the upper vertebral body, respectively. D, E and F are the analogous measurements for the intervertebral space. A 3.0 T MRI scanner (PHILIPS-Achieva 3.0 T, The Netherlands) was also used to assess the degree of IVDD. The disc was measured using quantitative T2 mapping in the sagittal plane of the MRI. The Picture Archiving and Communication System region of interest (ROI) was used to assess the midsagittal plane of the disc by drawing contours.

2.13. RNA extraction and quantitative PCR

Strictly following the manufacturer's guidelines, we performed RNA extraction using TRIzol reagent (manufactured by Beyotime, Shanghai, China). Quantitative analysis was performed using SYBR Green master mixes (provided by Yeasen, China) and the 7500 PCR system, with 40 amplification cycles. For quantification of relative gene expression, we used the $2^{-\Delta\Delta CT}$ method for accurate measurement. The primer sequences

used for qPCR analysis can be found in Supplementary Table 2.

2.14 Western blot analysis and Immunofluorescence Staining

Protein extracts were prepared using RIPA buffer, supplemented by protease and phosphatase inhibitors for optimal conservation. Protein levels were quantified using the bicinchoninic acid (BCA) protein assay kit (#ZJ101, supplied by Epizyme, China). The immunoblots were incubated with antibodies in the following way: ELF1 (Proteintech, 22565-1-AP), YTHDF2 (Proteintech, 24744-1-AP), METTL3 (Proteintech, 15073-1-AP), P21 (Proteintech, 10355-1-AP), MMP13 (Proteintech, 18165-1-AP), P16 (Proteintech, 10883-1-AP), ADAMTS5 (Abclonal, A23125), E2F3 (Abclonal, A8811), Collagen II (Abclonal, A19308), Cyclin D1(Abclonal, A1301). Protein bands were finally visualized with enhanced chemiluminescence (ECL) kit. GAPDH (Abclonal, A19056) or β-Actin (Abclonal, AC026) was used as internal control. Cells were fixed with 4% paraformaldehyde for 15 minutes at 4°C, followed by incubation in Triton X-100 and 5% goat serum (P0096, C0265, Beyotime). This was followed by overnight incubation with primary antibodies at 4°C. Samples were then incubated with FITCgoat anti-rabbit IgG (AB clonal, AS011) or Cy3goat anti-rabbit IgG (AB clonal, AS007) for 1 hour in the dark. All images were taken after incubation of cells in DAPI (C1006, Beyotime) for 5 min in the dark using the same microscope (Leica DMI 6000B, Germany).

2.15 Chromatin immunoprecipitation (ChIP) assay and Luciferase

reporter gene assay

Chip sequencing data, including specific entries such as GSM803415, GSM1010765 and GSM999185, were obtained from the Gene Expression Omnibus (GEO). The 2000 base pair region upstream of METTL3 and YTHDF2 genes was selected as promoter region according to information from the National Center for Biotechnology Information database (http://www.ncbi.nlm.nih.gov/). We then designed ChIP primers to target these predicted sites, using Primer Premier to design them. ChIP assays were then performed in conjunction with an ELF1 antibody (22565-1-AP) from Proteintech using a Beyotime ChIP kit (Catalogue P2083S, China). Following these assays, the degree of binding of ELF1 to the METTL3 and YTHDF2 promoters was quantified by PCR, and the PCR-amplified DNA was resolved on agarose gels and the products were visualised by digital imaging. Genomeditech Company (Shanghai, China) synthesised WT and mutant METTL3 and YTHDF2 gene promoter fragments. It was then cloned into the PGL3-basic luciferase vector to obtain PGL3-basic-H_METTL3 promoter (-2000 to +50) WT and PGL3-basic-H_METTL3 promoter (-2000 to +50) MT plasmids. Then it was cloned into PGL3-basic luciferase vector to obtain PGL3-basic-H_YTHDF2 promoter (-1950 to+50 bp) WT and PGL3-basic-H_ YTHDF2 promoter (-1950 to+50 bp) MT plasmids， were transfected with Lipofectamine2000 (Thermo Fisher Scientific, Massachusetts, USA), respectively. After transfection, the cells

were cultured in normal medium for 48 h. The cells were harvested and lysed for the luciferase assay and the luciferase activity was normalised to the Renilla luciferase activity.

2.16 Cell Cycle Assay and Senescence-Associated β-Galactosidase Assay

Cells were placed in 1.5 ml Eppendorf tubes, washed twice with phosphate-buffered saline (PBS), centrifuged and resuspended in pre-cooled 70% ethanol. The cells were fixed. After 30 min staining at room temperature, samples were passed through 300 mesh nylon membranes. The cell cycle was analysed by flow cytometry using a cell cycle analysis kit (C1052, Beyotime). The beta-galactosidase staining kit (C0602, Beyotime) was used to detect the activity of SA-β-gal. The cells were fixed in a colour fixative for 15 minutes at room temperature. The samples are incubated overnight at 37°C with the addition of 1 mL of working solution after three washes with PBS.

2.17. Molecular docking

Use Open Babel, a powerful computational tool for chemical structure manipulation, to generate 3D structures of compounds. To ensure accurate representation for subsequent docking simulations, receptor proteins and ligands are carefully prepared and parameterised using AutoDock Tools (ADT3). Subsequent docking simulations were performed using AutoDock Vina (1.2.0). AutoDock Vina is a state-of-the-art molecular docking

software known for its accuracy and efficiency in predicting ligand-receptor interactions. AlphaFold 2 was used to predict the structure of ELF1. Modeling quality was assessed using predicted alignment error, where colour at position (x, y) indicates expected position error at residue x when predicted and real structures are aligned at residue y. Predicted structure was assessed using predicted alignment error. A database of 11036 compounds (LCO020020) was used to obtain MMF structures. Molecular docking simulations and analysis of the predicted structures of ELF1 and MMF were performed using AutoDockTools-1.5.6. Visualisation was performed using PyMOL 2.3.2.

2.18. EDU incorporation test

To analyse proliferating cells by flow cytometry, EdU-positive cells were analysed using a Cell-Light EdU Apollo 488 in vitroflow cytometry kit (RiboBio, Guangzhou, China) according to the manufacturer's protocol. A flow cytometer was used to measure the fluorescence signal at 488 nm.

2.19. RNA sequencing and analysis

(1) RNA extraction and assay, total RNA quality assay: Concentration and purity were measured by nanodrop (Thermo Scientific NanoDrop 2000 (Thermo Scientific, Waltham, Massachusetts, USA)), and integrity was measured by RNA-specific agarose electrophoresis or 2100 (Agilent 2100). Bioanalyzer. RNA 6000 Nano kit 5067-1511(Agilent Technologies Inc, California, USA). **(2) Library Construction and Quality Assurance:**

Total RNA >=1ug was selected and enriched for mRNA with polyA tails by Oligo(dT) magnetic beads followed by ionic interruption using a dibasic library using the NEB Next Ultra II RNA Library Prep Kit for Illumina (New England Biolabs Inc; Ipswich, Massachusetts, USA) (strand-specific library kit NEB Next Ultra Directional RNA Library Prep Kit for Illumina), mRNAs with polyA tails were enriched by Oligo(dT) magnetic beads, and the mRNAs were subsequently randomly interrupted by ion interruption using divalent cations. The cDNA was synthesised using fragmented mRNA as template and random oligonucleotides as primers. cDNA was purified from double-stranded cDNA, followed by double-end repair and introduction of the 'A' base at the 3' end and ligation of the sequencing junctions. The cDNA was screened for cDNAs of 400-500 bp using AMPure XP beads, amplified by PCR and the PCR products were purified again using AMPure XP beads to obtain the final library. Library quality testing was performed using an Agilent 2100 Bioanalyzer (Agilent Technologies, California, USA), Agilent High Sensitivity DNA Kit (Agilent Technologies, California, USA, 5067-4626). Total library concentration was detected using Pico green (Quantifluor-ST fluorometer, Promega Corporation, Madison, Wisconsin, USA, E6090; Quant-iT PicoGreen dsDNA Assay Kit, Invitrogen Corporation, California, USA, P7589). QPCR quantified the concentration of active libraries (StepOnePlus Real-Time PCR Systems, Thermo Scientific, Waltham,

Massachusetts, USA). Multiplexed DNA libraries are homogenised and mixed in equal volumes. The mixed libraries were progressively diluted and quantified and then sequenced in PE150 mode on an Illumina sequencer. Use HTSeq (v0.9.1) to statistically match the Read Count value to each gene as the raw gene expression. In order to make gene expression levels comparable across genes and samples, expression was normalised using FPKM (Nosrmalization), which is the number of fragments from a gene per kilobase length per million fragments, and FPKM (Fragments Per Kilo bases per Million fragments). For Pair-End sequencing, there will be two Reads for each Fragments, and FPKM only counts the number of Fragments that can be matched to the same transcript by the two Reads.

2.20. Statistical analysis

Statistical analyses were performed using IBM SPSS 19.0 software. Data are expressed as mean ± SEM. Parametric tests were based on unpaired two-tailed Student's t-tests (for comparisons between two groups) or on one-way ANOVA followed by post-hoc tests (for comparisons between more than one group). The Kruskal-Wallis test followed by the Mann-Whitney U test was used for non-parametric tests of multiple comparisons. In all experiments, p-value <0.05 was statistically significant. n is the number of mice or humans per group, indicating the number of biologically independent samples. ImageJ (ver. 1.51j8) software was used to analyse the mean intensity of immunofluorescence. All graphs were created using

GraphPad Prism 9.0 software. All experiments were repeated at least three times. The sample size for each experiment was not predetermined by statistical methods. No data were excluded from the analyses. The experiments in this study were randomised. The experiments were blinded and the researchers were unaware of the identity of the animals and the identity of the research team.

Supplementary Table 1. Information of si-RNA target sequence

Species	Si-RNA	Target sequence
Human	siELF1-1	GGATGTTGCTGAAGAAGAA
Human	siELF1-2	GGCTGAGTAGTCAGTTATA
Human	siE2F3-1	CAAAGGTTAGCTTATGTTA
Human	siE2F3-2	CGAAGTCCAGATAGT CCAA
Human	siYTHDF2-1	GCACAGAAGTTGCAAGCAA
Human	si YTHDF2-2	GGAAGAAGAAAGTGTTAAA
Human	siMETTL3-1	GAGTGATATTTGTACAATA
Human	siMETTL3-2	GGTTGCACGGTTCAAGCAA
Norway rat	siElf1-1	GTCTTCAAGTCCAGGAATA
Norway rat	siElf1-2	CAGTAACACTCCAGACAGT
Norway rat	siE2f3-1	GGTTTACTTGTGTCCAGAA
Norway rat	siE2f3-2	CGAGGATTCAGAGAATCAA
Norway rat	siYthdf2-1	GGGATTGACTTCTCAGCAT
Norway rat	siYthdf2-2	GGGCTGATATTGCTAGCAA
Norway rat	siMettl3-1	GTCTATAGTCCCTGAATTA
Norway rat	siMettl3-2	CCTACAAGATGACGCACAT

Supplementary Table 2. Nucleotide sequences of gene-specific primers used for quantitative real-time reverse transcription PCR.

Gene name	Primer	Sequence of primers (5′ to 3′)
Human_ELF1	Forward	TGTCCAACAGAACGACCTAGT
	Reversed	GGCAGGAAAAATAGCTGGATCAC
Human_CCNA2	Forward	CGCTGGCGGTACTGAAGTC
	Reversed	GAGGAACGGTGACATGCTCAT
Human_CCNB1	Forward	AATAAGGCGAAGATCAACATGGC
	Reversed	TTTGTTACCAATGTCCCCAAGAG
Human_CCNE2	Forward	TCAAGACGAAGTAGCCGTTTAC

	Reversed	TGACATCCTGGGTAGTTTTCCTC
Human_CDK1	Forward	AAACTACAGGTCAAGTGGTAGCC
	Reversed	TCCTGCATAAGCACATCCTGA
Human_CDK2	Forward	CCAGGAGTTACTTCTATGCCTGA
	Reversed	TTCATCCAGGGGAGGTACAAC
Human_CDK4	Forward	ATGGCTACCTCTCGATATGAGC
	Reversed	CATTGGGGACTCTCACACTCT
Human_COL2A1	Forward	TGGACGATCAGGCGAAACC
	Reversed	GCTGCGGATGCTCTCAATCT
Human_CCND1	Forward	GCTGCGAAGTGGAAACCATC
	Reversed	CCTCCTTCTGCACACATTTGAA
Human_ADAMTS5	Forward	GAACATCGACCAACTCTACTCCG
	Reversed	CAATGCCCACCGAACCATCT
Human_MMP13	Forward	ACTGAGAGGCTCCGAGAAATG
	Reversed	GAACCCCGCATCTTGGCTT
Human_CDKN2A(P16)	Forward	GATCCAGGTGGGTAGAAGGTC
	Reversed	CCCCTGCAAACTTCGTCCT
Human_METTL3	Forward	TTGTCTCCAACCTTCCGTAGT
	Reversed	CCAGATCAGAGAGGTGGTGTAG
Human_E2F3	Forward	AGAAAGCGGTCATCAGTACCT
	Reversed	TGGACTTCGTAGTGCAGCTCT
Human_CDKN1A(P21)	Forward	TGTCCGTCAGAACCCATGC
	Reversed	AAAGTCGAAGTTCCATCGCTC
Human_YTHDF2	Forward	AGCCCCACTTCCTACCAGATG
	Reversed	TGAGAACTGTTATTTCCCCATGC
Mouse_Elf1	Forward	TGTCCAACAGAACGACCTAGT
	Reversed	ACACAAGCTAGACCAGCATAAC
Mouse_Mettl3	Forward	CTGGGCACTTGGATTTAAGGAA
	Reversed	TGAGAGGTGGTGTAGCAACTT
Mouse_Ythdf2	Forward	GAGCAGAGACCAAAAGGTCAAG
	Reversed	CTGTGGGCTCAAGTAAGGTTC
Mouse _Cdkn1a (p21)	Forward	CCTGGTGATGTCCGACCTG
	Reversed	CCATGAGCGCATCGCAATC
Mouse _Cdkn2a (p16)	Forward	CGCAGGTTCTTGGTCACTGT
	Reversed	TGTTCACGAAAGCCAGAGCG
Mouse _E2f3	Forward	CAGATCCTCACTACGAACCCT
	Reversed	GTTCCAGCCTTCGCTTTGC
Mouse _Adamts5	Forward	CCCAGGATAAAACCAGGCAG
	Reversed	CGGCCAAGGGTTGTAAATGG
Mouse _Col2a1	Forward	GGGTCACAGAGGTTACCCAG
	Reversed	ACCAGGGGAACCACTCTCAC
Mouse _Ccnd1	Forward	GCGTACCCTGACACCAATCTC
	Reversed	ACTTGAAGTAAGATACGGAGGGC

Mouse _Ccne2	Forward	ATGTCAAGACGCAGCCGTTTA
	Reversed	GCTGATTCCTCCAGACAGTACA
Mouse _Cdk4	Forward	ATGGCTGCCACTCGATATGAA
	Reversed	TGCTCCTCCATTAGGAACTCTC
Rat_Elf1	Forward	TGTTCCGCTGTCACTTAGCTT
	Reversed	TCCCCCTTCACCCACAGTAA
Rat_Mettl3	Forward	TGCAGACCGACTCTTTCCAC
	Reversed	ATCCAGTTGGGCTGCACATT
Rat_Ythdf2	Forward	CAGGCAAGGCCGAATAATGC
	Reversed	TTTGCCACAGGACCCTTGTT
