# Supplementary material for: ELF1-mediated transactivation of METTL3/YTHDF2 promotes nucleus pulposus cell senescence via m6A-dependent destabilization of E2F3 mRNA in intervertebral disc degeneration
Source: Cell Death Discov. 2025 Jun 4;11:267. doi: 10.1038/s41420-025-02515-8 (PMC12137937; doi:10.1038/s41420-025-02515-8)

Each band of protein Marker indicated molecular weight (kDa) :

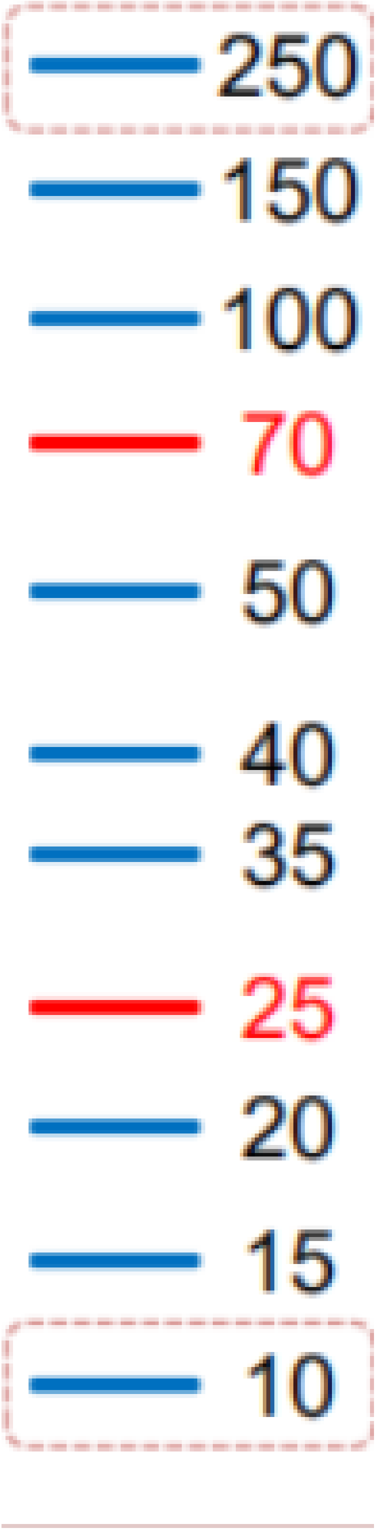

Figure-1F

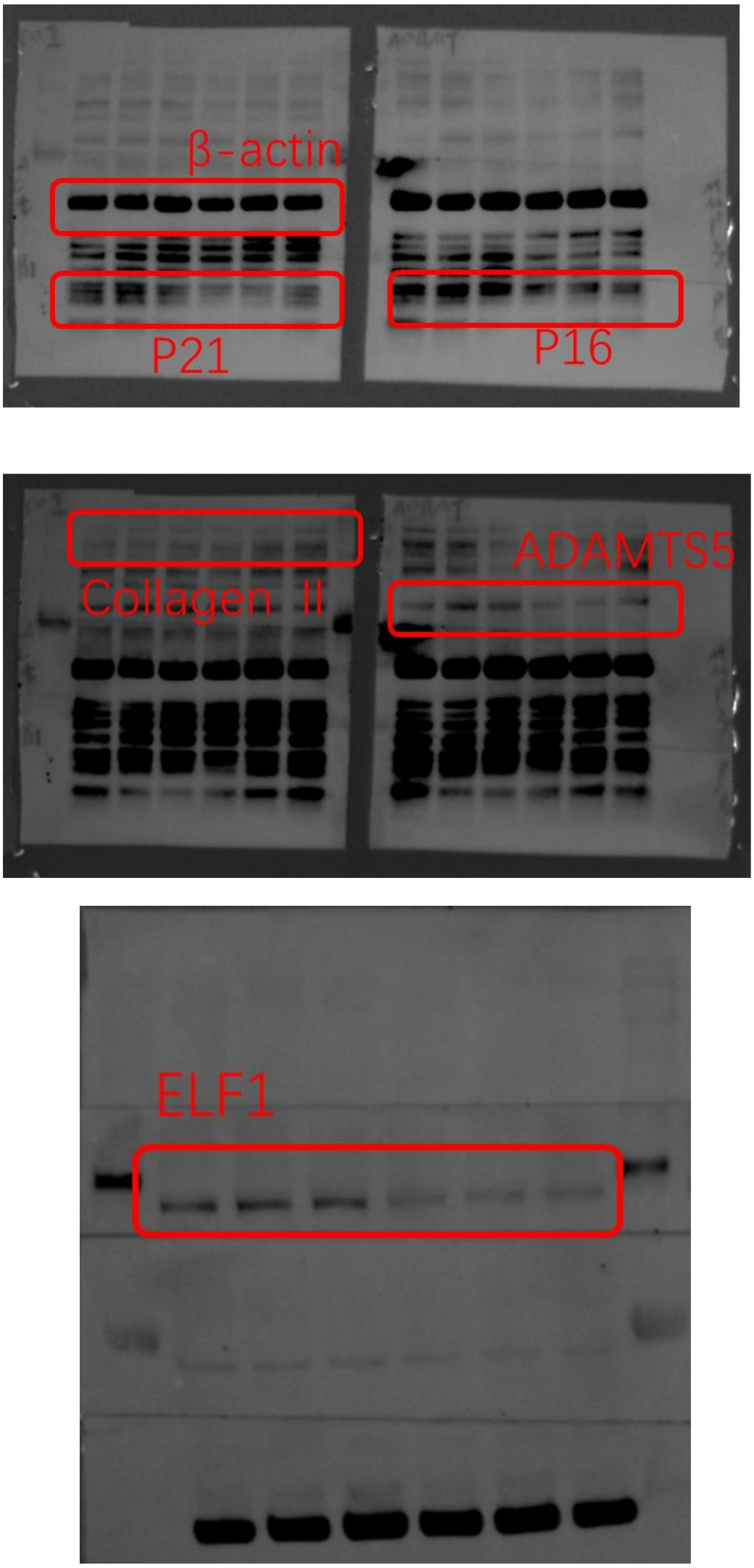

Figure-1P

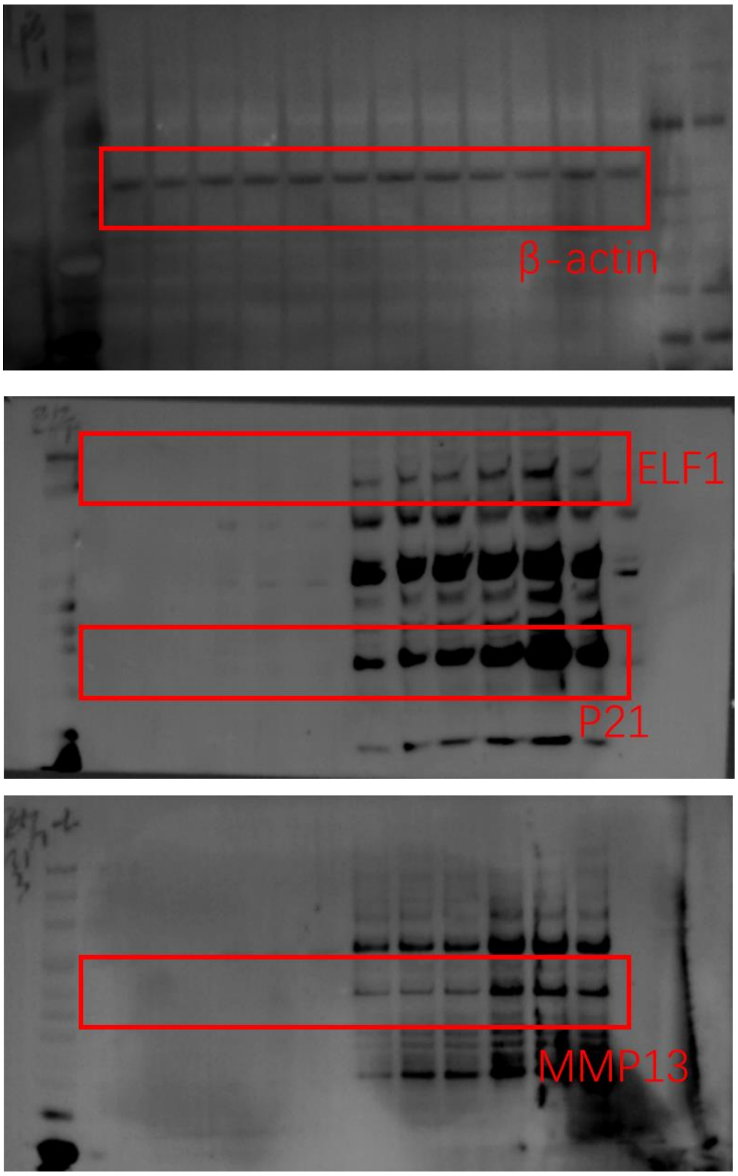

Figure-1Q

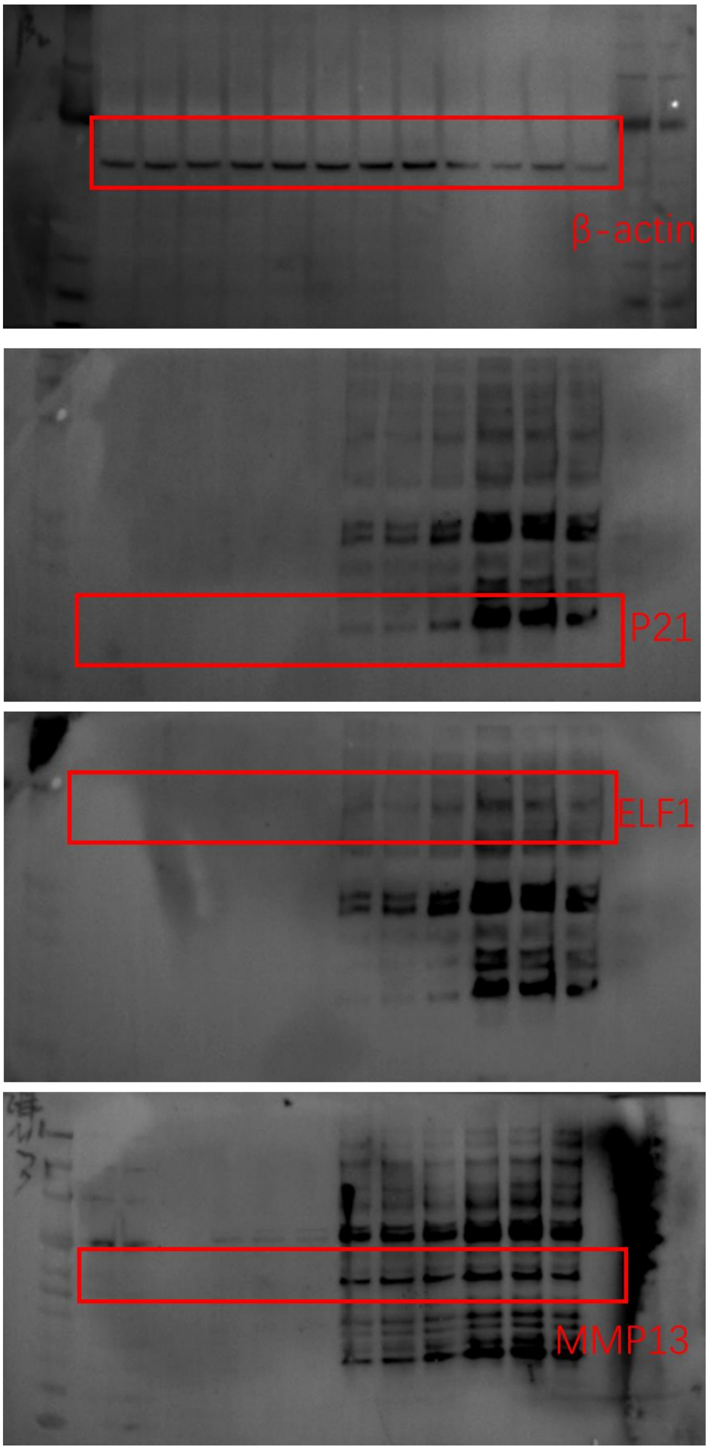

Figure-1R

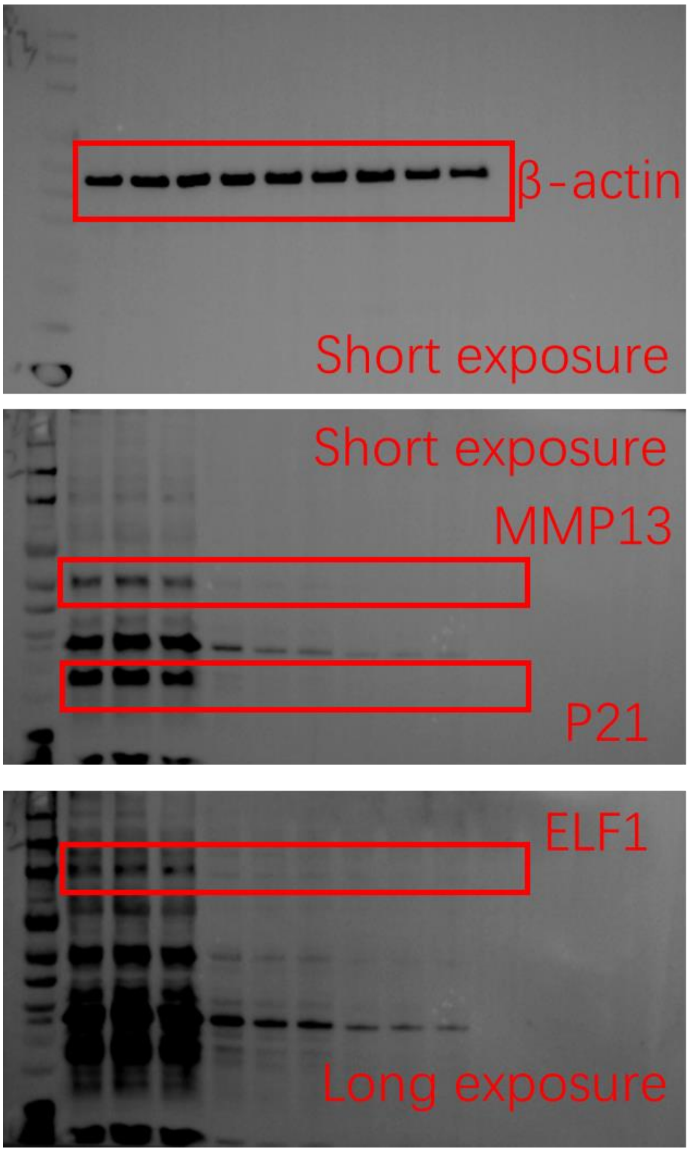
β-actin
Short exposure
Short exposure
MMP13
P21
ELF1
Long exposure

Figure-2E

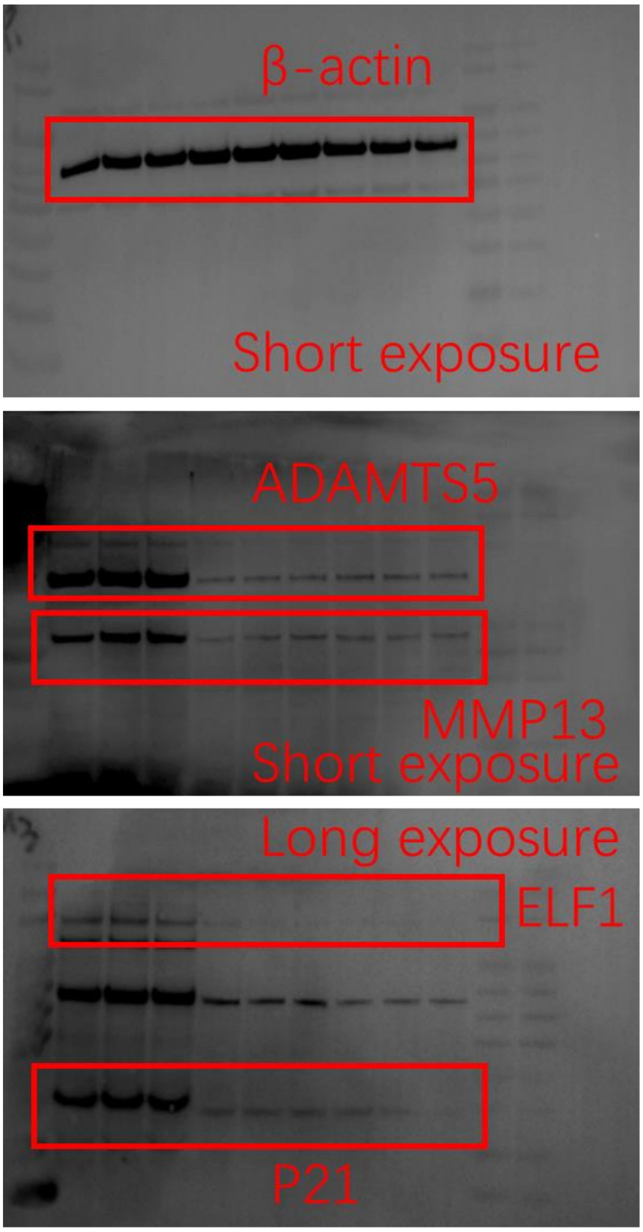

β-actin
Short exposure
ADAMTS5
MMP13
Short exposure
Long exposure
ELF1
P21

Figure-2O

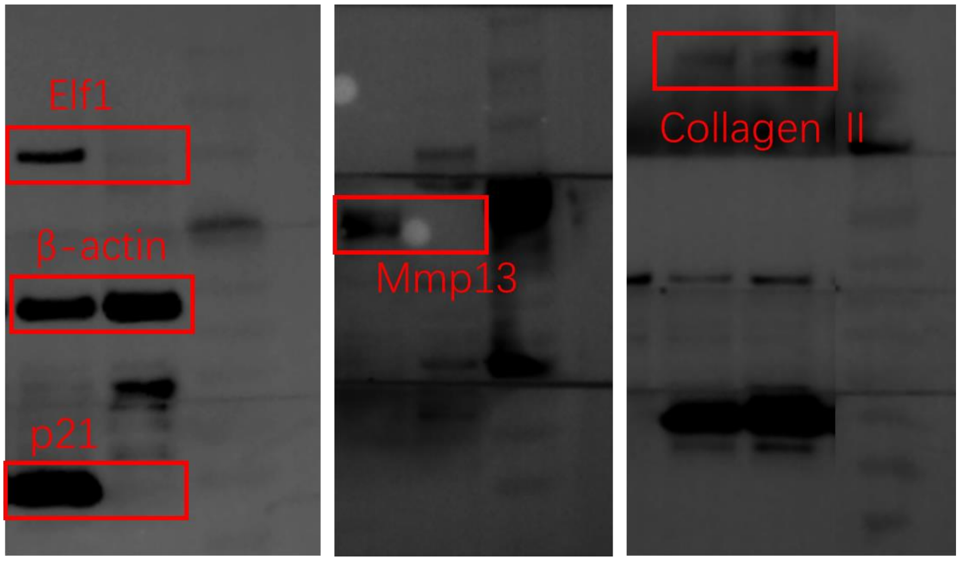

Figure-3N

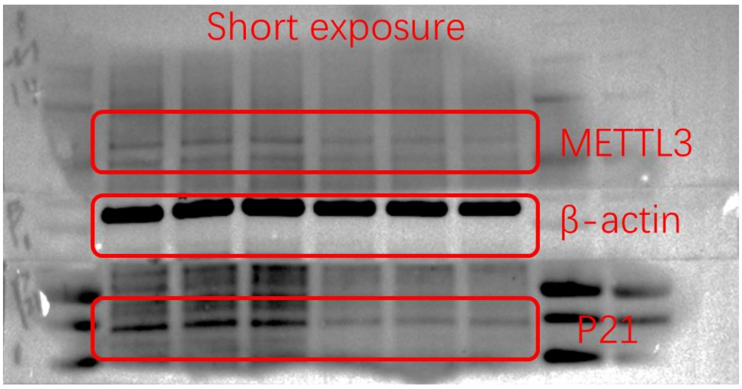

Figure-3R

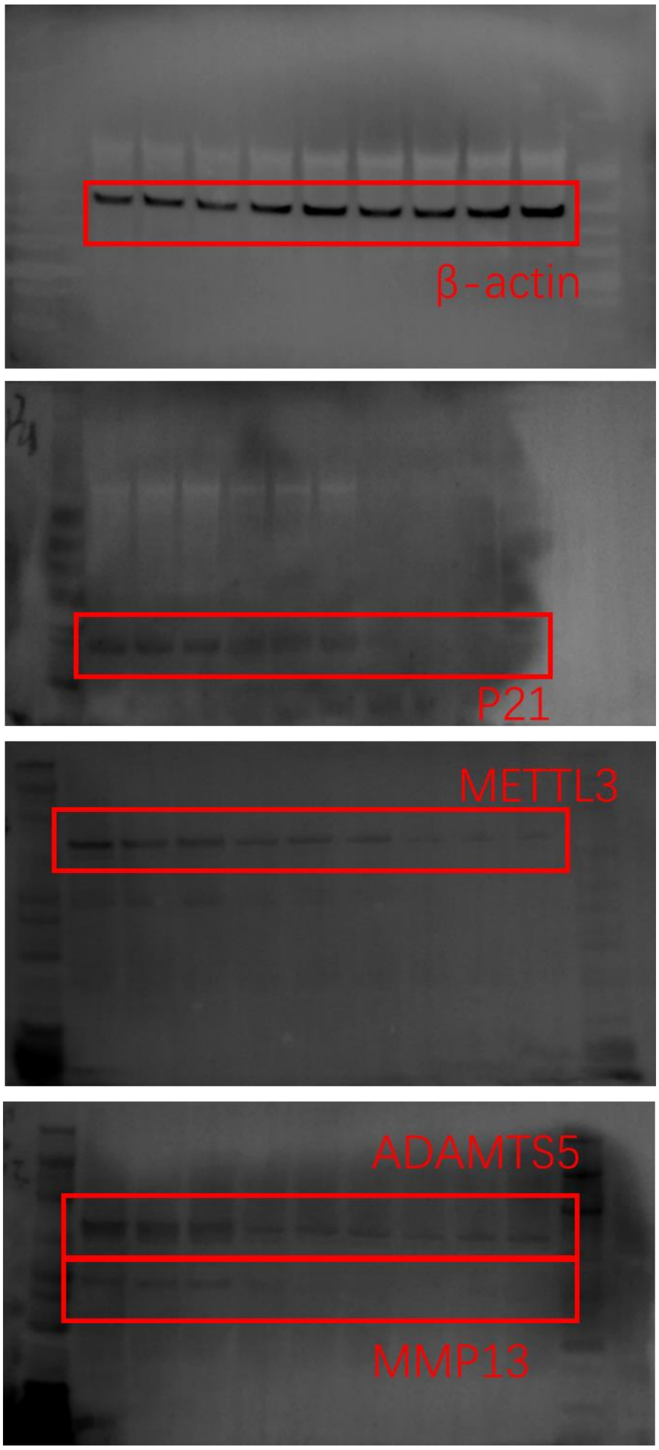

Figure-4C

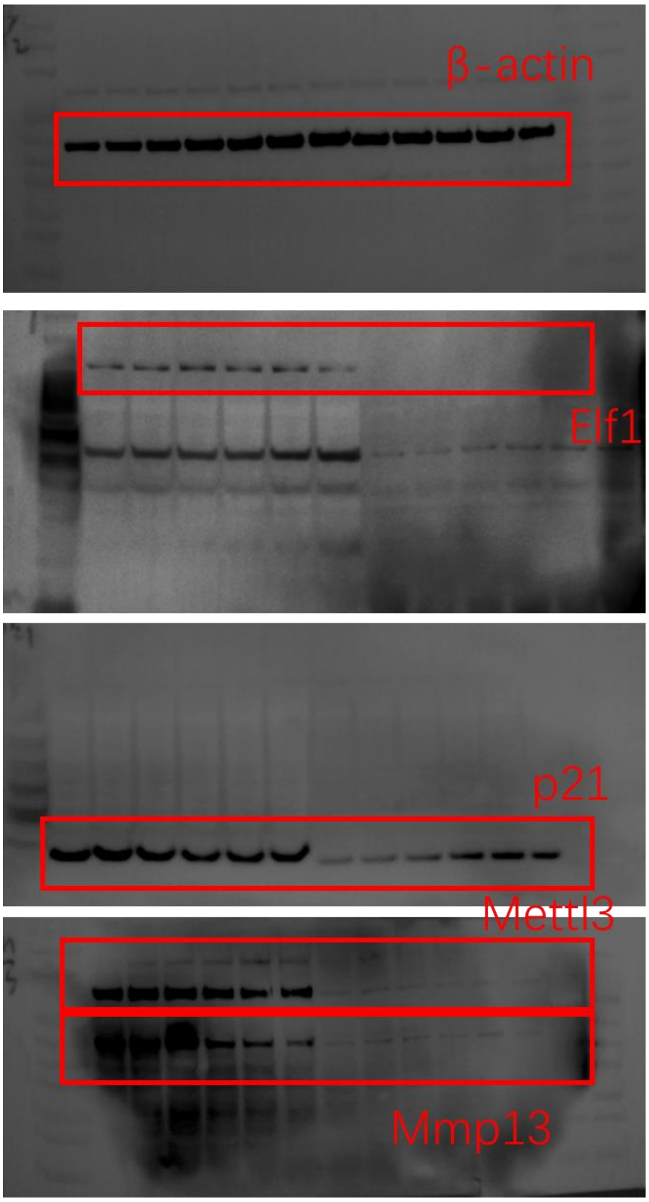

Figure-4D

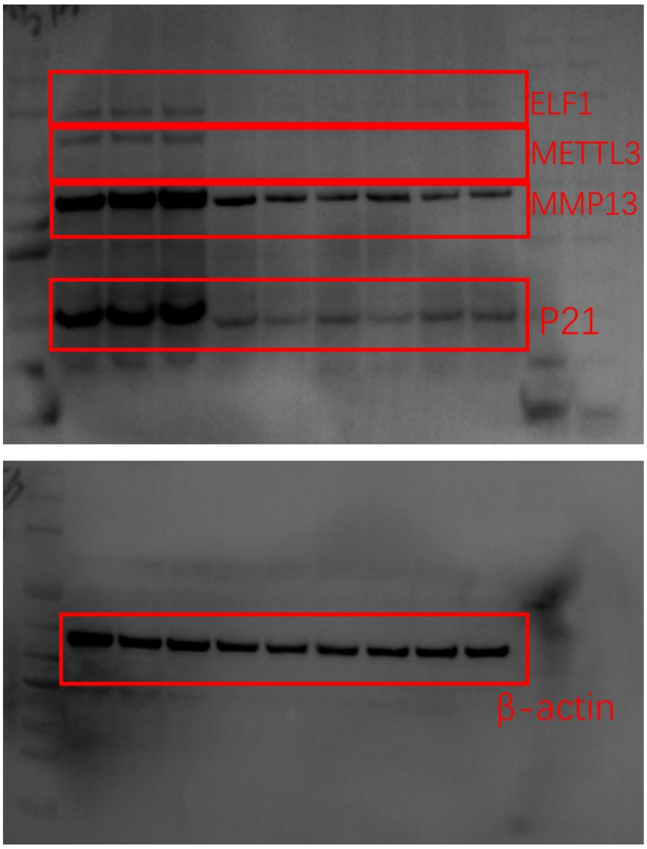

Figure-4N

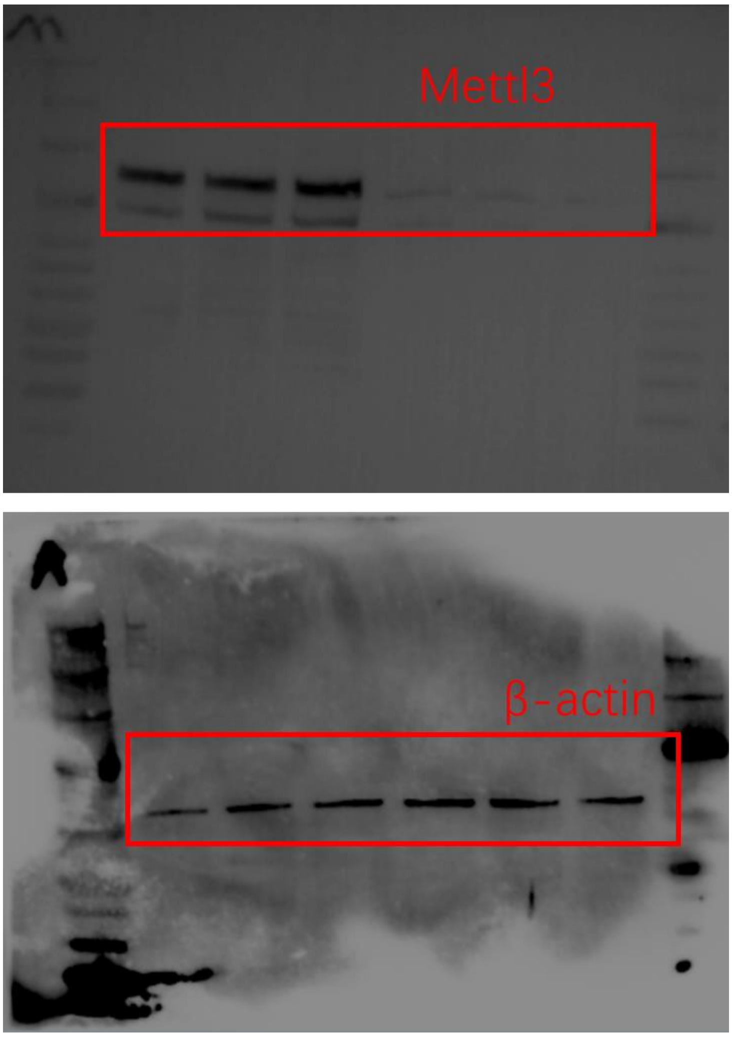

Figure-5E

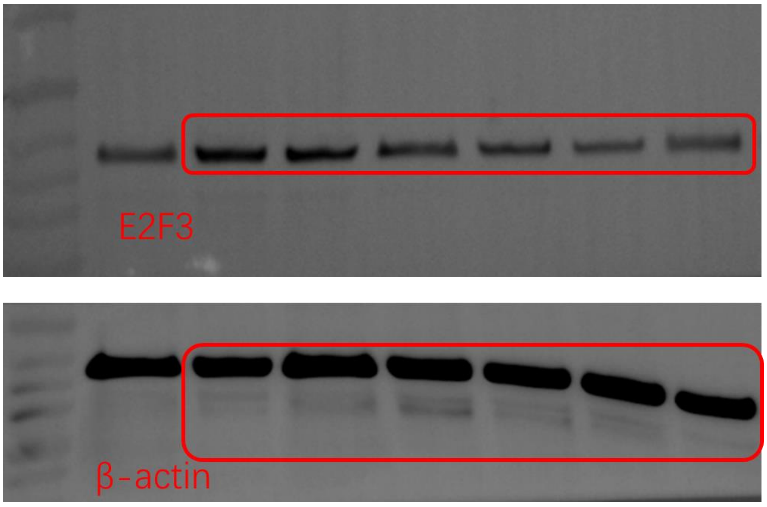

Figure-5F

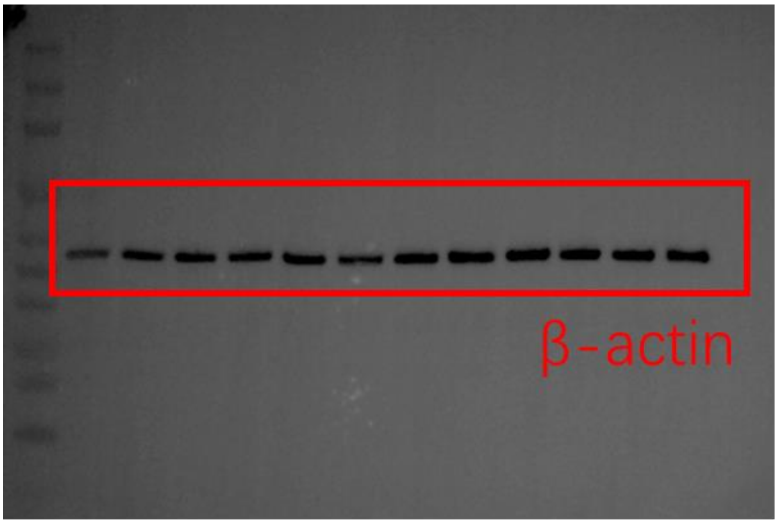

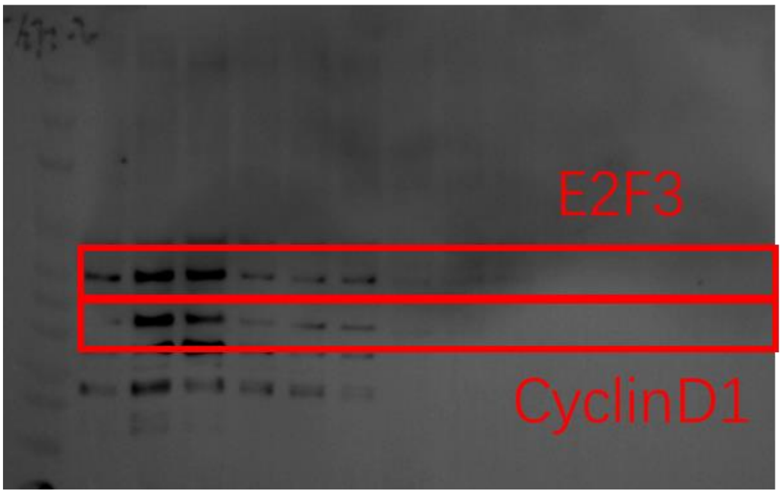

Figure-5I

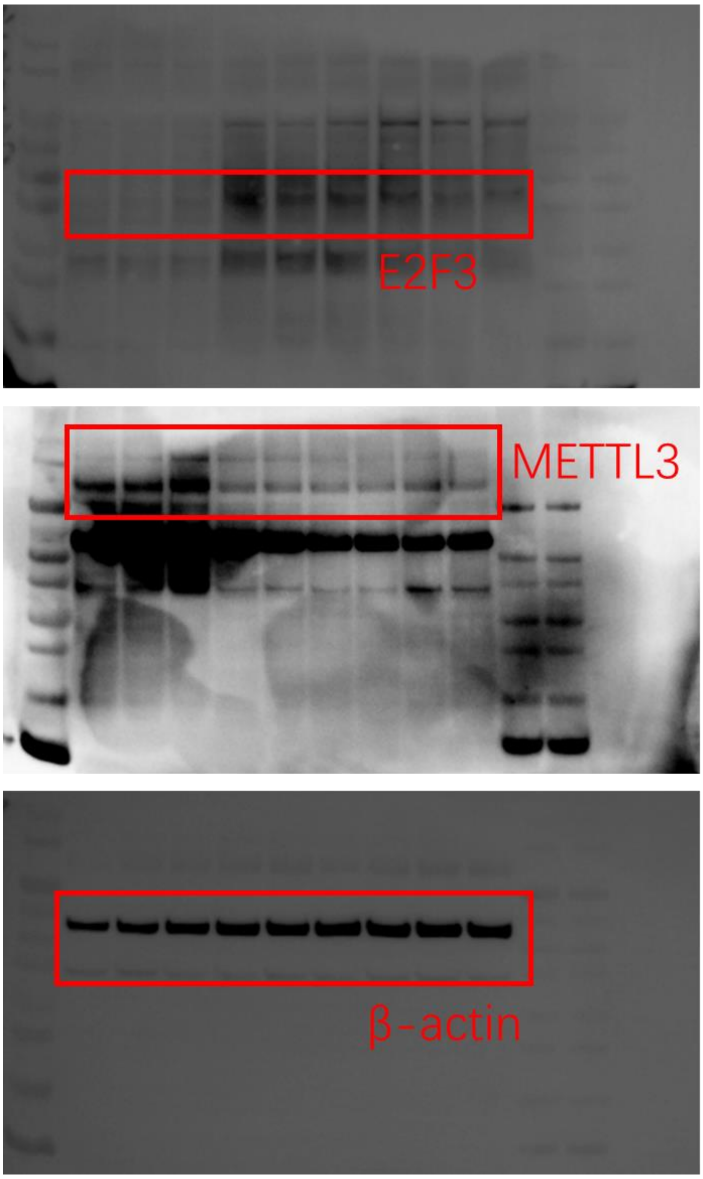

Figure-5J

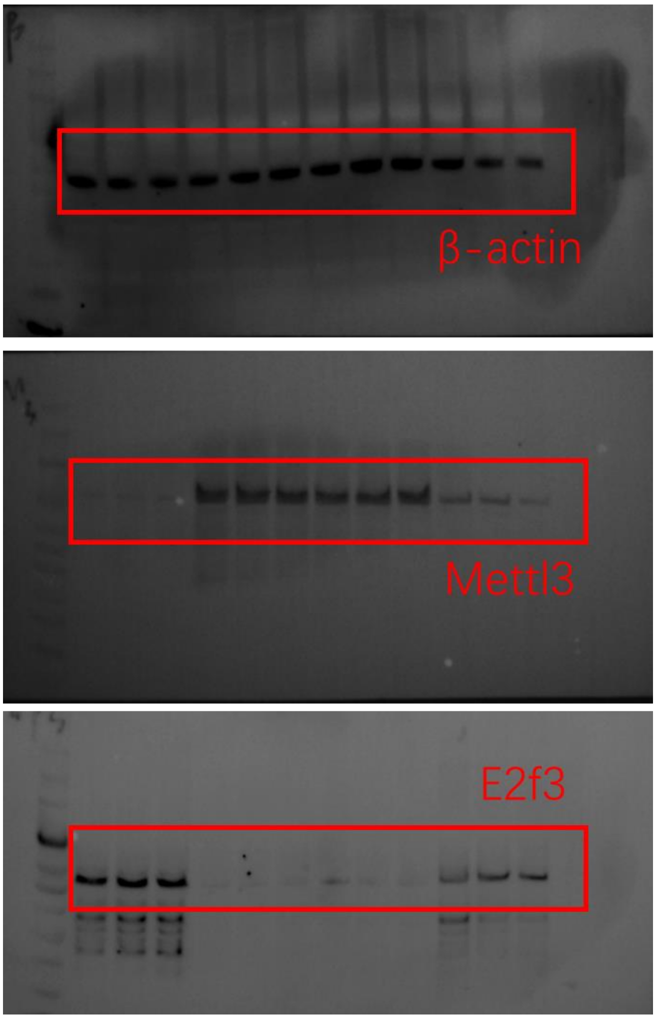

Figure-5L

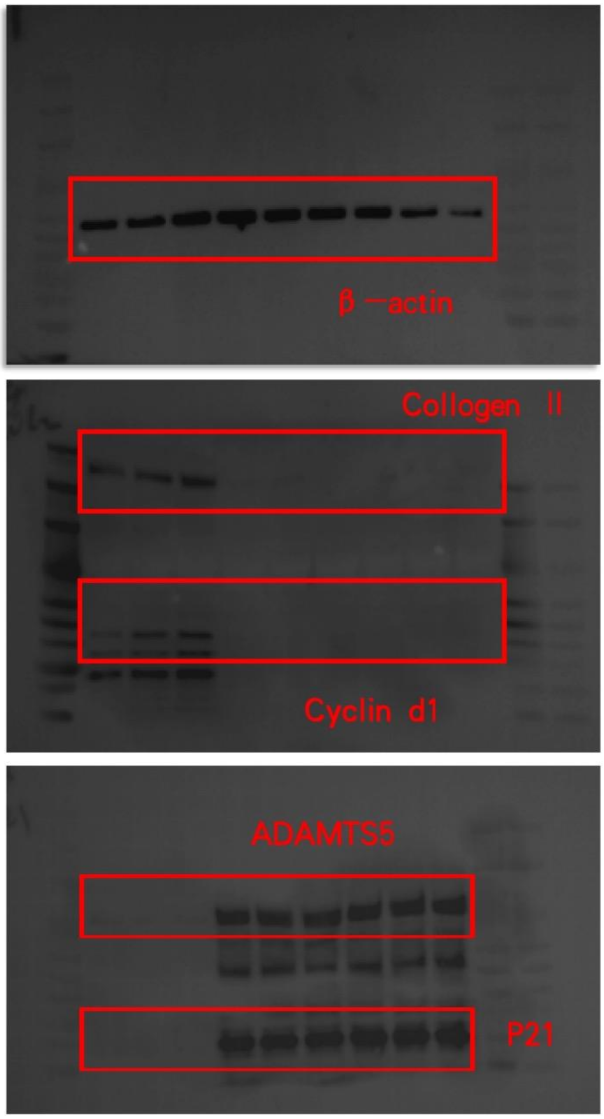

Figure-6L

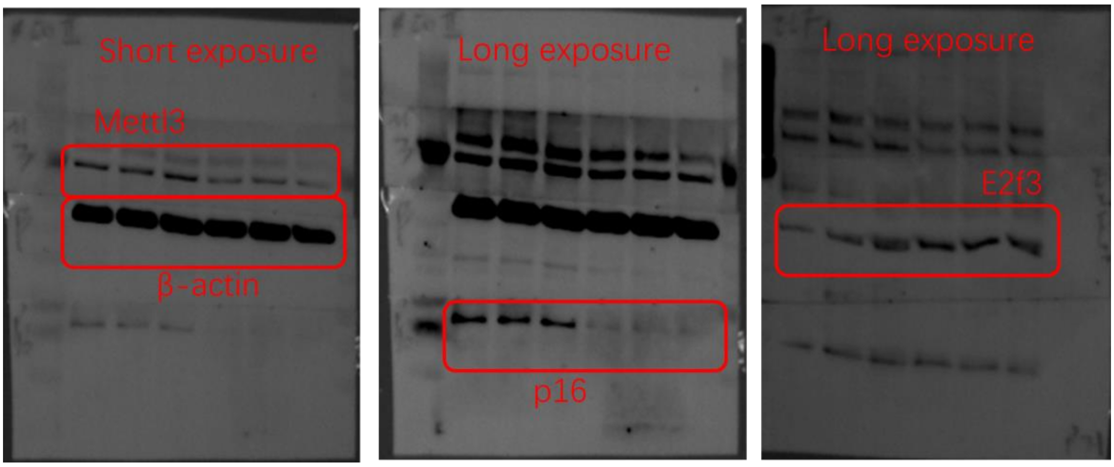

Figure-6S

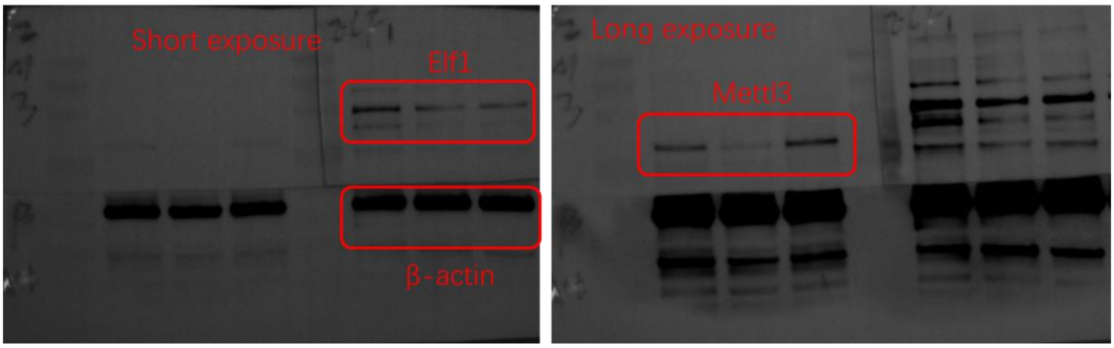

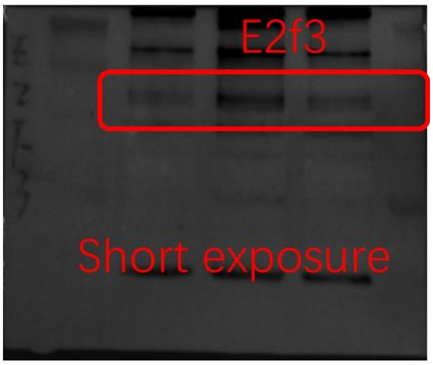

Figure-7F

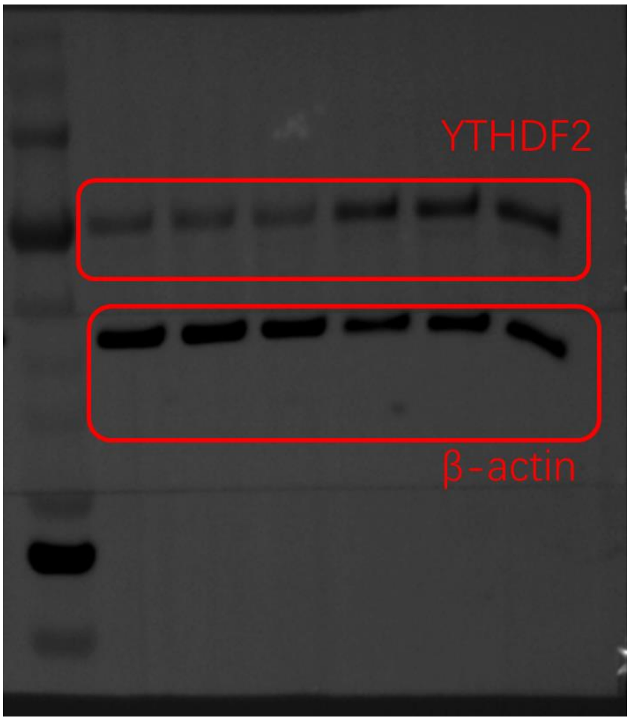

Figure-7K

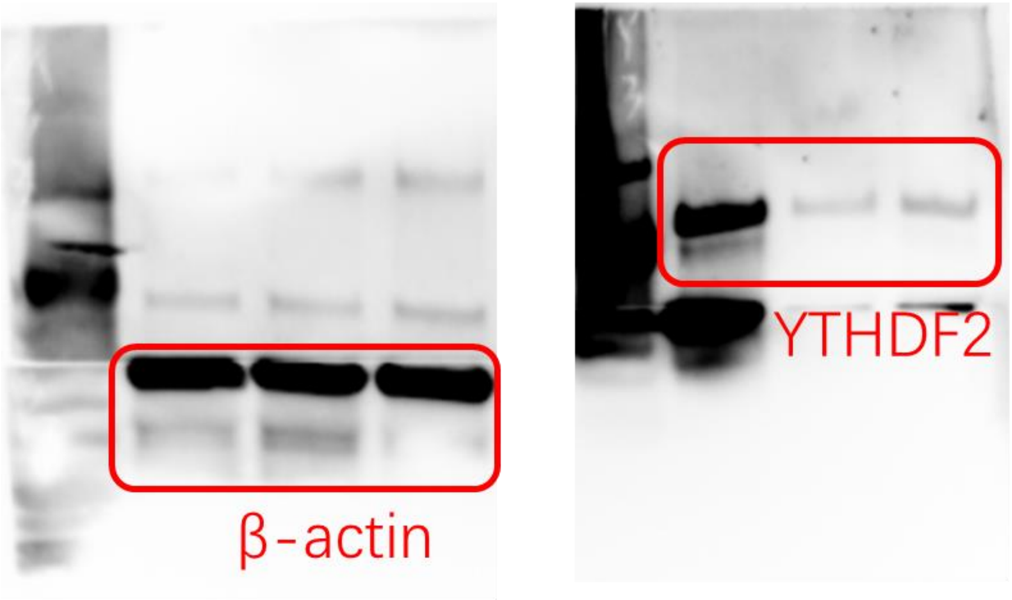

Figure-7T

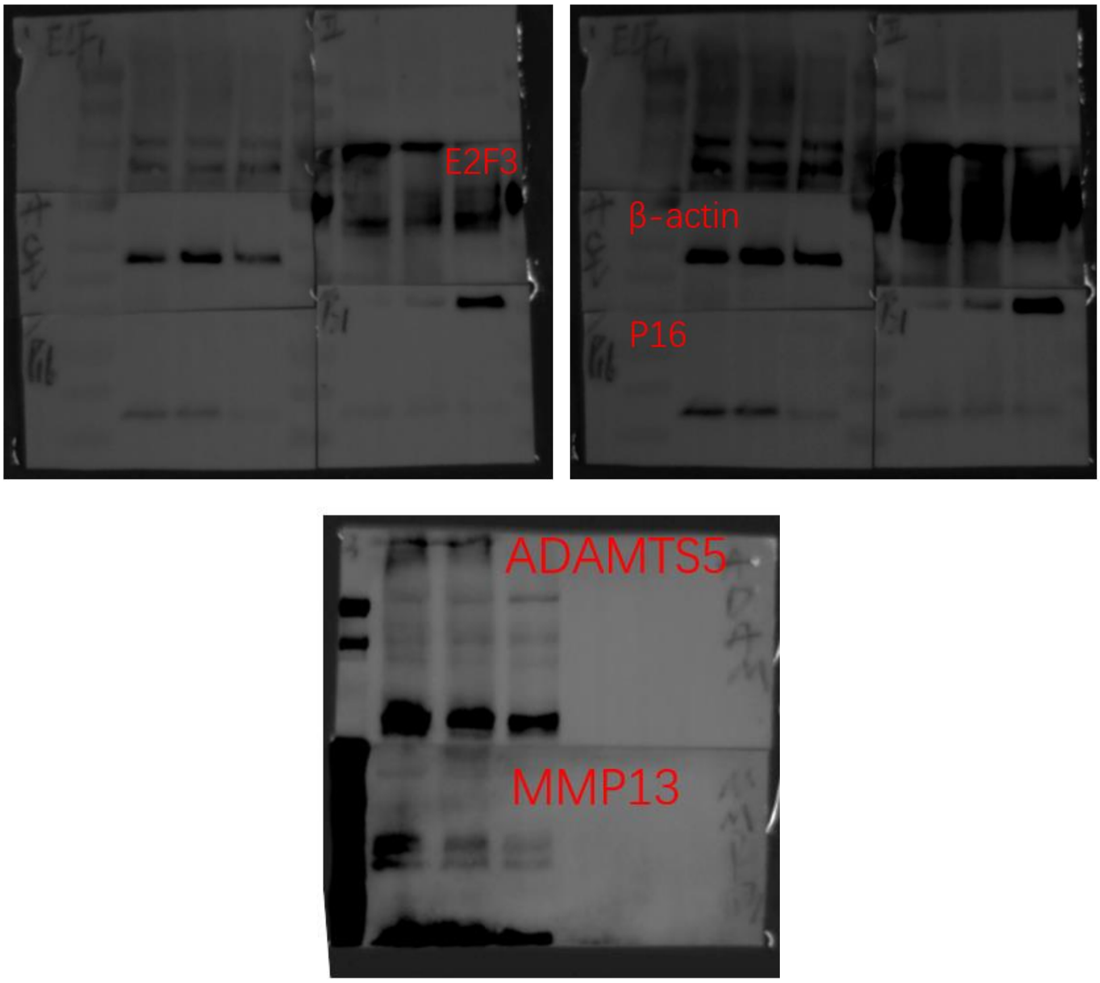

Figure-7X

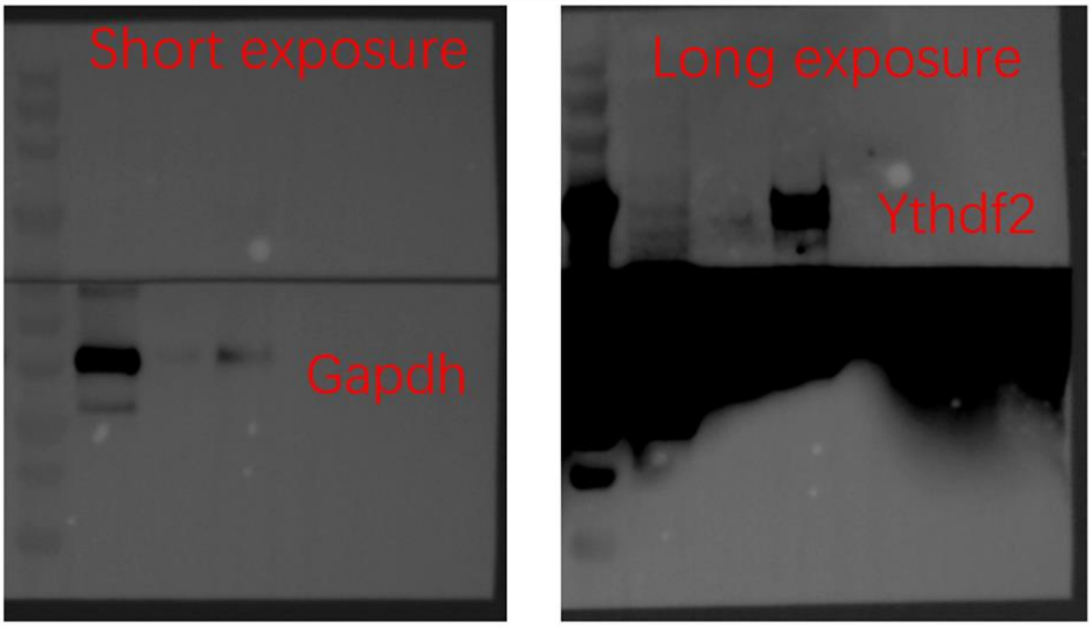

Figure-7Y

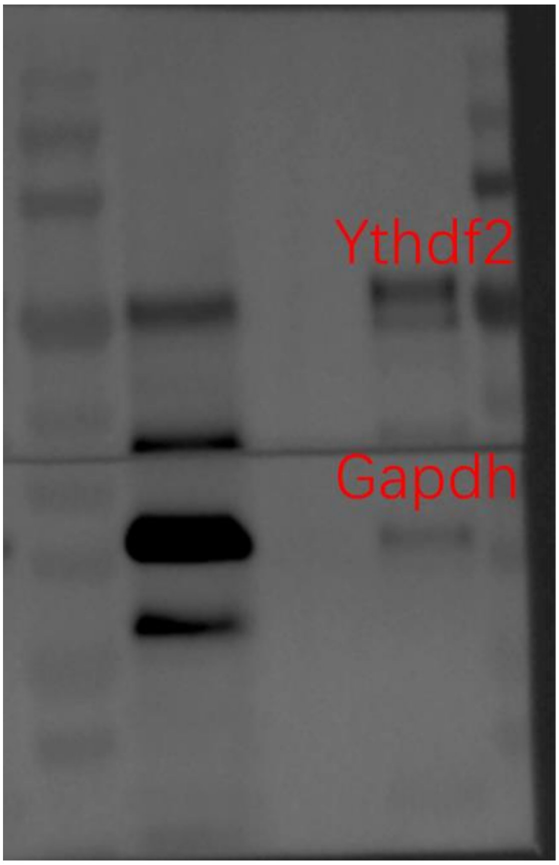

Figure-8B

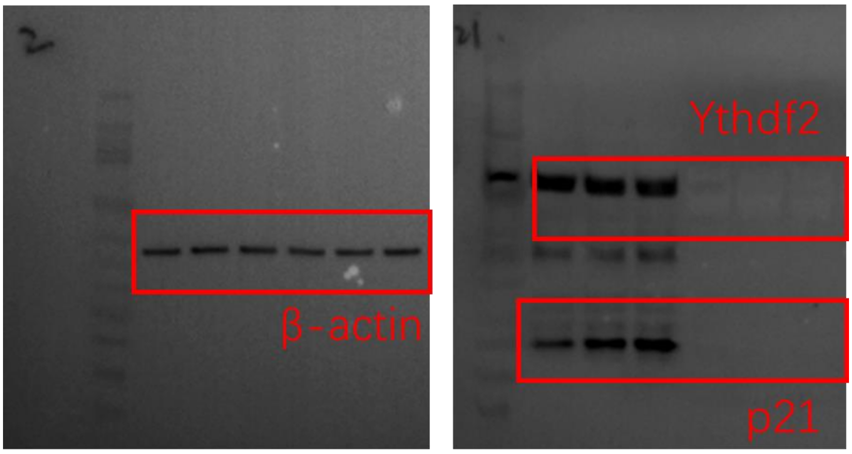

Figure-8D

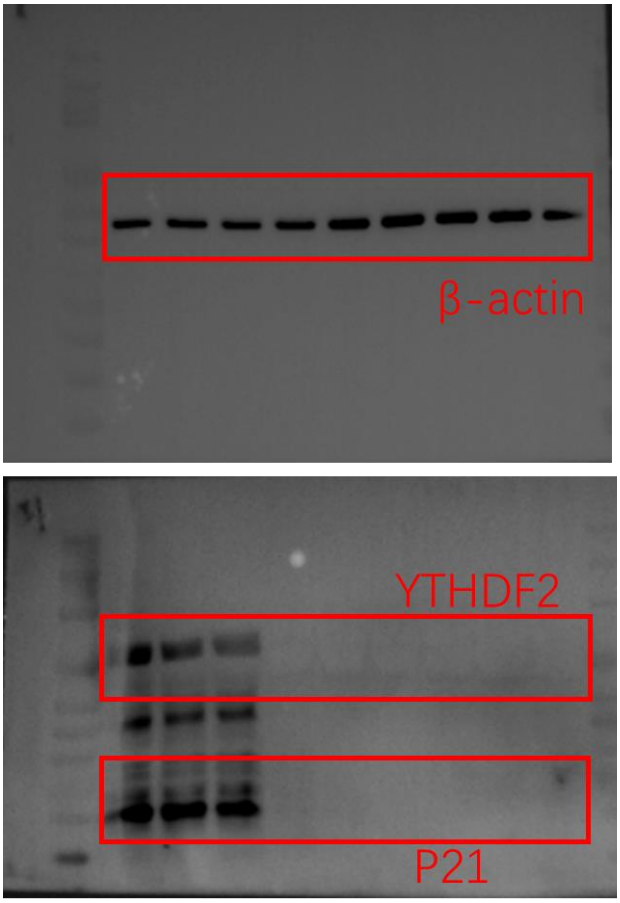

Figure-8K

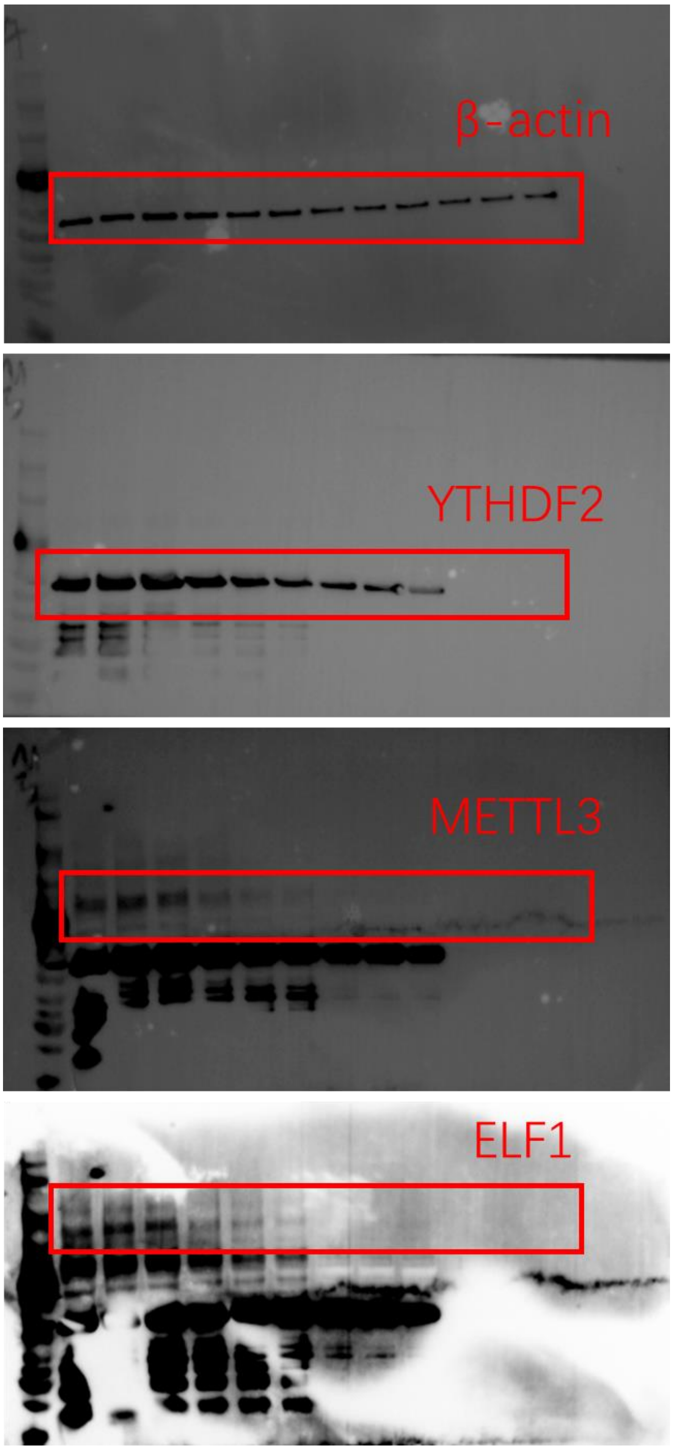

Figure-8N

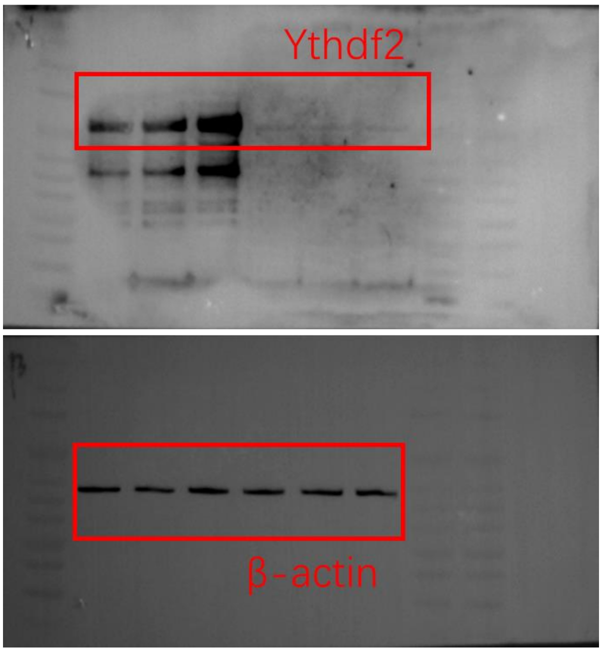

Figure-9I

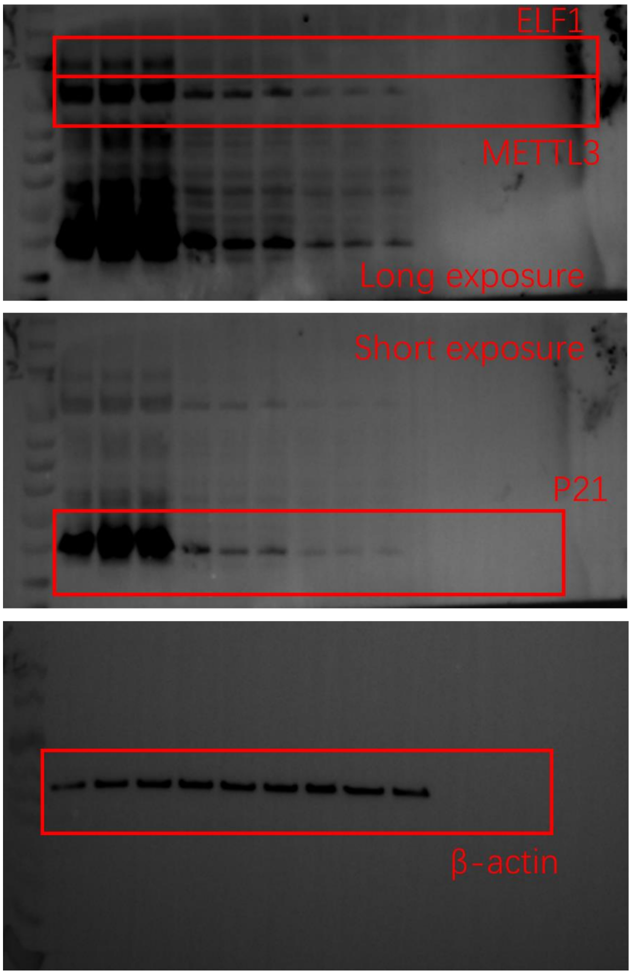

Figure-9J

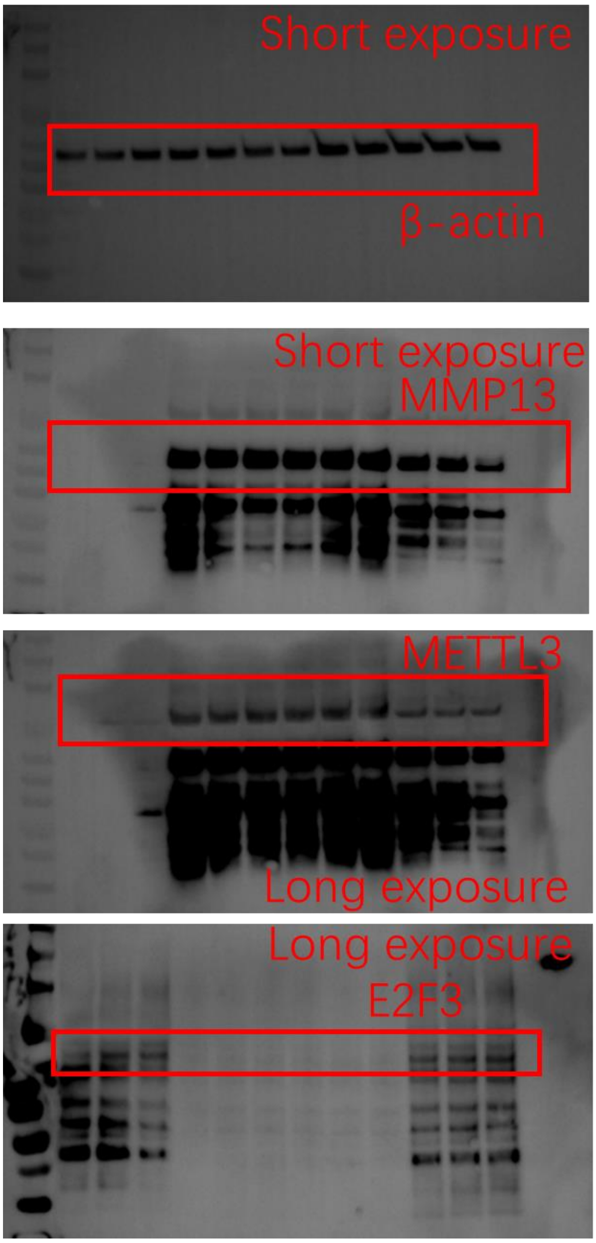
Short exposure
β-actin
Short exposure
MMP13
METTL3
Long exposure
Long exposure
E2F3

Figure-10B

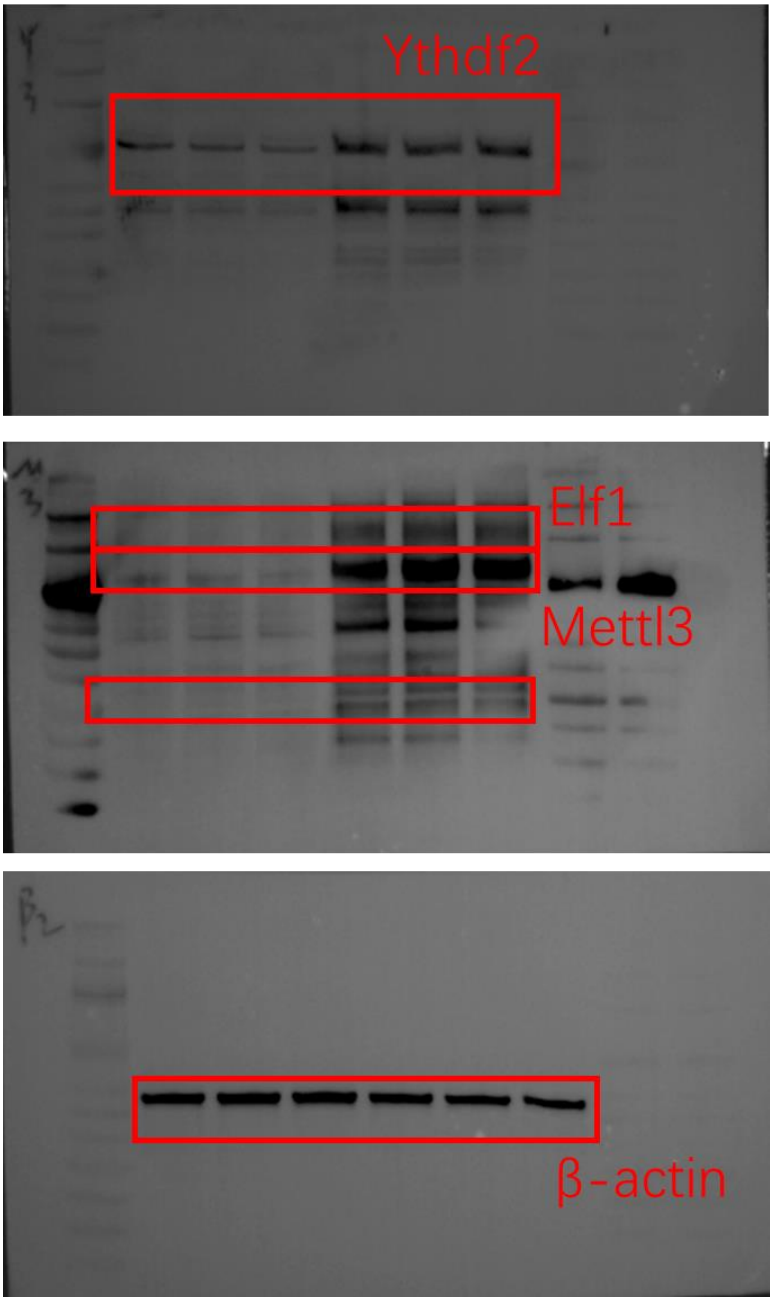

Figure-10L

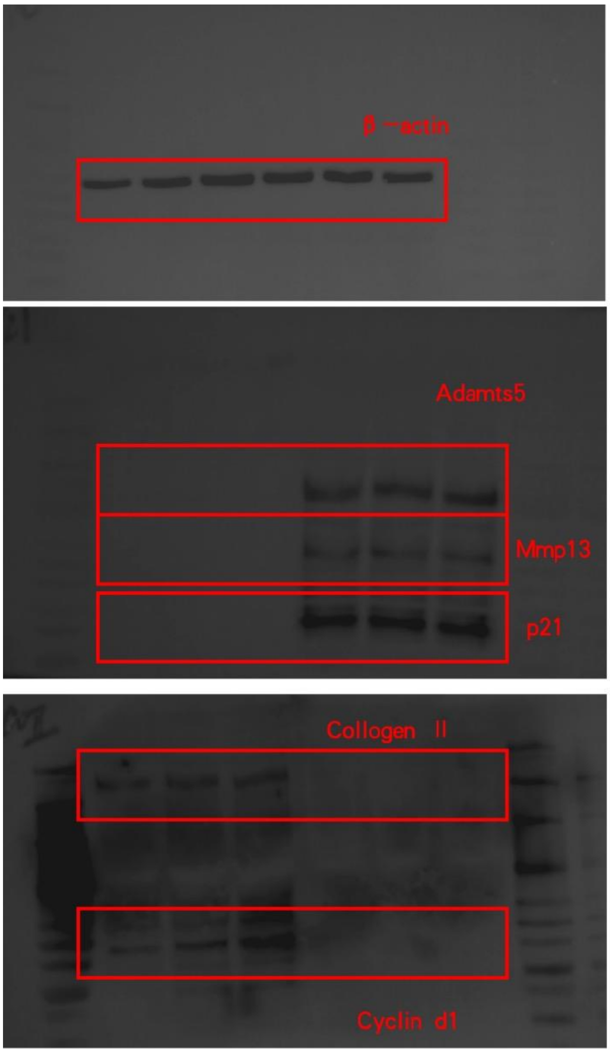

Figure-10M

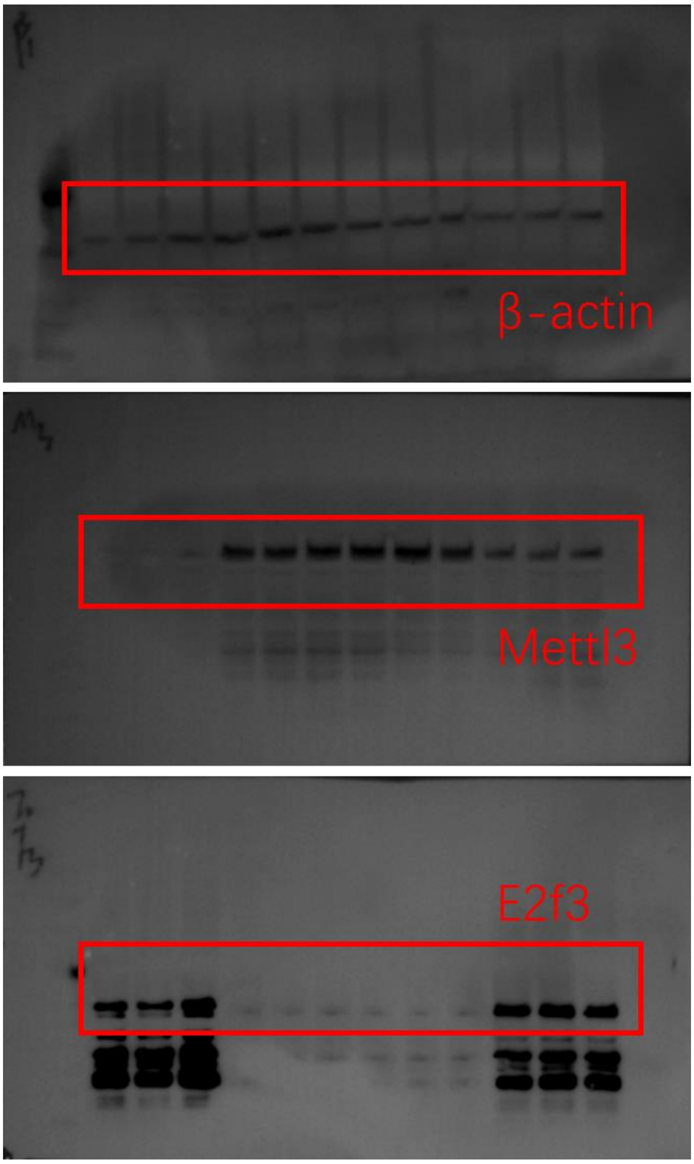

Supplementary Figure-8D

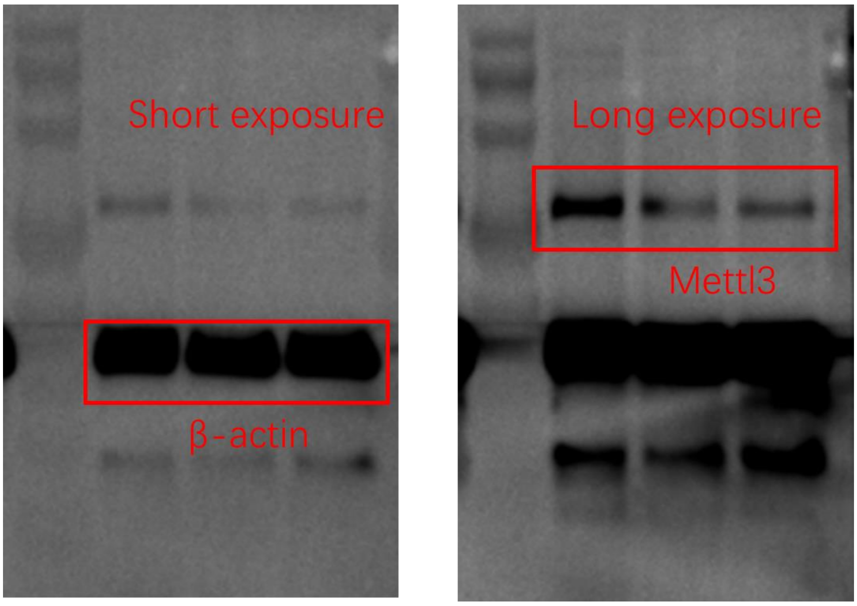

Supplementary Figure-8E

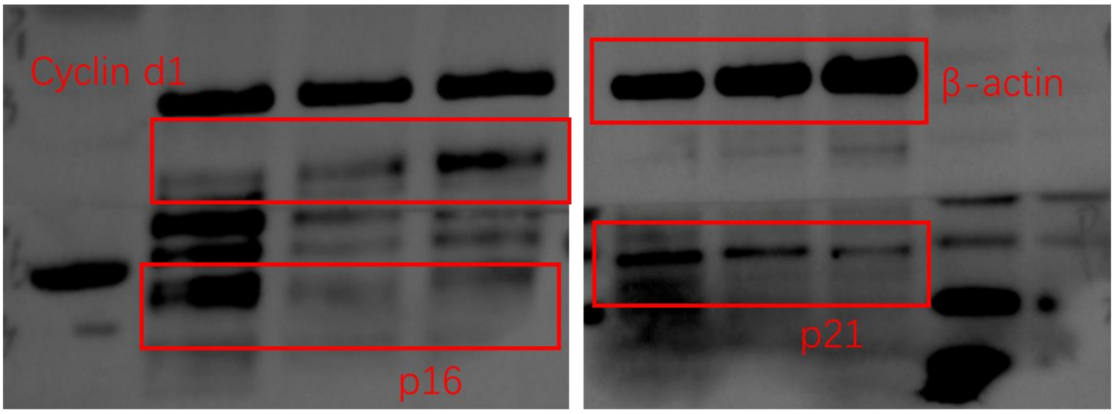

Supplementary Figure-12B

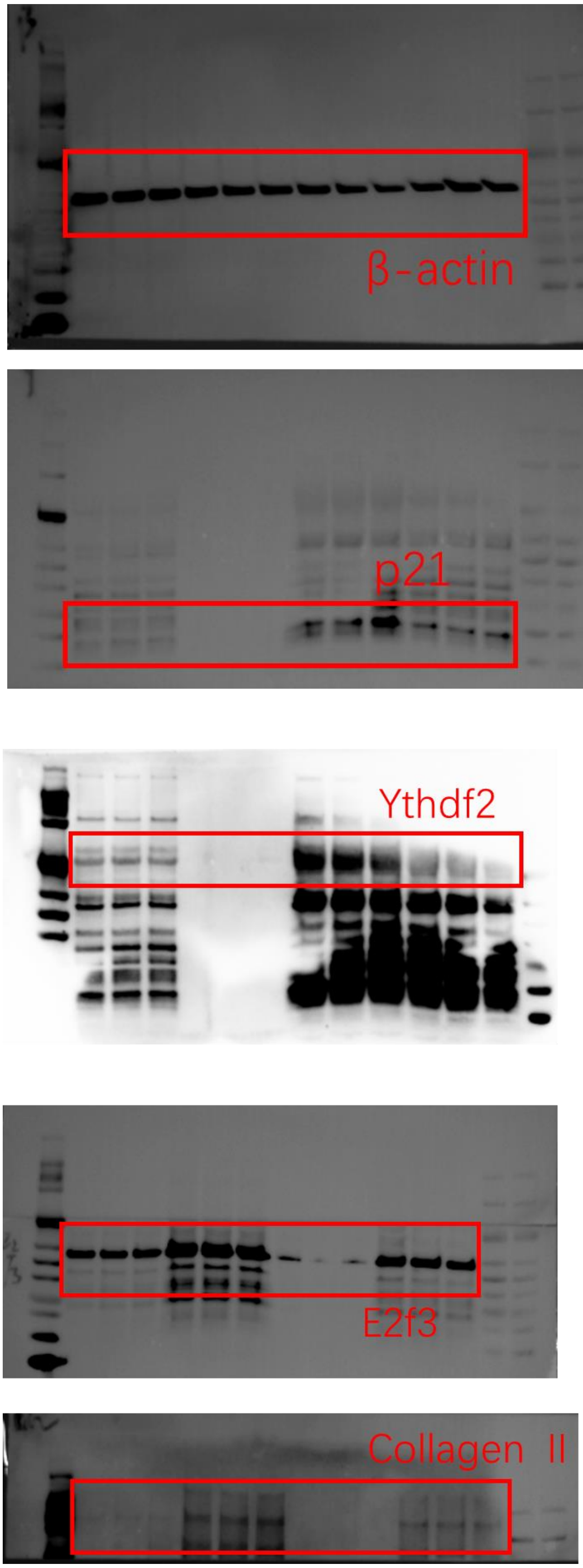

Supplementary Figure-12C

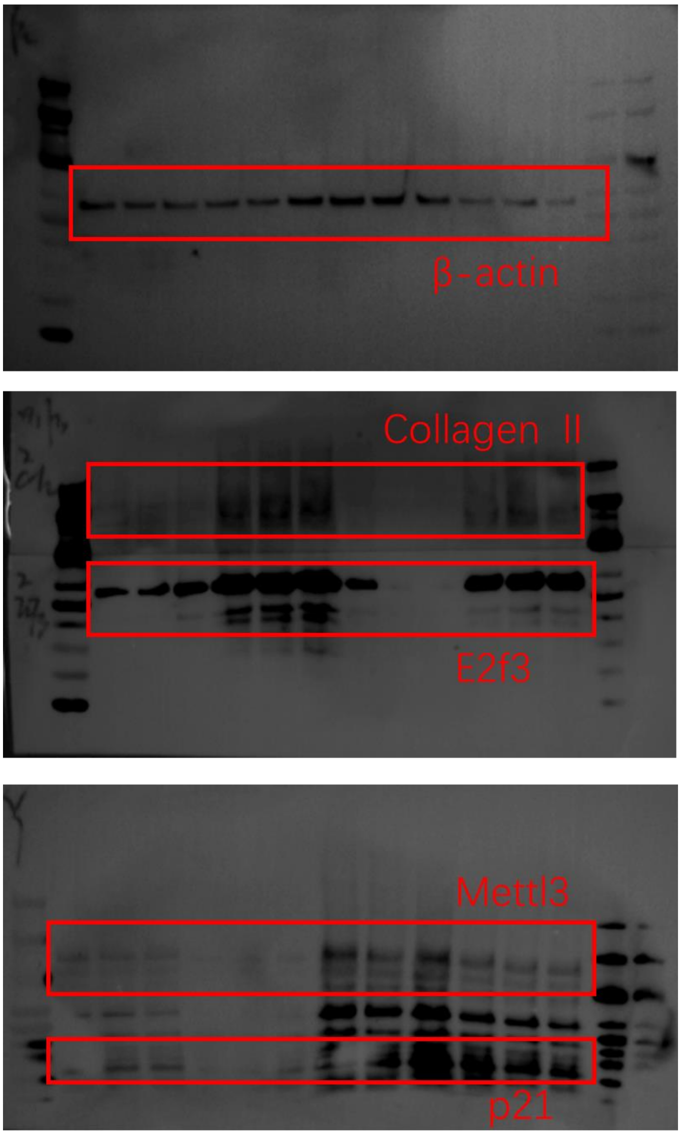

Supplement: Supplementary file 2 — Full and uncropped western blots [file 41420_2025_2515_MOESM2_ESM.pdf]
